# Simple and Scalable Electrochemical Reduction of Alkyl Oxalates Using Carbon‐Based Electrodes

**DOI:** 10.1002/cssc.202502557

**Published:** 2026-03-22

**Authors:** Sebastian Kissel, Philipp Schnieders, Volker Derdau, Siegfried R. Waldvogel

**Affiliations:** ^1^ Department of Electrosynthesis Max‐Planck‐Institute for Chemical Energy Conversion, Mülheim an der Ruhr Germany; ^2^ Deutero GmbH Kastellaun Germany; ^3^ Sanofi R&D, Isotope Chemistry Integrated Drug Discovery Frankfurt Germany; ^4^ Karlsruhe Institute of Technology Institute of Biological and Chemical Systems – Functional Molecular Systems (IBCS‐FMS) Karlsruhe Germany

**Keywords:** carbonyl reduction, deuteration, electrolysis, electroreduction, electrosynthesis, ester reduction

## Abstract

In this study, a simple and robustly scalable electrochemical method for the reduction of alkyl oxalates was established. The use of electricity as a universal agent for reduction applications avoids the stoichiometric formation of reagent waste and the use of transition metal catalysts. In addition, employing readily available carbon electrode materials like glassy carbon or graphite provide important prerequisites for later technical applications. This goes along with common solvents such as acetonitrile and acetic acid in combination with a flow electrolysis setup, allowing easy scalability. The scale‐up experiments on up to a 120 mmol scale proved the practical relevance of the method and the potential use for industrial large‐scale synthesis. The method is suitable for a variety of esters, 14 examples up to 97% yield, and the simple deuterium incorporation to obtain highly deuterated alkyl glycolates.

## Graphical abstract

A method for the electrochemical reduction of alkyl oxalates under mild and ambient conditions in a very simple two‐electrode undivided setup under galvanostatic conditions is established. A simple isolation by direct distillation of the crude mixture in combination with the reusability of the electrodes provides all prerequisites for potential technical applications.

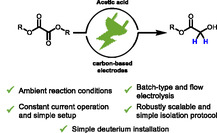

## 1 Introduction

**SCHEME 1:**
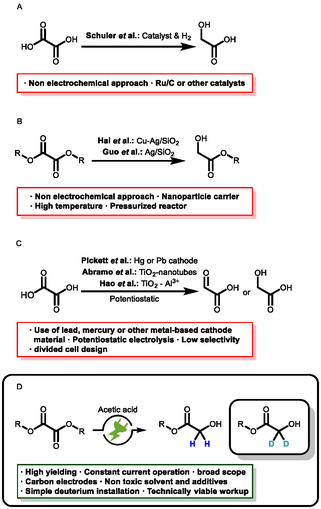
Different methods for the reduction of alkyl oxalates to alkyl glycolates. (A) Coventional reduction of oxalic acid. (B) Selective reduction of oxalates. (C) Electrochemical reduction of oxalic acid. (D) This work: selective electrochemical reduction of oxalates.

Glycolates are a versatile motif in chemical compounds that are commonly made from glycolic acid with various alcohols and have a broad field of application [1, 2]. In cosmetics, glycolates are often used for their exfoliating and moisturizing properties [3, 4, 5]. As such, they are an essential ingredient in skin care that aims to improve the appearance and reduce signs of ageing [6]. In pharmaceutical applications, they are active ingredient carriers that can increase the solubility and bioavailability of drugs [7, 8]. Recently, glycolic acid and its esters have gained interest as a platform in plastics production, where they serve as plasticizers and solvents. Their ability to increase the flexibility and durability of plastics makes them a valuable additive and serves as a building block for biodegradable polymers [9, 10, 11, 12]. Conventionally, glycolic acid can either be made by oxidation of ethylene glycol or through reduction of oxalic acid, either by traditional or electrochemical pathways (Scheme 1) [13, 14].

Schuler et al. reported a conventional pathway of thermo‐catalytic reduction to glycolic acid, which requires the use of pressurized hydrogen and transition metal catalysts [15, 16]. Alternatively, Hai et al. and Wang et al. reported a method for the selective reduction of dimethyl oxalate to methyl glycolate using Cu‐Ag/SiO_2_ nanoparticles [17, 18]. Guo et al. published their method using a similar metal catalyst for this reduction [19]. Pickett et al. described a method for the electrochemical reduction of an aqueous oxalic acid solution. However, their method is based on toxic heavy metal electrodes such as mercury or lead. In addition, the formation of glyoxylic acid lacks selectivity [20]. Modern approaches for electrochemical reduction are reported by Abramo et al. and Hao et al., who used TiO_2_ or TiO_2_‐Al^3+^ nanotubes in an electrochemical setup to reduce oxalic acid under mild conditions to glycolic acid [21, 22, 23, 24, 25, 26, 27]. On the other hand, the electrochemical reduction of alkyl oxalates is underexplored. In this context, electrochemistry offers an elegant solution circumventing the use of stoichiometric amounts of reducing agents or transition metal catalysts, high temperatures and high pressures of hydrogen—tremendously ameliorating safety risks and increasing sustainability [28, 29, 30, 31, 32]. The use of electricity in this method from preferred renewable energy sources as a universal and inexpensive reducing agent prevents the formation of reagent waste [33, 34, 35]. In this report (Scheme 1D), we developed a simple and scalable galvanostatic method for the electrochemical reduction of oxalic esters. Isostatic graphite was used as an inexpensive, nonhazardous and readily available cathode material. As a cosolvent and proton source, acetic acid has to solubilize the substrates and provide the acidity required for the chemical transformation [36]. Besides adding tetrabutylammonium acetate as the supporting electrolyte, no other additives are needed. In addition, we were able to show that this process can be adapted for the simple introduction of deuterium into oxygen containing synthesis precursors. Common synthetic strategies of reductive deuteration require pressurized deuterium gas and or a transition metal catalyst (e.g., palladium or ruthenium) or the use of metal deuterides such as NaBD_4_ or LiAlD_4_ [13, 37]. In contrast to that, electrochemistry offers the ability to achieve selective deuteration under mild conditions based on the principles of green and sustainable chemistry [38].

## 2 Results and Discussion

### 2.1 Optimization in Batch‐Type Cells

**SCHEME 2:**
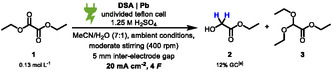
Initial experimental results of the electrochemical reduction of diethyl oxalate.

The electrochemical reduction of oxalic acid to glyoxylic acid in aqueous sulfuric acid is well established in literature [20]. We were interested to see whether these conditions could be applied to the corresponding esters. Based on this knowledge, initial experiments using a simple undivided batch cell with the conditions depicted in Scheme 2 (dimensionally stable anodes (DSA) and lead cathode) were carried out [39].

While a yield of 12% of **2** was observed, residual starting material and an acetal side product **3** were detected too. Due to acidic ester hydrolysis and by‐product formation, sulfuric acid seems not to be the suitable acid for this reaction. Based on knowledge from previous work, screening reactions using weaker acids such as formic or acetic acid were carried out [40]. The conditions (Table 1) were applied to the test reaction for optimization. Information on yield determination, as well as used batch‐type cells, can be found in the supporting information (Figure S1 and GP1).

**TABLE 1:** Optimization of the test reaction.

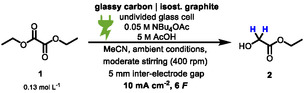			
Entry	Deviation from optimized conditions depicted above	**GC‐** **yield, %** a	Conversion, %
1	None	**97**	**>95**
2	Glassy carbon, nickel (foam), 20 mA cm^–2^, 4 *F*	**0 (95** b **)**	**0**
3	Glassy carbon, lead, 20 mA cm^–2^, 4 *F*	**81**	**>95**
4	Glassy carbon, glassy carbon, 20 mA cm^–2^, 4 *F*	**31 (20** b **)**	**80**
5	Platinum, isost. graphite, 20 mA cm^–2^, 4 *F*	**5 (90** b **)**	**10**
6	20 mA cm^–2^, 4 *F*	**78**	**90**
7	20 mA cm^–2^, 6 *F*	**91**	** >95**
8	5 M HCOOH, 0.1 M NaHCOO	**79**	** >95**
9	5 M HCOOH, 0.1 M NEt_4_BF_4_	**25 (60** b **)**	**35**
10	5 M AcOH, 0.1 M NH_4_OAc	**72**	** >95**
11	MeCN/H_2_O (v:*v* = 1:1)	**20 (25** b **)**	**75**
12	5 mmol, *c*(**1**) = 0.63 mol L^–1^	**93**	** >95**
13	No electricity	**0 (99** b **)**	**0**

a GC quantification against internal standard mesitylene.

b Residual starting material determined by GC.

The analysis of the reactions was done by gas chromatography (GC) to obtain the yield by using mesitylene as an internal standard. The optimized conditions (entry 1) gave a yield of 97% using acetonitrile (MeCN) as solvent and a tetraalkylammonium salt to minimize hydrogen evolution as the main reaction due to accumulation of cations on the surface of the cathode [41, 42, 43, 44, 45, 46]. In addition to acetonitrile, alternative solvents were evaluated; however, they proved unsuitable as they either failed to produce the target product (methanol) or did not provide adequate conductivity (e.g., propylene carbonate or dioxane). Using nickel foam (entry 2), no product was observed and 95% of the starting material was obtained [47]. The only other cathode material which was suitable for this reaction seems to be lead (entry 3). While this electrode material gave a higher yield compared to isostatic graphite, it is toxic, which limits the applicability of the reaction. As such, isostatic graphite was used as the cathode material for further experiments. Using glassy carbon as the cathode material (entry 4), the yield decreased, which can be explained by the different structure of the materials compared to isostatic graphite [48]. Regarding anode materials, platinum (entry 5), which is recognized for its effective anodic oxidation of acetic acid, resulted in a negligible yield yet demonstrated a high recovery rate [49]. Further experiments were carried out using glassy carbon as the anode material due to its good stability and durability [48, 50]. To determine the amount of applied charge required for a high yield, a kinetic study was carried out (supporting information, Figure S9). The results indicate that the required amount of applied charge reaches its maximum around 6 *F* (entry 7) with a yield of 91%. Higher amounts of applied charge did not increase the yield. The applied current density of 10 mA cm^–2^ (entry 1) gave the highest yield with 97%. Using a lower current density, the yield decreased (80%) and the amount of remaining starting material increased (7%). Formic acid and its sodium salt (entry 8) can replace acetic acid in this electrolysis with similar yields. An increase in current density resulted in lower yield, as it caused the formation of potential by‐products. Using tetraethylammonium tetrafluoroborate (entry 9) in combination with formic acid, a significantly lower yield (25%) was observed. With deuterium incorporation in mind, acetic acid was chosen over formic acid for further optimizations due to its lower cost. Using a lower concentration of acetic acid had no significant influence on the yield. When ammonium acetate was used instead of tetrabutylammonium acetate (entry 10), the yield decreased to 72%. This can be explained by the anticorroding effect of the alkylammonium salt on the cathode and the higher overpotential for competition against hydrogen evolution [44]. If a mixture of water and acetonitrile (ratio 1:1, entry 11) was used instead of pure MeCN, only 20% yield was found, with only 25% of the determined residual starting material. Finally, different concentrations of the starting material were investigated. A higher concentration of the starting material (entry 12) did not affect the yield. When considered alongside entry 20, the data indicate that acetic acid concentration serves as the limiting factor in the reaction. In the absence of applied electricity (entry 13), no conversion of the starting material was observed. Based on the optimized conditions from the screening (entry 1), the Faradaic efficiency is calculated at 65%. This value is lower than the theoretical value, which can be explained by possible parasitic side reactions, most likely hydrogen evolution at the cathode. However, the reaction was optimized for yield, not for Faradaic efficiency. All other Faradaic efficiency calculations can be found in the screening tables in the supplementary information.

**SCHEME 3:**
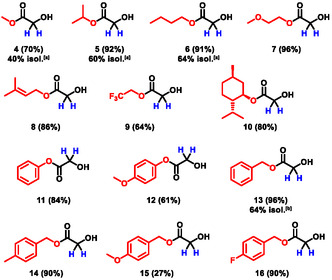
Screening of various oxalic esters resulting in the formation of the corresponding glycolic ester. The optimized electrolysis conditions in batch‐type cells were applied as standard reaction conditions. The yield in brackets was determined by ^1^H NMR spectroscopy using 1,3,5‐trimethoxybenzene as internal standard. ^[a]^Isolation was achieved by direct distillation of the crude reaction mixture from a 10 mmol scale. ^[b]^Isolation was achieved by column chromatography on a 10 mmol scale.

To examine whether different esters have an influence on the yield, screening of various oxalic esters under the optimized conditions was carried out and analyzed using quantitative ^1^H NMR with 1,3,5‐trimethoxybenzene as an internal standard. The calculation of the yield is described in the supporting information. As indicated in Scheme 3, most of the esters are suitable for the reaction and result in high yields. However, there are a few examples which are not suitable for the reaction without adapting the conditions due to solubility issues (**12** and **15**). Highly reactive esters such as fluoro compounds (**9**) gave a significantly lower yield. Regarding the aspect of a simple workup and isolation of the product the aliphatic esters (**2**, **4**–**6**) were preferred since they could be distilled from the crude reaction mixture. A comparison of the aliphatic esters indicates that, apart from the methyl ester, the yields are similar. Isolation was done by simple distillation of the crude reaction mixture.

### 2.2 Scale‐Up in Batch‐Type Cells

Scale‐up experiments were carried out in batch‐type glass cells using diethyl oxalate **1** and the same reaction parameters as the optimized conditions established in the screening (Table 2, entry 12). The isolation of the product was achieved by distillation of the crude reaction mixture in which the solvent (MeCN/acetic acid) could be regained. No GC yields were determined here. It was assumed that these reactions would proceed analogously to the screening reactions and therefore, only the conversion of the starting material was monitored by GC.

**TABLE 2:** Scale‐up experiments in different‐sized batch‐type cells. Further details on the cell design, sizes of the used electrodes and the general procedure can be found in the supporting information (Figure S2, GP1).

Entry	Cell‐volume, mL	**n**_(**starting‐material)**_, **mmol**	**Yield, %** a
1	10	6	**55**
2	30	18	**68**
3	60	36	**75**
4	200	120	**80**

a isolated yield after distillation of the crude reaction mixture.

Comparison with GC quantitative yields showed that isolating small product amounts is challenging, resulting in significant product loss. However, scaling up to larger quantities, the loss of product during distillation lowers and more product can be isolated, which further highlights its robust scalability.

### 2.3 Optimization in Flow Electrolysis

The optimized conditions for the batch‐type electrolysis were transferred to a flow electrochemistry setup [51]. The following conditions (Table 3) were applied as a deviation from the optimized conditions.

**TABLE 3:** Optimization of the reduction of 1 in flow electrolysis.

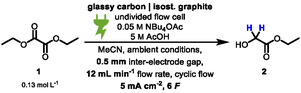		
Entry	Deviation from optimized conditions depicted above	**GC yield, %** a
1	None	**95**
2	10 mA cm^–2^	**84**
3	15 mA cm^–2^	**76**
4	20 mA cm^–2^	**72**
5	25 mA cm^–2^	**68**
6	1.0 mm electrode distance	**85**
7	1.5 mm electrode distance	**79**
8	2.0 mm electrode distance	**78**
9	Flow rate 0.05 mL min^−1^ (single pass)	**0 (95** b **)**
10	Flow rate 5.0 mL min^−1^	**84**
11	Flow rate 10.0 mL min^−1^	**91**
12	Flow rate 15.0 mL min^−1^	**93**
13	Flow rate 20.0 mL min^−1^	**92**
14	No electricity	**0 (99** b **)**
15	Isolation, 5 mmol scale	**95 (55** c **)**

a GC quantification against internal standard mesitylene.

b Recovery of starting material determined by GC.

c Isolated yield after distillation.

Under optimized flow conditions (entry 1), a GC‐determined yield of 95% was achieved. As electrode materials were screened in batch cells, no further tests were conducted in flow. Instead, the focus was set on the parameters specific to flow electrolysis. First, different current densities were applied to the system (entry 2–5). It turned out that the yield decreases significantly with higher current densities. Subsequently, the electrode distance (entry 6–8) was investigated at a constant flow rate of 15 mL min^−1^ by using different sized Teflon spacers. A larger interelectrode gap resulted in longer residence times in the cell, which corresponded to a longer contact time between the electrolyte and the electrode. In the next step, the flow rate was varied (entry 9–13). Using single pass flow (entry 9), no product was observed and 95% of the starting material was recovered. Higher flow rates (entry 12) gave 93% of yield. The product (entry15) was isolated from 5 mmol of starting material by direct distillation, yielding 55%. In a control reaction without electricity (entry 14) no conversion of the starting material was observed.

### 2.4 Reductive Deuteration

For electrochemical reductive deuteration, all proton sources were examined and exchanged to deuterated ones (Table 4). The reaction conditions used for the deuteration reactions are described in the supporting information (GP4). The deuterium incorporation was determined by LCMS [52]. A blindfold experiment (entry 1) demonstrates that MeCN does not participate in proton transfer. When using acetic acid‐*d*_4_ in MeCN (entry 2), 93% deuterium incorporation is observed, with residual water likely accounting for the relatively high 1D percentage of 13%. Therefore, the supporting electrolyte was predried in vacuum at 60°C from MeCN‐*d*_3_ and D_2_O for further experiments. The combination of MeCN‐d_3_ and acetic acid‐*d*_4_ (entry 3) gave an overall incorporation of 96% (8% 1D), same applies to the use of acetic acid‐*d*_1_ (entry 4), which results in 95% overall incorporation. The degree of incorporation determined by ^2^H NMR of this sample was about 96% based on the remaining ^1^H signal. Consequently, acetic acid *d*_4_ is not superior compared to acetic acid *d*_1_. Conditioning the glass cell by overnight treatment with D_2_O/D_2_SO_4_ to remove potential adsorbed protons on the glass wall (entry 5) gave a minor improvement with a total incorporation of 97%. Another minor improvement could be achieved by using ammonium acetate‐*d*_7_ (entry 6). Referring to Table 1 entry 10, the use of ammonium acetate goes along with a loss in yield of around 20%. If the deuterium incorporation does not exceed >98% by using tetrabutylammonium acetate, the use of ammonium acetate‐*d*_7_ is a valid alternative. An overall incorporation of 97% using dry MeCN (entry 7) indicates that the use of MeCN‐*d*_3_ is not necessary. Fresh distillation of diethyl oxalate (entry 8) did not improve the deuterium incorporation.

**TABLE 4:** Evaluation of the deuterium incorporation under consideration of various influences. More details about the experimental setup and experimental data can be found in the Supporting Information (GP4).

Entry	Solvent	Acid	**Supporting** **electrolyte**	2D, %	1D, %	0D, %	Overall D, %
1	MeCN‐*d*_3_	AcOH	NBu_4_OAc	0	0	>99	0
2	MeCN	AcOD_4_	NBu_4_OAc	86	13	<1	93
3	MeCN‐*d*_3_	AcOD_4_	NBu_4_OAc	92	8	<0.5	96
4	MeCN‐*d*_3_	AcOD_1_	NBu_4_OAc	91	8	<1	95
5^a^	MeCN‐*d*_3_	AcOD_1_	NBu_4_OAc	94	6	<0.5	97
6^a^	MeCN‐*d*_3_	AcOD_1_	ND_4_OAc‐*d*_3_	96	4	<0.5	98
7b,, c	MeCN	AcOD_1_	NBu_4_OAc	94	5	<1	97
8b,, c,, d	MeCN	AcOD_1_	NBu_4_OAc	93	7	<1	97

a Conditioning of the glass cell by D_2_O/D_2_SO_4_ treatment overnight to remove adsorbed protons on the glass wall and exchange to deuterons.

b MeCN extra dry with septum.

c Conditioning of the glass cell by TMSCl treatment overnight to remove adsorbed protons on the glass wall and exchange to deuterons.

d Freshly distilled diethyl oxalate.

Finally, the deuteration was transferred to the flow setup using the data according to entry 7. To do a proper isolation, the scale was set to 20 mmol of starting material. Afterward, a distillation of the crude mixture was carried out. A yield of 75% was obtained with an overall deuterium incorporation of 97.5% (95% 2D, 5% 1D according to ^1^H NMR and LCMS).

### 2.5 Reaction Mechanism

**SCHEME 4:**
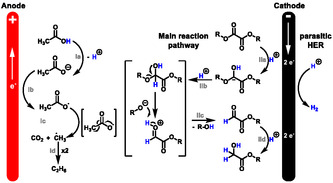
Plausible reaction mechanism for the electrochemical reduction of an oxalic acid ester.

The postulated reaction mechanism is depicted in Scheme 4. The anodic part of the reaction refers to acetic acid oxidation, more precisely, the oxidation of the acetate (**Ia**) liberating H^+^ needed for the cathodic reduction of the ester [49, 53, 54, 55]. NR_4_OAc seems to act sufficiently as a base and therefore, no electrochemical dehydration reaction of the acetate nor the acetic acid was observed [56, 57, 58]. The reduction of the oxalate occurs at the cathode. In the first two electron transfer followed by the cleavage (**IIa**–**IIc**), the ester gets reduced to its corresponding aldehyde. Followed by another two‐electron transfer (**IId**), the aldehyde further reduces to its corresponding alcohol [59]. No further reduction to ethylene glycol was observed. The Faradaic efficiency was calculated at 65%, indicating that a parasitic side reaction, most likely hydrogen evolution in this case, consumes parts of the amount of applied charge. Further experiments underlining those statements can be found in the supporting information.

## 3 Conclusion

A very simple to conduct method for the electrochemical reduction of oxalates to glycolates was established. The reaction relies on nontoxic, inexpensive, and readily available electrode materials and reagents. The method was established in the simplest undivided batch‐type glass cells and could be shown to be both scalable and transferable to a flow electrolysis setup. By distillation of the crude reaction mixture, the product can easily be obtained, which makes this method viable for technical applications. A mechanism which subsequently facilitate the cathodic reduction of the ester using H^+^/D^+^ provided by the acetic acid has been proposed. In addition, this method can be used to selectively obtain deuterated glycolates.

## Supporting Information

Additional supporting information can be found online in the Supporting Information section. **Supporting Scheme S1**: Optimized conditions of the electrochemical conversion of diethyl oxalate into ethyl 2‐hydroxyacetate in batch electrolysis. **Supporting Scheme S2**: Optimized conditions for the screening of different oxalic esters in batch type glass cells. **Supporting Scheme S3**: Optimized conditions of the electrochemical conversion of diethyl oxalate into ethyl 2‐hydroxyacetate in flow electrolysis. **Supporting Scheme S4**: Reaction conditions applied for the investigation of the deuterium incorporation. **Supporting Scheme S5**: Overview of the commercially bought and synthesized oxalic esters. **Supporting Fig. S1**: The electrolysis setup: A glass‐cell (1) with a small stirrer inside was used for the batch screening experiments (10 mL total volume, 8 mL experimental volume). A screwable Teflon^TM^ (4) cap with titanium electrode holders was used with a glassy carbon anode (2 left) and an isostatic graphite cathode (3 right). On the right side the disassembled TeflonTM screwable electrode cap is displayed. **Supporting Fig. S2**: The scale electrolysis setup for reference purpose: A 50 mL batch‐type glass cell (1) equipped with a magnetic stir bar and the two electrodes. On the right (2) the disassembled 50 mL and 100 mL cell. At the bottom (3), the sizes of the used glassy carbon and isostatic graphite electrodes are displayed. **Supporting Fig. S3**: The flow‐electrolysis setup: on the left side (1): Fully disassembled cell with two Teflon^TM^ half‐cells equipped with 2.0 × 6.0 × 0.3 cm electrodes, Glassy carbon anode and an isostatic. graphite cathode and a 0.5 mm Teflon^TM^ spacer and the screws. On the right side (2): Fully assembled flow cell. At the bottom (3), the sizes of the used isostatic graphite half‐cell are displayed. **Supporting Fig. S4**: GC Chromatogram of the three different compounds with the method used for the calibration. **Supporting Fig. S5**: Calibration curve of the starting material, diethyl oxalate. **Supporting Fig. S6**: Calibration curve of the desired product, ethyl 2‐hydroxyacetate. **Supporting Fig. S7**: Visualization of the determination of the yield by quantitative ^1^H NMR using 1,3,5‐Trimethoxybenzene as internal standard. **Supporting Fig. S8**: Combined GC‐chromatograms of each sample of the kinetic flow electrolysis experiment. **Supporting Fig. S9**: Plotting of the calculated yield and the conversion of the starting material according to the GC‐calibration depending on the amount of applied charge. **Supporting Fig. S10**: Influence of different sandpaper grain sizes preparing the surface of the graphite electrode on the flow electrolysis of diethyl oxalate into ethyl 2‐hydroxyacetate under exact same electrolysis conditions (GC|isost. graphite, 0.5 mm inter‐electrode distance, 6 F, 5 mA·cm^‐2^, 12 mL min^‐1^ flow rate, 5 M AcOH / 0.05 M NBu_4_OAc, MeCN). **Supporting Fig. S11**: ^1^H NMR Spectrum of entry 2 indicating partial ester hydrolysis but no HIE. **Supporting Fig. S12**: GC chromatogram of the electrolysis mixture after the reaction (red) against the reference of *n*‐decane (black). **Supporting Fig. S13**: ^13^C NMR spectrum of the crude reaction mixture. **Supporting Fig. S14**: GC chromatogram of the electrolysis after different amounts of applied charge. The formation of the aldehyde intermediate can be seen on the left‐hand side of the chromatogram. **Supporting Fig. S15**: ^1^H NMR after the electrolysis of dibenzyl oxalate. The formation of benzyl alcohol can be observed. **Supporting Table S1**: GC temperature program of the used method with an injector temperature of 250°C and a detection temperature of 315°C with a total time of 22 min. **Supporting Table S2**: GC calibration data – weigh in of the used compounds. **Supporting Table S3**: GC calibration data – total area of the internal standard. **Supporting Table S4**: GC calibration data – total area of the starting material. **Supporting Table S5**: GC calibration data – total area of the desired product. **Supporting Table S6**: GC calibration data for the plot of the calibration curve. **Supporting Table S7**: GC calibration data for the plot of the calibration curve. **Supporting Table S8**: GC calibration curve validation measurement. **Supporting Table S9**: Optimized conditions, no electricity control experiment and reference experiment with ^1^H NMR quantification. **Supporting Table S10**: Screening of the different cathode materials. **Supporting Table S11**: Screening of different anode materials. **Supporting Table S12**: Screening of the amount of applied charge. **Supporting Table S13**: Screening of the applied current density. **Supporting Table S14**: Screening of the different electrolytic systems. **Supporting Table S15**: Screening of the different starting material concentrations. **Supporting Table S16**: Screening of different oxalic esters under optimized conditions (1 mmol [0.13 M], GC | isost. graphite, 8 mL volume, 6.0 *F*, 10 mA·cm^−2^, MeCN, 5 M AcOH / 0.05 M NBu_4_OAc). **Supporting Table S17**: Screening of different inter‐electrode gaps. **Supporting Table S18**: Screening of different flow rates. **Supporting Table S19**: Screening of different current densities. **Supporting Table S20**: Conditions for the kinetic flow electrolysis experiment. **Supporting Table S21**: Optimization of the deuterium incorporation in the final product. **Supporting Table S22**: Control reactions for the HIE. **Supporting Table S23**: Data evaluation of the screening of different oxalic esters in batch type cells.

## Funding

This work was supported by Bundesministerium für Bildung, Wissenschaft, Forschung und Technologie Clusters4future ETOS–Electrifying Technical Organic Synthesis (03ZU1205HC), Max–Planck–Gesellschaft (Open Acess DEAL Agreement), and Deutsche Forschungsgemeinschaft (EXC‐2033‐390677874 – RESOLV).

## Conflicts of Interest

Dr. Volker Derdau is employee of Sanofi and holds shares and stocks of the company.

## Supporting information

Supplementary Material

## Data Availability

The data that support the findings of this study are available in the supplementary material of this article.
