# Supplementary material for: Simple and Scalable Electrochemical Reduction of Alkyl Oxalates Using Carbon‐Based Electrodes
Source: ChemSusChem. 2026 Mar 22;19(6):e202502557. doi: 10.1002/cssc.202502557 (PMC13006163; doi:10.1002/cssc.202502557)
Supplement: Supplementary file 1 — Supplementary Material [file CSSC-19-e202502557-s001.pdf]

Electronic Supporting Information (ESI)

Simple and Scalable Electrochemical Reduction of Alkyl Oxalates using Carbon-based Electrode Materials

Sebastian Kissel[a], Dr. Philipp Schnieders[c], Dr. Volker Derdau[d] and Siegfried R. Waldvogel*[a,b]

[a] S. Kissel, Prof. Dr. S. R. Waldvogel
Max-Planck-Institute for Chemical Energy Conversion, Department of Electrosynthesis
Stiftstraße 34-36,45470 Mülheim an der Ruhr (Germany)
E-mail: siegfried.waldvogel@cec.mpg.de

[b] Prof. Dr. S. R. Waldvogel
Karlsruhe Institute of Technology, Institute of Biological and Chemical Systems – Functional Molecular Systems (IBCS-FMS)
Kaiserstraße 12, 76131 Karlsruhe (Germany)

[c] Dr. Philipp Schnieders
Deutero GmbH
Am Ring 29, 56288 Kastellaun (Germany)

[d] Dr. Volker Derdau
Sanofi R&D, Integrated Drug Discovery, Isotope Chemistry
Industriepark Höchst, 65926 Frankfurt am Main (Germany)

Table of Contents

1. **General information** S2

2. **General procedures** S10

   GP1: Optimization of the electrolytic reduction of diethyl oxalate in batch-type cell S10

   GP2: Screening of different oxalic esters under optimized conditions S14

   GP3: Optimization of the diethyl oxalate reduction in flow electrolysis S17

   GP4: Electrolytic reduction of diethyl oxalate in batch cell using deuterated compounds. S20

3. **Mechanistic studies on the reduction of oxalic esters in acetic acid solution** S22

4. **Synthesis of oxalic esters** S26

   GP5: General synthesis of different alkyl and aryl oxalates S26

5. **NMR characterization of isolated products after electrolysis** S32

6. **GC spectra of the starting materials** S34

7. **NMR spectra of the starting materials and the isolated products** S42

General information

All reactions were performed under ambient conditions, if not stated otherwise, and chemicals in analytical grade were used as purchased (BLD-Pharm, Sigma Aldrich, TCI etc.) without further purification. Cyclohexane and ethyl acetate were purchased in technical grade and purified by distillation at reduced pressure prior to use. Milli-Q™ water was obtained using Simplicity™ System (UV) (*Merck KGaA*, Darmstadt, Germany) for chromatography purposes. Anhydrous solvents were obtained from a solvent purification system SPS-5 (*M. Braun Incorporated*, Stratham, USA). Deuterated solvents for NMR analysis and compounds (deuterated acetic acid and ND_4OAc-d_3) for the deuteration reactions were purchased from Deutero GmbH (Kastellaun, Germany).

Chromatography

Thin layer chromatography was performed using silica gel DC Kieselgel 60 F254 on aluminium plates (*Merck KGaA,* Darmstadt, Germany). A UV lamp (λ = 254 nm, NU-4 KL, *Benda*, Wiesloch, Germany). For preperative column chromatography prepacked spherical puriFlash® SI-HP silica columns (*Interchim SAS*, Montluçon Cedex, France) with particle sizes of 15, 25 or 30 µm were used using a puriFlash® XS 520 Plus system (*Interchim SAS*, Montluçon Cedex, France).

High Performance Liquid Chromatography (HPLC)

Analysis of crude reaction mixtures and purified products was performed using a modular system LC-20A *Prominence* (*Shimadzu Deutschland GmbH*, Duisburg, Germany), UV/VIS-detector SPD-20A/AV (*Shimadzu Deutschland GmbH*, Duisburg, Germany), and LCMS-2020 Single Quadrupole (*Shimadzu Deutschland GmbH*, Duisburg, Germany). Analytical separation was performed using an Eurospher II 100-5 C-18A-Trennsäule (*Knauer Wissenschaftliche Geräte GmbH*, Berlin, Germany) column (length of 150 mm, diameter of 4 mm, pore size of 100 Å, particle size 5 µm). As eluents, acetonitrile (MeCN) and water with 5% (*v*/*v*) MeCN and formic acid (1 mL L^{-1}) were used. Given retention times were obtained at λ = 254 nm. To determine the deuterium incorporation a specific matrix was used that allows to use the isotope pattern of the mass to calculate the degree of incorporation.[1]

Nuclear Magnetic Resonance (NMR) Spectroscopy

Nuclear magnetic resonance experiments were performed using a nuclear magnetic resonance spectrometer Avance III HD300 (*Bruker*, Karlsruhe, Germany) ^{1}H NMR (300 MHz), ^{19}F NMR (282 MHz), Avance II 400 (*Bruker*, Karlsruhe, Germany) ^{1}H NMR (400 MHz), ^{13}C NMR (101 MHz), and ^{19}F NMR (376 MHz), Avance III 600 (*Bruker*, Karlsruhe, Germany) ^{1}H NMR (600 MHz), ^{13}C NMR (151 MHz) (5 mm BBFO-SmartProbe with z gradient and ATM, SampleXPress 60 sample changer, Analytische Messtechnik, Karlsruhe, Germany) or Bruker Ascend Evo 400 NMR spectrometer with a Bruker Prodigy probe (Bruker BioSpin GmbH, Rheinstetten, Germany). The spectra were recorded using deuterated solvents at a temperature of 25 °C. To normalize the obtained spectra, reference was made to the existing solvent signal of non-deuterated fractions according to the data provided by *Fulmer et al.*[1]: $CDCl_3$ (^{1}H NMR: δ = 7.26 ppm, ^{13}C NMR: δ = 77.2 ppm), dichloromethane-d_2 (^{1}H NMR: δ = 5.32 ppm, ^{13}C NMR: δ =53.8 ppm), methanol-d_4 (^{1}H NMR δ = 3.31 ppm, ^{13}C NMR: δ = 49.0 ppm) and water-d_2 (^{1}H NMR δ = 4.79 ppm) acetonitrile-d_3 (^{1}H NMR δ = 1.94 ppm, ^{13}C NMR: δ = 118.3 ppm). Besides ^{1}H, ^{13}C and ^{19}F NMR experiments, the 2D techniques ^{1}H,^{1}H-COSY, ^{1}H,^{13}C-HSQC, ^{1}H,^{13}C-HMBC were used assisting to assign the signals. The following abbreviations were used to describe the signals: s (singlet), d (doublet), t (triplet), dd (doublet of doublets), td (triplet of doublets), m (multiplett), q (quartet), hep (heptet). The spectra obtained were evaluated with MestReNova 14.2.0-26256 (*Mestrelab Research S.L.*, Spain).

The NMR yield is calculated as follows, where I denotes the integral of the ^{1}H NMR signal, and N is the number of protons corresponding to that signal:

$$\%\text{yield}_{\text{NMR}} = \frac{n_{\text{product}} \cdot 100}{n_{\text{starting material}}} = \frac{n_{\text{std}} \cdot \frac{\left(\frac{I_{\text{product}}}{N_{\text{product}}}\right)}{\left(\frac{I_{\text{std}}}{N_{\text{std}}}\right)} \cdot 100}{n_{\text{starting material}}}$$

1,3,5-Trimethoxybenzene was used as internal standard, $N_{\text{std}} = 3$, the 3 aromatic protons are used for integration.

Electrochemical Set-Up

Electrochemical reactions were carried out using a multichannel galvanostat HMP4040 (*Rohde & Schwarz*, München, Germany) with a controllable DC output of 0-32 V and 0–10 A and a maximum power of 160 W per channel was used as power source. The different cells used for screening or batch reactions are described below.

Screening Reactions

First screening reactions were carried out using Teflon™ cells with a volume of 5 mL. It turned out that the Teflon™ cells used were not suitable, so screening glass cells (*IKA™ Werke GmbH & Co. KG*, Staufen, Germany) with a volume of 10 mL were used for all further screening experiments. The lid of the cell equipped with two rubber septa and two electrode holders was developed in the workshop of the University of Mainz in collaboration with the WALDVOGEL lab. Electrodes with a size of 0.8 x 5.1 x 0.2 cm were used in the cells.

For the flow electrolysis the Eletrcasyn Flow System (*IKA™ Werke GmbH & Co. KG*, Staufen, Germany) was used with an electrode size of 2.0 cm x 6.0 cm, 0.3 cm. To provide the flow rate, a one channel peristaltic pump (Masterflex ISMATEC®) with different PharMed® BPT tubing (e.g. triple purple ID: 2.06mm, ISMATEC®**)** was used.

For the scale-up experiments larger batch type glass cells depicted in Figure S2 were used.

Electrode Material	Remarks	Supplier
Boron-doped diamond (DIACHEM®)	15 µm boron-doped diamond layer on silicon	*CONDIAS GmbH*, Itzehoe, Germany
Glassy carbon (GC)	-	*HTW*, Thierhaupten, Germany
Isostatic graphite Sigrafine™	-	*SGL Carbon*, Bonn, Germany
Lead	-	*Globus Fachmärkte GmbH & Co. KG*, Völklingen, Germany (home depot)
Platinum	>99%	*OEGUSSA*, Vienna, Austria
Nickel (foam, Ni-4753)	>99.9%	Recemat BV, Dodewaard, The Netherlands
Reticulated vitreous carbon (RVC)	-	ERG Aerospace Corporation, Oakland, Untied States of America
Leaded bronze	CuSn7Pb15	*Metallwerk Langenau GmbH*, Germany

The surface of the graphite electrodes was polished using sand papers of different grain sizes (240, 600, 1000, 1200, *Bosch*, Stuttgart, Germany) bought from the local hardware store.

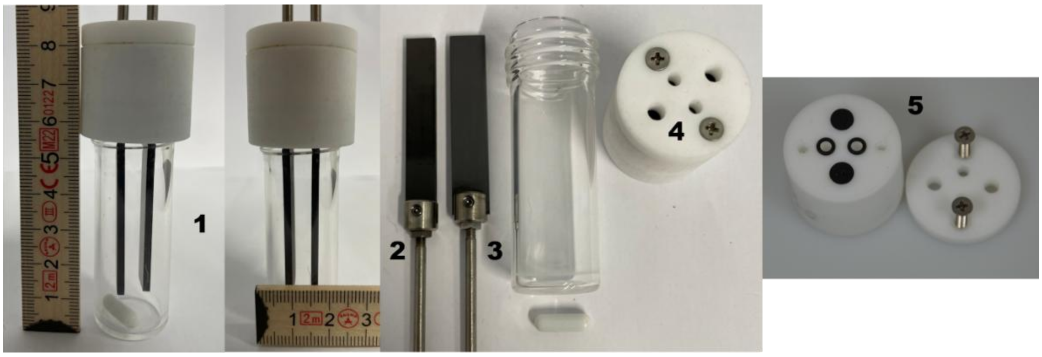

Figure S1: The electrolysis setup: A glass-cell (1) with a small stirrer inside was used for the batch screening experiments (10 mL total volume, 8 mL experimental volume). A screwable Teflon^{TM} (4) cap with titanium electrode holders was used with a glassy carbon anode (2 left) and an isostatic graphite cathode (3 right). On the right side the disassembled Teflon^{TM} screwable electrode cap is displayed (5).

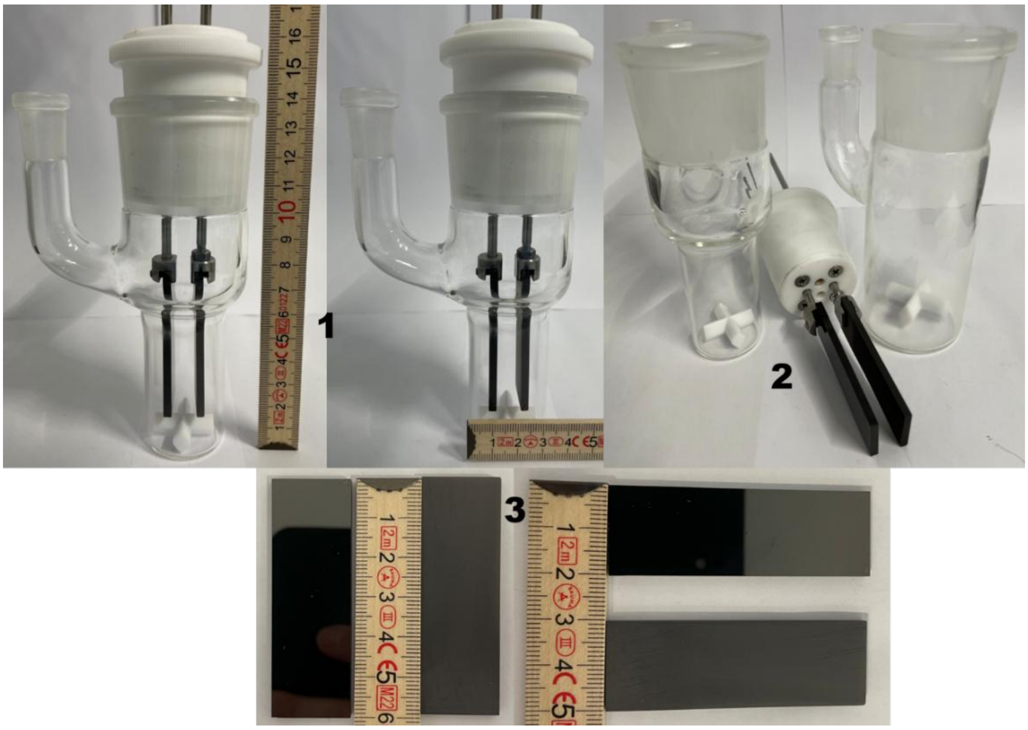

Figure S2: The scale electrolysis setup for reference purpose: A 50 mL batch-type glass cell (1) equipped with a magnetic stir bar and the two electrodes. On the right (2) the disassembled 50 mL and 100 mL cell. At the bottom (3), the sizes of the used glassy carbon and isostatic graphite electrodes are displayed.

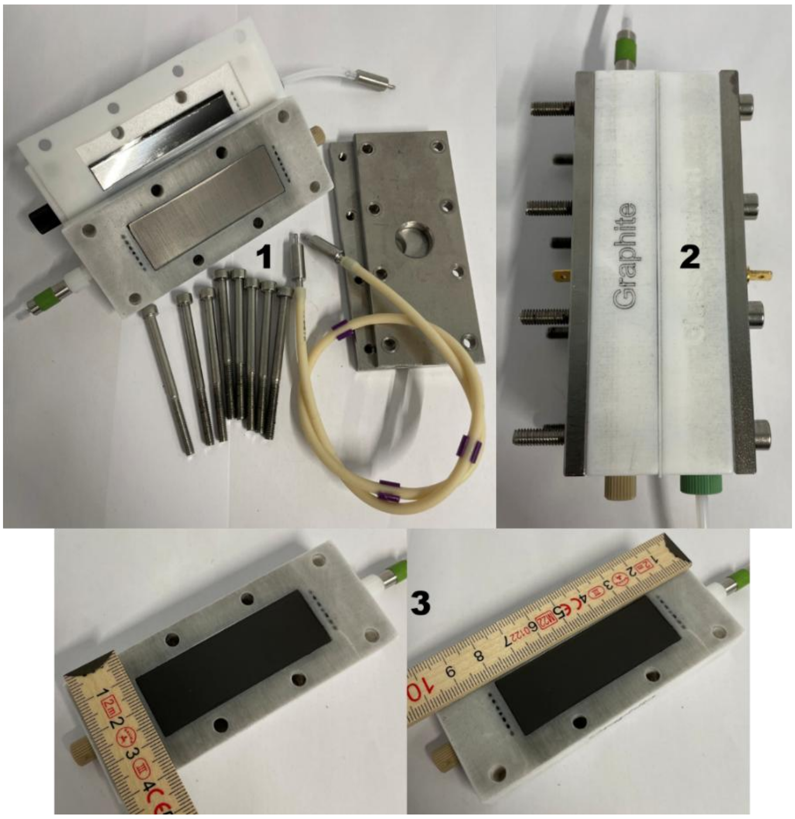

Figure S3: The flow-electrolysis setup: on the left side (1): Fully disassembled cell with two Teflon™ half-cells equipped with 2.0 x 6.0 x 0.3 cm electrodes, Glassy carbon anode and an isostatic. graphite cathode and a 0.5 mm Teflon™ spacer and the screws. On the right side (2): Fully assembled flow cell. At the bottom (3), the sizes of the used isostatic graphite half-cell are displayed.

Gas Chromatography with Mass Spectrometry (GCMS)

Analysis of crude reaction mixtures and purified products were performed using a GCMS QP2010SE (*Shimadzu*, Kyoto, Japan) equipped with an electron ionization (EI) source and a quadrupole mass analyzer. A quartz capillary column HI-5MS (*Avantor VWR*, Radnor, USA) with the following specification was used: length of 30 m, inner diameter of 0.25 mm and a stationary phase ((5%-phenyl)-dimethylsiloxane) of 0.25 µm thickness. Helium was used as carrier gas with a constant velocity of 30 cm/s. The GC temperature ramp started at 50 °C (holding for 1 min) and heating to 300 °C (holding for 4.71 min) with a temperature ramp of 17.5 °C/min (method: 2_medium, total program time: 20.0 min). Measurements were performed at an injector temperature of 270 °C and a temperature of the EI source of 250 °C.

Gas Chromatography (GC)

Gas chromatography was performed using a GC-2010 (*Shimadzu*, Kyoto, Japan) equipped with a flame ionization detector (FID) and a quartz capillary ZB-5HT *Inferno*™ (*Phenomenex*, Torrance, CA, USA) with following specification: length of 30 m, inner diameter of 0.25 mm and a stationary phase ((5%-phenyl)-dimethylsiloxane) of 0.25 µm thickness. Hydrogen was used as carrier gas with a total flow rate of 55.5 mL min^{-1}. The linear velocity is 40 cm s^{-1}. Nitrogen was used as the makeup gas for the FID detector. All methods have a split ratio of 35. The method used for analysis are shown in Table S1.

Table S1: GC temperature program of the used method with an injector temperature of 250°C and a detection temperature of 315°C with a total time of 22 min.

Temperature [°C]	Heating ramp [°C min^{-1}]	Holding time [min]
40	-	2.2
80	7.1	-
120	6	-
300	40	xx

GC Calibration standard substrate

In order to determine the yields of the screening experiments for the model substrate an external calibration of the FID was performed with three components: **diethyl oxalate, ethyl 2-hydroxyacetate** and **mesitylene**. The calibration was created using percentage steps of the maximum yield of 1 mmol.

To determine the yield of the reaction, the area of the standard was netted against the area of the product and this ratio was used to determine the amount of substance of the product and thus the percentage yield.

All plots were obtained by using Origin 2023 Pro (*OriginLab Corp.*,Northhampton, (MA), USA). The following equation shows the calculation of the amount of substance.

$$n_{product} = n_{IStd} * k * \frac{A_{\text{product}}}{A_{\text{IStd}}}$$

Figure S3 shows the reference spectrum with all three compounds used in the calibration. The following tables are necessary for the preparation of the calibration.

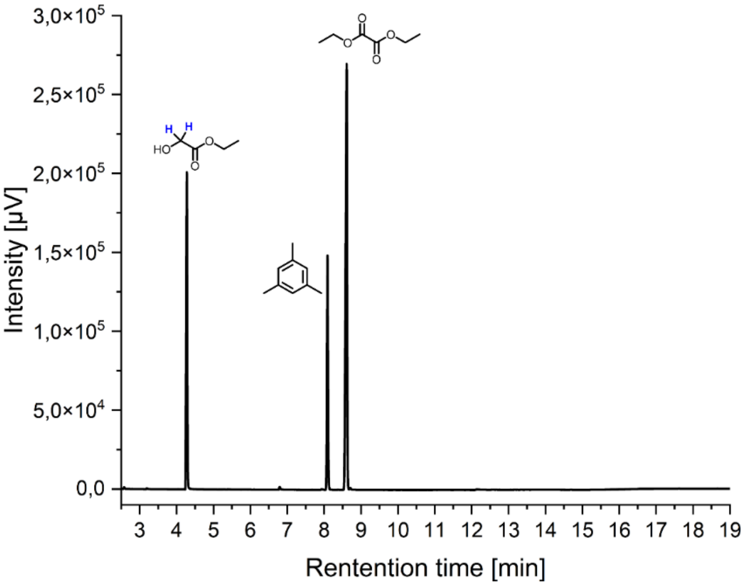

Figure S4: GC Chromatogram of the three different compounds with the method used for the calibration**.**

Table S2: GC calibration data – weigh in of the used compounds.

Entry	$V_{mesitylene}$ [µL]	$n_{mesitylene}$ [µmol]	m_{SM} [mg]	n_{SM} [µmol]	$n_{product}$ [µmol]	$m_{product}$ [mg]
**K0-Blank**	50	362.8	-	-	-	-
**K1-10%**	50	362.8	18.2	124.6	12.8	123.0
**K2-20%**	50	362.8	28.3	193.8	19.9	191.3
**K3-40%**	50	362.8	59.7	408.7	41.1	395.0
**K4-60%**	50	362.8	90.2	617.5	62.0	595.9
**K5-80%**	50	362.8	116.5	797.6	84.5	812.1
**K6-90%**	50	362.8	130.8	895.5	96.5	927.4
**K7-100%**	50	362.8	146.0	999.6	104.0	999.5

Table S3: GC calibration data – total area of the internal standard.

Entry	Run 1	Run 2	Run 3	Average
**K0-Blank**	1434242	-	-	1434242
**K1-10%**	1103900	1095855	1095254	1098336
**K2-20%**	1039954	1032073	1020219	1030749
**K3-40%**	1053691	1031313	1043256	1042854
**K4-60%**	970341	955415	941913	955890
**K5-80%**	730541	721873	719918	724111
**K6-90%**	700929	965348	707163	701147
**K7-100%**	452863	447576	447048	447162

Table S4: GC calibration data – total area of the starting material.

Entry	Run 1	Run 2	Run 3	Average
**K0-Blank**	-	-	-	-
**K1-10%**	152073	150984	150026	151028
**K2-20%**	223542	222065	220159	221922
**K3-40%**	487166	475504	486421	483030
**K4-60%**	683614	671403	666514	673844
**K5-80%**	668484	653008	658480	659991
**K6-90%**	720102	716038	732816	722985
**K7-100%**	552093	516492	508872	515819

Table S5: GC calibration data – total area of the desired product.

Entry	Run 1	Run 2	Run 3	Average
**K0-Blank**	-	-	-	-
**K1-10%**	76516	75899	74851	75755
**K2-20%**	114539	113516	112496	113517
**K3-40%**	245161	239366	244832	243130
**K4-60%**	344480	339046	336000	339842
**K5-80%**	4256968	351061	352803	353611
**K6-90%**	391234	389232	398300	392922
**K7-100%**	272937	269924	265725	269529

Table S6: GC calibration data for the plot of the calibration curve.

Entry	$A_{product}/A_{IStd}$	$n_{product}/n_{IStd}$	A_{SM}/A_{IStd}	n_{SM}/n_{IStd}
**K0-Blank**	0.00	0.00	0.00	0.00
**K1-10%**	0.07	0.34	0.14	0.34
**K2-20%**	0.11	0.53	0.22	0.53
**K3-40%**	0.23	1.09	0.46	1.13
**K4-60%**	0.36	1.64	0.70	1.70
**K5-80%**	0.49	2.24	0.91	2.20
**K6-90%**	0.56	2.56	1.03	2.47
**K7-100%**	0.61	2.76	1.16	2.76

Table S7: GC calibration data for the plot of the calibration curve.

Entry	Gradient (k)	Intersection (y-axis)	Pearson R^2
**Starting material**	**2.38995 (±0.0071)**	0	0.99992
**Product**	**4.56499 (±0.01425)**	0	0.99991

Table S8: GC calibration curve validation measurement.

Entry	$V_{mesitylene}$ [µL]	$n_{mesitylene}$ [µmol]	m_{SM} [mg]	n_{SM} [mmol]	n product [µmol]	m product [mg]	$A_{mesitylene}$	A_{SM}	$A_{product}$	Yield [%]
**Starting material**	50	362.8	145.7	0.997	-	-	335304	386431	-	**99**
**Product**	50	362.8	104.5	1.006	104.5	1.007	335304	-	194006	**96**

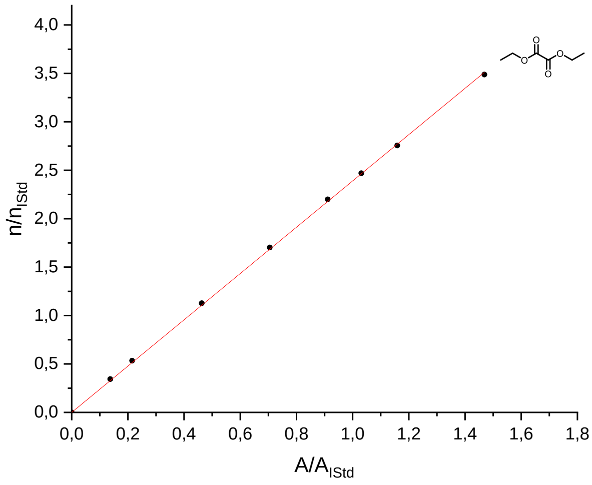

Figure S5: Calibration curve of the starting material, diethyl oxalate.

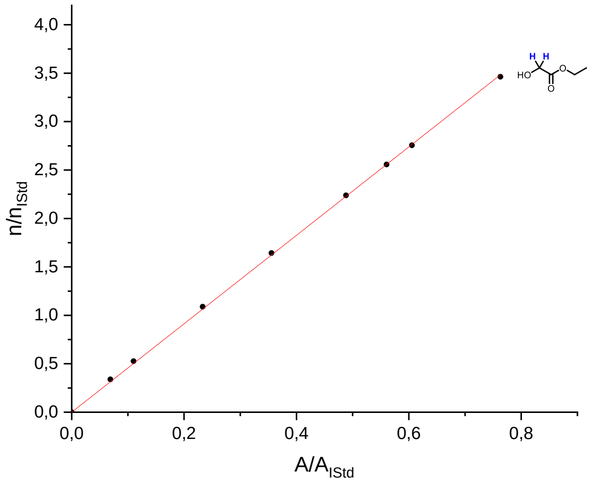

Figure S6: Calibration curve of the desired product, ethyl 2-hydroxyacetate.

1. General procedures

GP1: Optimization of the electrolytic reduction of diethyl oxalate in batch-type cell

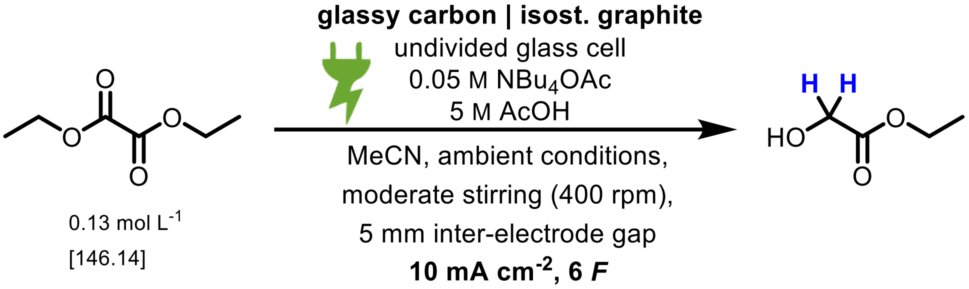

Scheme S1: Optimized conditions of the electrochemical conversion of diethyl oxalate into ethyl 2-hydroxyacetate in batch electrolysis.

The 10 mL glass cell equipped with a magnetic stir bar was charged with diethyl oxalate (1.0 mmol, 146.1 mg). To this, 8 mL of a premade solution of acetic acid (5 M, 58mL) and tetrabutylammonium acetate (0.05 M, 3.14 g) in MeCN (total volume of 200 mL) was added. Upon dissolution, the electrodes were put into the electrolyte and the galvanostatic electrolysis was started (6.0 *F*, 10 mA cm^{-2}, room temperature, under air, GC (anode), isostatic graphite (cathode), both electrodes 0.8 cm x 5.1 cm, 5 mm inter-electrode gap, moderate stirring (400 rpm)). From 6.0 *F,* Q = 581 *C* is calculated ($Q = n \cdot z \cdot F$). The electrolysis time calculated with $Q = I \cdot t$ and $j = \frac{I}{A}$ is 5 h and 23 mins. After electrolysis is completed, 50 µL of mesitylene as internal standard were added to the mixture. A part of the solution was filtered through a small silica column to remove the supporting electrolyte before the sample was analyzed by GC-chromatography. Deviations from the standard conditions marked in **red** (**Scheme 1**) are shown in the following tables (**Tables S9-S15**).

Scale-up experiments were carried out according to the parameters in Scheme 1 using larger batch-type beaker cells according to Figure S2. Isolation of the desired product was achieved by distillation of the crude reaction mixture under vacuum.

Table S9: Optimized conditions, no electricity control experiment and reference experiment with ^{1}H NMR quantification.

Entry	Anode	Cathode	Solvent	Supporting electrolyte	Current density *j* [mA cm^{-2}]	Applied charge *Q* [*F*]	n_{SM} [mmol]	c_{SM} [mmol mL^{-1}]	GC yield [%][a]	FE [%]
**1**	GC	isost. graphite	MeCN	AcOH / NBu_4OAc	10	6.0	1.0	0.13	**97**	**65**
**2**	GC	isost. graphite	MeCN	AcOH / NBu_4OAc	**no**	**no**	1.0	0.13	**- (99[b])**	**-**
**3**	GC	isost. graphite	MeCN	AcOH / NBu_4OAc	10	6.0	1.0	0.13	**95[c]**	**63**

[a] GC-quantification against internal standard mesitylene. [b] Residual starting material. [c] Verification of the GC yield by **^{1}H NMR** using 1,3,5-trimethoxybenzene as internal standard (1.0 mmol, 168.2 mg).

Table S10: Screening of the different cathode materials.

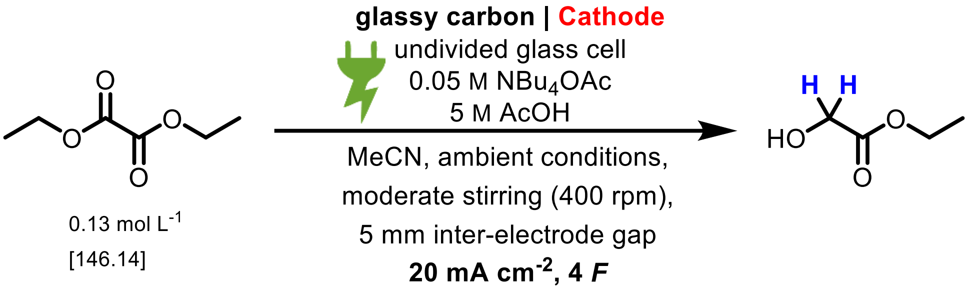

Entry	Anode	Cathode	Solvent	Supporting electrolyte	n_{SM} [mmol]	GC yield [%][a]	FE [%]
1	GC	RVC	MeCN	AcOH / NBu_4OAc	1.0	**47 (35[b])**	**47**
2	GC	nickel (foam)	MeCN	AcOH / NBu_4OAc	1.0	**- (95[b])**	**-**
3	GC	Pb	MeCN	AcOH / NBu_4OAc	1.0	**81**	**81**
4	GC	GC	MeCN	AcOH / NBu_4OAc	1.0	**31 (20[b])**	**31**
5	GC	CuSn7Pb15	MeCN	AcOH / NBu_4OAc	1.0	**- (95[b])**	**-**

[a] GC-quantification against internal standard mesitylene. [b] Residual starting material.

Table S11: Screening of different anode materials.

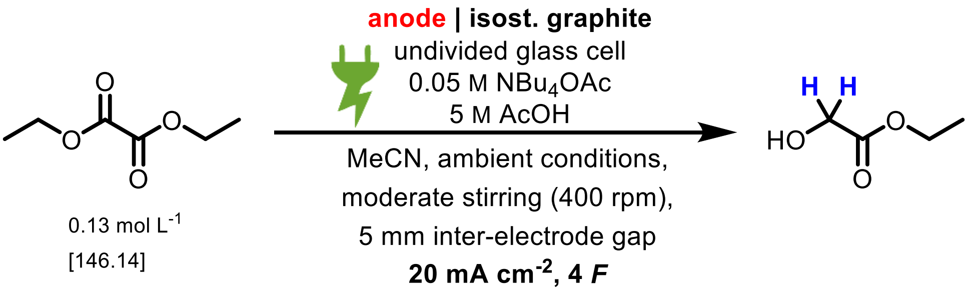

Entry	Anode	Cathode	Solvent	Supporting electrolyte	n_{SM} [mmol]	GC yield [%][a]	FE [%]
1	isost. graphite	isost. graphite	MeCN	AcOH / NBu_4OAc	1.0	**74**	**74**
2	RVC	isost. graphite	MeCN	AcOH / NBu_4OAc	1.0	**74**	**74**
3	platinum	isost. graphite	MeCN	AcOH / NBu_4OAc	1.0	**5 (90[b])**	**5**
4	GC	isost. graphite	MeCN	AcOH / NBu_4OAc	1.0	**78**	**78**

[a] GC-quantification against internal standard mesitylene. [b] Residual starting material.

Table S12: Screening of the amount of applied charge.

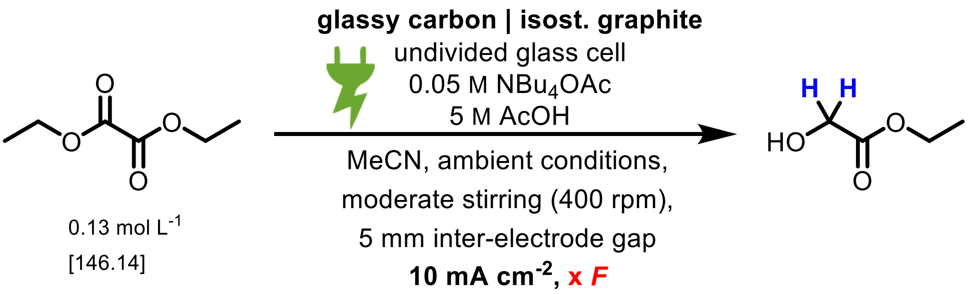

Entry	Anode	Cathode	Solvent	Supporting electrolyte	Current density *j* [mA cm^{-2}]	Applied charge *Q* [*F*]	n_{SM} [mmol]	GC yield [%][a]	FE [%]
**1**	GC	isost. graphite	MeCN	AcOH / NBu_4OAc	20	4.0	1.0	**78**	**78**
**2**	GC	isost. graphite	MeCN	AcOH / NBu_4OAc	20	5.0	1.0	**86**	**69**
**3**	GC	isost. graphite	MeCN	AcOH / NBu_4OAc	20	6.0	1.0	**91**	**60**
**4**	GC	isost. graphite	MeCN	AcOH / NBu_4OAc	20	7.0	1.0	**90**	**51**
**5**	GC	isost. graphite	MeCN	AcOH / NBu_4OAc	20	8.0	1.0	**91**	**46**

[a] GC-quantification against internal standard mesitylene. [b] Residual starting material.

A full plot (seen in **Figure S9**) of the amount of applied charge was done in the flow-cell.

Table S13: Screening of the applied current density.

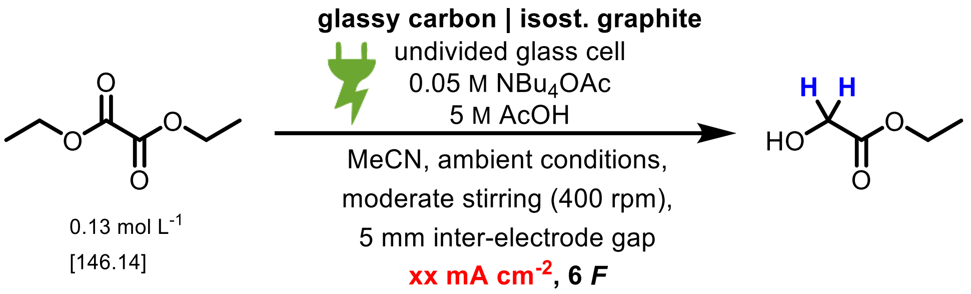

Entry	Anode	Cathode	Solvent	Supporting electrolyte	Current density *j* [mA cm^{-2}]	n_{SM} [mmol]	GC yield [%][a]	FE [%]
**1**	GC	isost. graphite	MeCN	AcOH / NBu_4OAc	5	1.0	**80 (7[b])**	**53**
**2**	GC	isost. graphite	MeCN	AcOH / NBu_4OAc	10	1.0	**97**	**65**
**3**	GC	isost. graphite	MeCN	AcOH / NBu_4OAc	15	1.0	**92**	**61**
**4**	GC	isost. graphite	MeCN	AcOH / NBu_4OAc	25	1.0	**88**	**59**
**5**	GC	isost. graphite	MeCN	AcOH / NBu_4OAc	25	1.0	**88**	**59**

[a] GC-quantification against internal standard mesitylene. [b] Residual starting material.

Table S14: Screening of the different electrolytic systems.

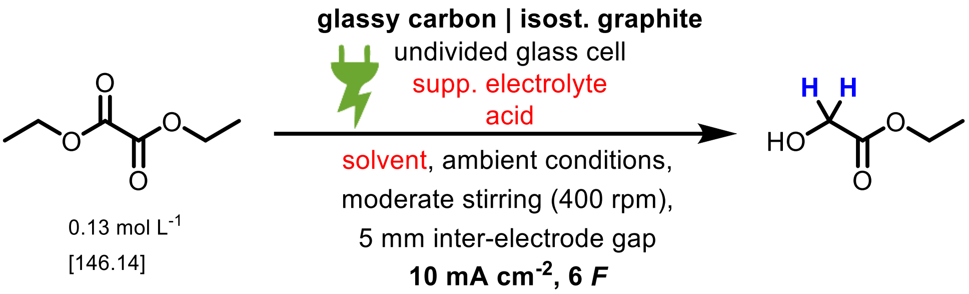

Entry	Anode	Cathode	Solvent	Supporting electrolyte	Current density *j* [mA cm^{-2}]	Applied charge *Q* [*F*]	n_{SM} [mmol]	GC yield [%][a]	FE [%]
**1**	GC	isost. graphite	MeCN	5M HCOOH /0.1 M NaHCOO	10	6.0	1.0	**79**	**53**
**2**	GC	isost. graphite	MeCN	5M HCOOH /0.1 M NH_4HCOO	10	6.0	1.0	**90**	**60**
**3**	GC	isost. graphite	MeCN	5M HCOOH /0.05 M NEt_4BF_4	10	6.0	1.0	**25 (60[b])**	**17**
**4**	GC	isost. graphite	MeCN	2 M AcOH / 0.05 M NBu_4OAc	10	6.0	1.0	**95**	**63**
**5**	GC	isost. graphite	MeCN	5 M AcOH / 0.1 M NH_4OAc	10	6.0	1.0	**72**	**48**
**6**	GC	isost. graphite	MeCN:water 1:1 (v:v)	5M AcOH / 0.1 M NBu_4OAc	10	6.0	1.0	**25 (25[b])**	**17**

[a] GC-quantification against internal standard mesitylene. [b] Residual starting material

Table S15: Screening of the different starting material concentrations.

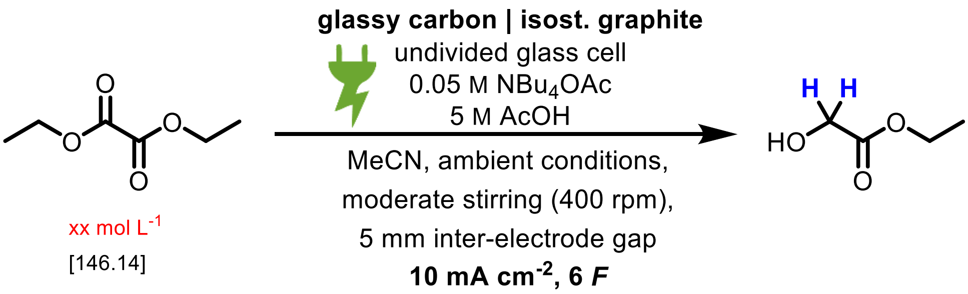

Entry	Anode	Cathode	Solvent	Supporting Electrolyte	n_{SM} [mmol]	c_{SM} [mmol mL^{-1}]	GC yield [%][a]	FE [%]
**1**	GC	isost. graphite	MeCN	AcOH / NBu_4OAc	1.0	0.13	**97**	**65**
**2**	GC	isost. graphite	MeCN	AcOH / NBu_4OAc	2.0	0.26	**97**	**65**
**3**	GC	isost. graphite	MeCN	AcOH / NBu_4OAc	3.0	0.38	**96**	**64**
**4**	GC	isost. graphite	MeCN	AcOH / NBu_4OAc	4.0	0.50	**95**	**63**
**5**	GC	isost. graphite	MeCN	AcOH / NBu_4OAc	5.0	0.63	**95**	**63**

[a] GC-quantification against internal standard mesitylene. [b] Residual starting material.[

GP2: Screening of different oxalic esters under optimized conditions

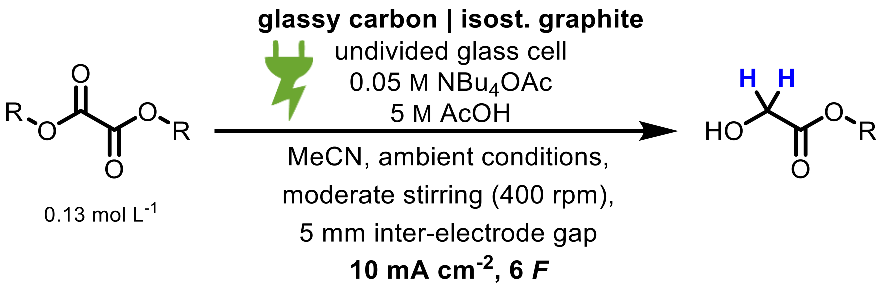

Scheme S 2: Optimized conditions for the screening of different oxalic esters in batch type glass cells.

The 10 mL glass cell equipped with a magnetic stir bar was charged with the corresponding oxalic ester (1.0 mmol). To this, 8 mL of a premade solution of acetic acid (5 M, 58 mL) and tetrabutylammonium acetate (0.05 M, 3.14 g) in MeCN (total volume of 200 mL) was added. Upon dissolution, the electrodes were put into the electrolyte and the galvanostatic electrolysis was started (6.0 *F*, 10 mA cm^{-2}, room temperature, under air, GC (anode), isostatic graphite (cathode), both electrodes 0.8 cm x 5.1 cm, 2 mm inter-electrode gap, moderate stirring (400 rpm)). From 6.0 *F*, Q = 581 *C* is calculated ($Q = n \cdot z \cdot F$). The electrolysis time calculated with $Q = I \cdot t$ and $j = \frac{I}{A}$ is 5 h and 23 mins. After electrolysis, 1,3,5-trimethoxybenzene (168.2 mg, 1.0 mmol) was added and an aliquot dissolved in DMSO-d_6 was measured in the NMR (400 MHz, 30s d_1-relaxtion time). To determine the yield of the electrolysis, the three aromatic protons were correlated to the CH_2-group of the glycolic ester. Isolation of selected examples were done by distillation of the crude mixture from a 10 mmol scale or by column chromatography of a 10 mmol scale.

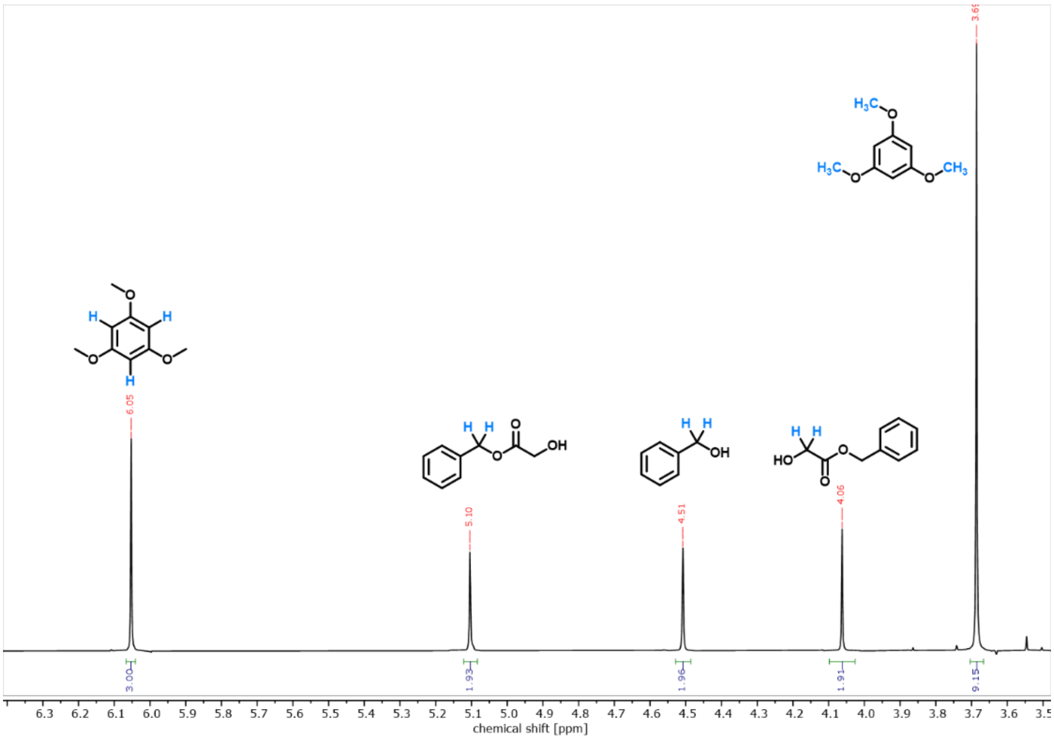

Figure S7: Visualization of the determination of the yield by quantitative ^{1}H NMR using 1,3,5-Trimethoxybenzene as internal standard.

Exemplary evaluation of the yield based on the electrolysis of dibenzyl oxalate (see full spectrum in the spectra attachment).

Table S16: Screening of different oxalic esters under optimized conditions (1 mmol [0.13 M], GC | isost. graphite, 8 mL volume, 6.0 *F*, 10 mA·cm^{-2}, MeCN, 5 M AcOH / 0.05 M NBu_4OAc).

Entry	Starting molecule	Product molecule	^{1}H NMR Yield [%][a]	FE [%]
1	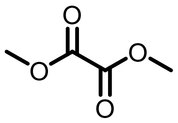	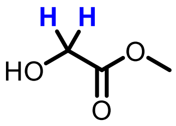	70	47
2	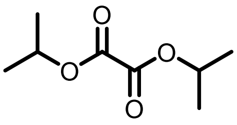	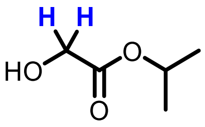	92	61
3	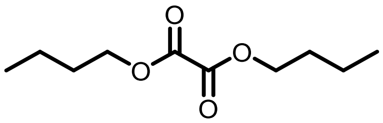	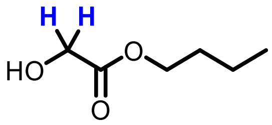	91	61
4	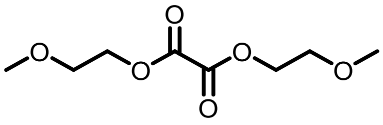	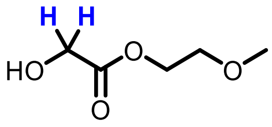	96	64

**5**	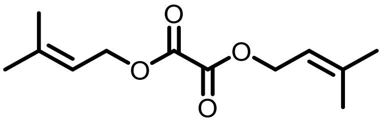	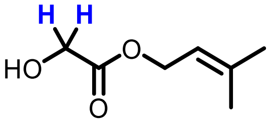	**86**	**57**
**6**	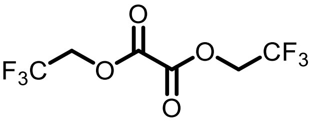	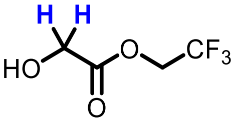	**64**	**43**
**7**	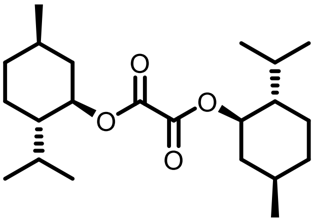	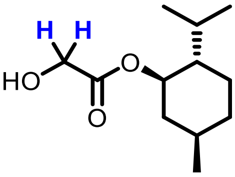	**80**	**53**
**8**	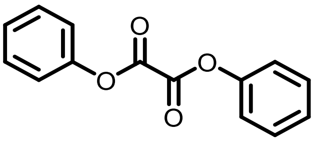	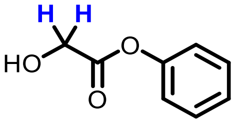	**84**	**56**
**9**	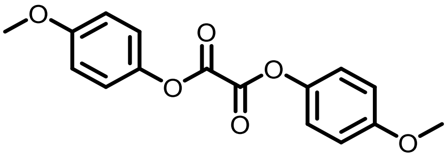	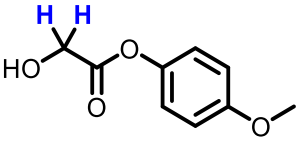	**61[b]**	**41**
**10**	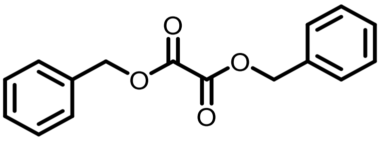	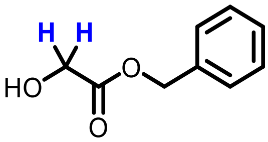	**96**	**64**
**11**	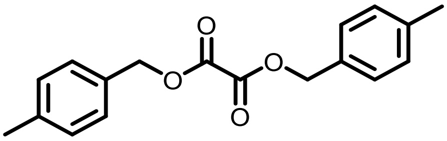	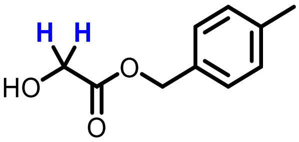	**90**	**60**
**12**	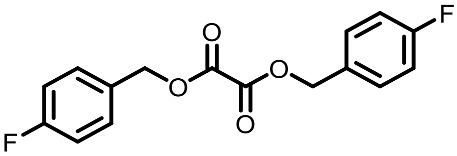	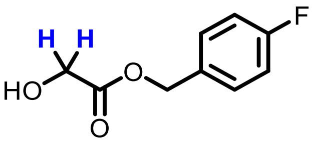	**90**	**60**
**13**	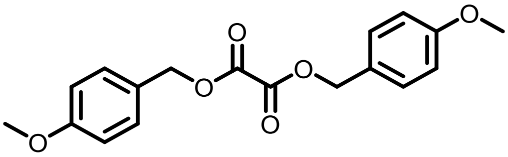	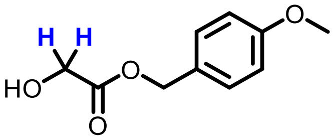	**27[b]**	**18**

[a] Quantification by **^{1}H NMR** using 1,3,5-Trimethoxybenzene as internal standard (1.0 mmol, 168.2 mg. [b] Solubility issues (not suitable for flow electrolysis.)

GP3: Optimization of the diethyl oxalate reduction in flow electrolysis

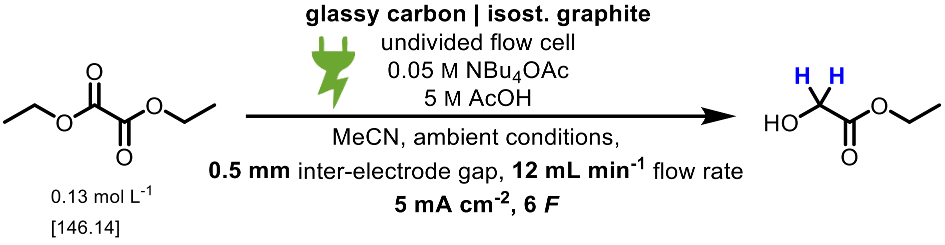

Scheme S3: Optimized conditions of the electrochemical conversion of diethyl oxalate into ethyl 2-hydroxyacetate in flow electrolysis.

To a 50 mL round bottom flask charged with diethyl oxalate (1.0 mmol, 146.1 mg), 8 mL of a premade solution of acetic acid (5 M, 58 mL) and tetrabutylammonium acetate (0.05 M, 3.14 g) in MeCN (total volume of 200 mL) was added two tubing were put into the solution (inlet and outlet of the flow-cell). After filling up the flow cell the galvanostatic electrolysis was started (6.0 *F*, 5 mA cm^{-2}, room temperature, under air, GC (anode), isostatic graphite (cathode), both electrodes 2.0 cm x 6.0 cm, 0.5 mm inter-electrode gap, 12 mL min^{-1} flow rate, cyclic flow). From 6.0 *F*, Q = 581 *C* is calculated ($Q = n \cdot z \cdot F$). The electrolysis time calculated with $Q = I \cdot t$ and $j = \frac{I}{A}$ is 2 h and 41 mins. After electrolysis is completed, 50 μL of mesitylene as internal standard were added to the mixture. A part of the solution was filtered through a small silica column to remove the supporting electrolyte before the sample was analyzed by GC-chromatography. Deviations from the optimized conditions (Scheme S3) are shown in the following tables.

Table S17: Screening of different inter-electrode gaps.

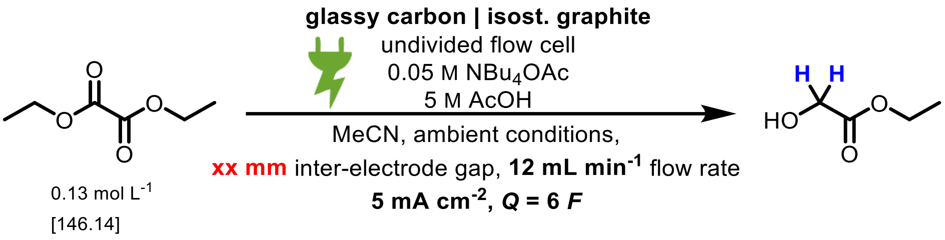

Entry	Electrode distance [mm]	Flow rate [mL min^{-1}]	Current density *j* [mA cm^{-2}]	Applied charge *Q* [*F*]	c_{SM} [mmol mL^{-1}]	GC yield [%][a]	FE [%]
1	0.5	12	5	6.0	0.13	**95**	**63**
2	1.0	12	5	6.0	0.13	**85**	**57**
3	1.5	12	5	6.0	0.13	**79**	**53**
4	2.0	12	5	6.0	0.13	**78**	**52**

[a] GC quantification against internal standard mesitylene. [b] Residual starting material.

Table S18: Screening of different flow rates.

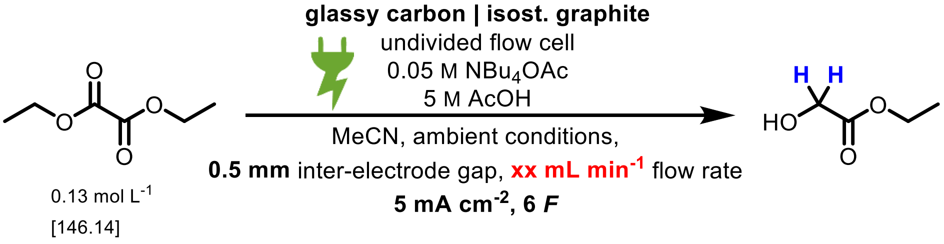

Entry	Flow rate [mL min^{-1}]	Current density j [mA cm^{-2}]	Applied charge Q [F]	c_{SM} [mmol mL^{-1}]	GC yield [%][a]	FE [%]
**1**	12	5	6.0	0.13	**95**	**63**
**2**	0.05 (single pass)	5	6.0	0.13	**0 (95[b])**	**-**
**3**	5.0	5	6.0	0.13	**84**	**56**
**4**	10.0	5	6.0	0.13	**91**	**61**
**5**	15.0	5	6.0	0.13	**93**	**62**
**6**	20.0	5	6.0	0.13	**92**	**61**

[a] GC quantification against internal standard mesitylene. [b] Residual starting material.

Table S19: Screening of different current densities.

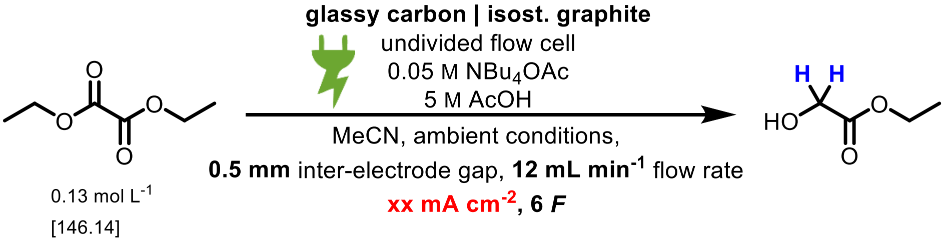

Entry	Current density j [mA cm^{-2}]	Applied charge Q [F]	c_{SM} [mmol mL^{-1}]	GC yield [%][a]	FE [%]
**1**	5	6.0	0.13	**95**	**63**
**2**	10	6.0	0.13	**84**	**56**
**3**	15	6.0	0.13	**76**	**51**
**4**	20	6.0	0.13	**72**	**48**
**5**	25	6.0	0.13	**68**	**45**

[a] GC quantification against internal standard mesitylene. [b] Residual starting material.

In addition, a kinetic experiment was carried out to prove the amount of applied charge given from the batch cell optimization.

Table S20: Conditions for the kinetic flow electrolysis experiment.

Entry	Electrode distance [mm]	Flow rate [mL min^{-1}]	Current density j [mA cm^{-2}]	Applied charge Q [F]	n_{SM} [mmol]	c_{SM} [mmol mL^{-1}]
**1**	0.5	12	5	8.0	2.0	0.26

Each sample represents the equivalent of **1 F mol^{-1}** which lead to 8 sample (each 0.1 mL) + 5 µL of internal standard as a reference. The second figure shows the reach of a plateau starting at around **6 *F***, indicating no further improvement of the yield with more applied charge.

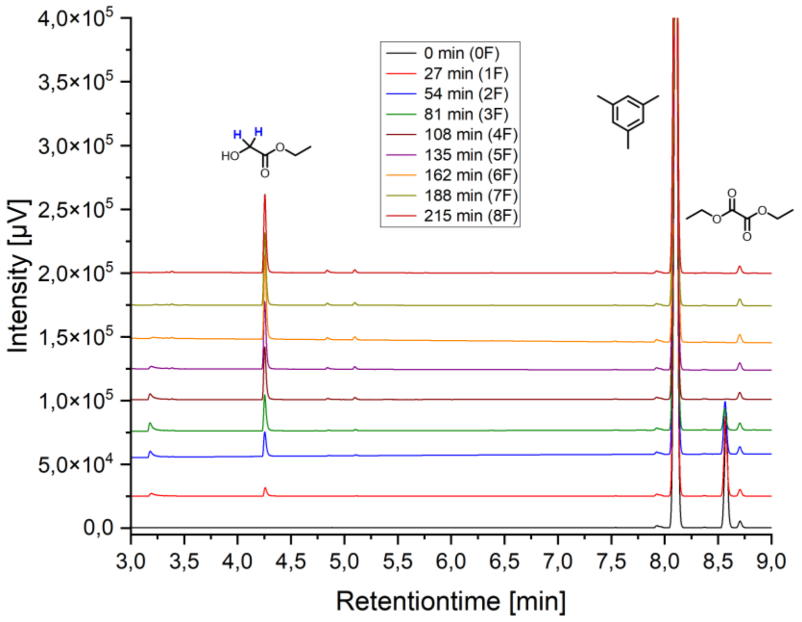

p

Figure S8: Combined GC-chromatograms of each sample of the kinetic flow electrolysis experiment.

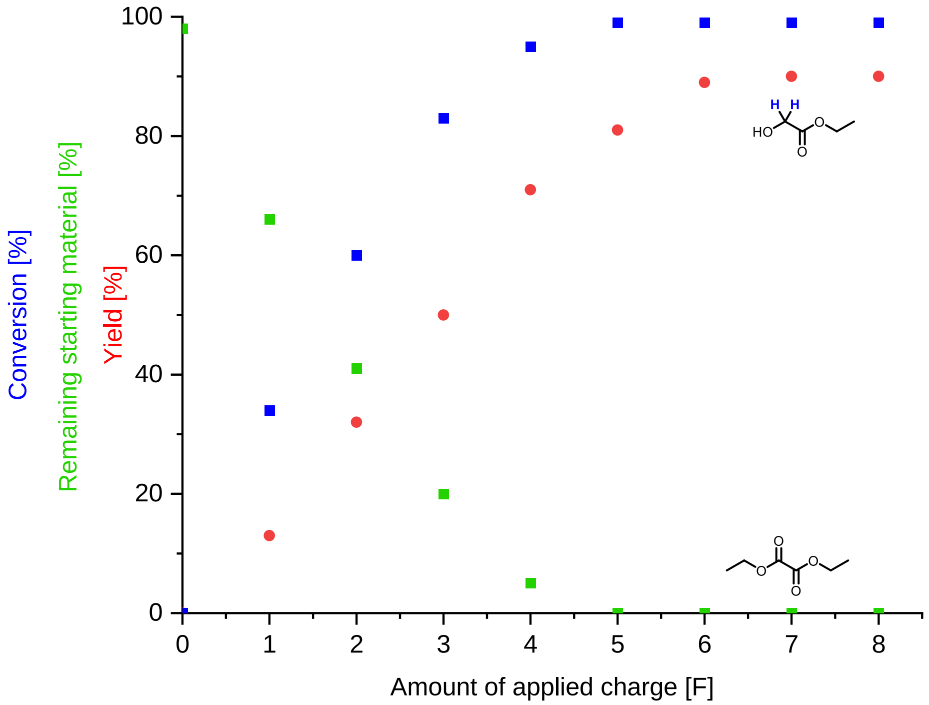

Figure S9: Plotting of the calculated yield and the conversion of the starting material according to the GC-calibration depending on the amount of applied charge.

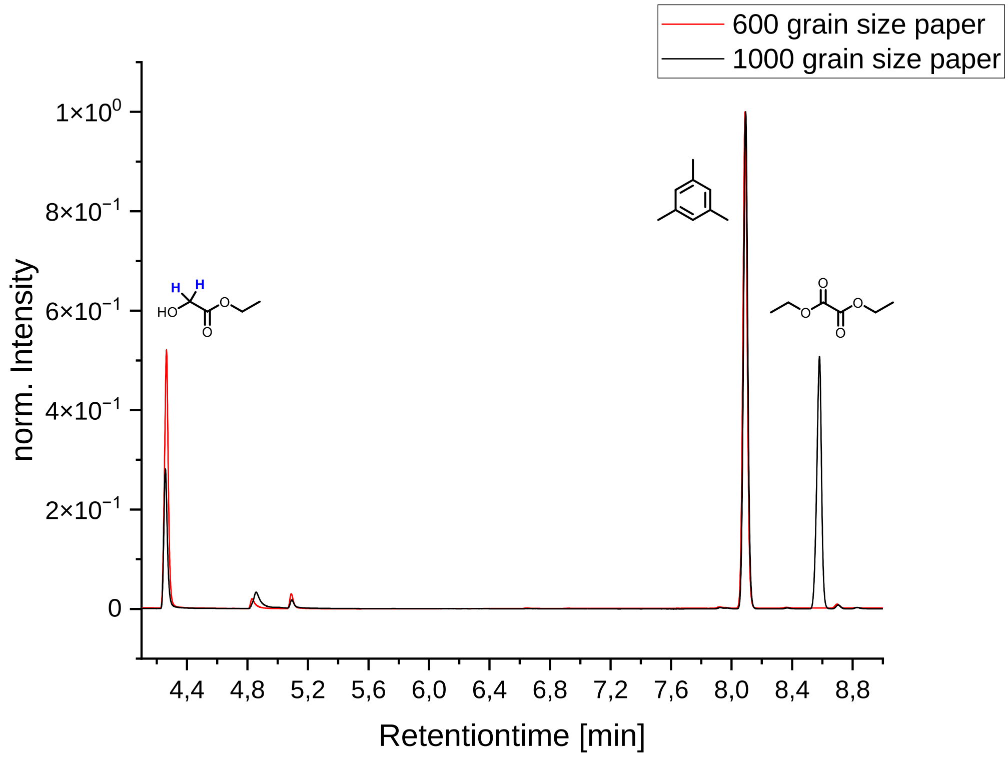

Figure S10: Influence of different sandpaper grain sizes preparing the surface of the graphite electrode on the flow electrolysis of diethyl oxalate into ethyl 2-hydroxyacetate under exact same electrolysis conditions (GC|isost. graphite, 0.5 mm inter-electrode distance, 6 *F*, 5 mA·cm^{-2}, 12 mL min^{-1} flow rate, 5 M AcOH / 0.05 M NBu_4OAc, MeCN).

GP4: Electrolytic reduction of diethyl oxalate in batch cell using deuterated compounds.

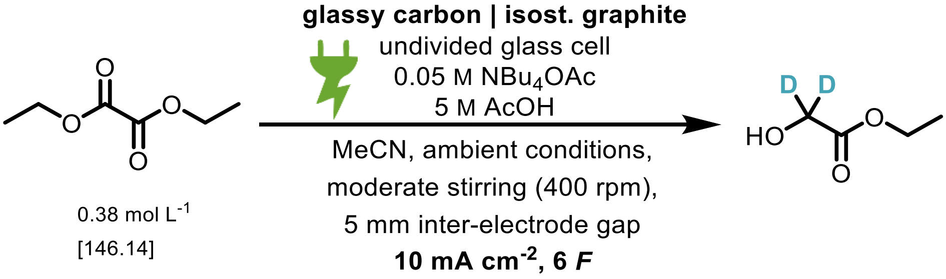

Scheme S4: Reaction conditions applied for the investigation of the deuterium incorporation.

The 10 mL glass cell equipped with a magnetic stir bar was charged with diethyl oxalate (3.0 mmol, 438.3 mg). To this pre-dried (in vacuum, 60°C out of MeCN-d_3 and D_2O) tetrabutylammonium acetate (0.4 mmol, 121 mg) and $MeCN_{dry}$ (6.5 mL) were added quickly. Finally, acetic acid-d_1 or d_4 (1.5 mL) were added. Alternatively, instead of tetrabutylammonium acetate 1.5 mL of a regular ammonium acetate-d_7 stock solution (25 mL acetic acid-d_4 and 220 mg ammonium acetate-d_7) was used. Upon dissolution, the electrodes were put into the electrolyte and the galvanostatic electrolysis was started (6.0 *F*, 10 mA cm^{-2}, room temperature, under air, GC (anode), isostatic graphite (cathode), both electrodes 0.8 cm x 5.1 cm, 5 mm inter-electrode gap, moderate stirring (400 rpm)). From 6.0 *F*, Q = 1743 *C* is calculated ($Q = n \cdot z \cdot F$). The electrolysis time calculated with $Q = I \cdot t$ and $j = \frac{I}{A}$ is 16 h and 08 mins. After electrolysis is completed, a part of the solution was filtered through a small silica column

to remove the supporting electrolyte before the sample was analyzed by HPLC-MS in order to determine the degree of deuteration incorporation shown in Table S22.

Table S21: Optimization of the deuterium incorporation in the final product.

Entry	Solvent	Acid	Supporting Electrolyte	2D [%]	1D [%]	0D [%]	Overall D [%]
1	MeCN-d_3	AcOH	NBu_4OAc	0	0	>99	0
2	MeCN	$AcOD_4$	NBu_4OAc	86	13	<1	93
3	MeCN-d_3	$AcOD_4$	NBu_4OAc	92	8	<0.5	96
4	MeCN-d_3	$AcOD_1$	NBu_4OAc	91	8	<1	95
5[a]	MeCN-d_3	$AcOD_4$	ND_4OAc-(d_3)	96	4	<0.5	98
6[a]	MeCN-d_3	$AcOD_4$	NBu_4OAc	94	6	<0.5	97
7[a]	MeCN-d_3	$AcOD_1$	NBu_4OAc	94	6	<0.5	97
8[b]	MeCN-d_3	$AcOD_1$	NBu_4OAc	94	6	<0.5	97
9[c]	$MeCN_{dry}$	$AcOD_1$	NBu_4OAc	93	6	< 1	97

[a] Conditioning of the glass cell by D_2O/D_2SO_4 treatment overnight in order to remove adsorbed protons on the glass wall and exchange to deuterons. [b] Pre-treatment of the glass cell by using Trimethylsilyl chloride in order to fully dry the cell. [c] MeCN extra dry with septum over molecular sieves.

It seems, that there is no influence whether using acetic acid-d_1 or -d_4. Since acid-d_1 is less expensive than the fully deuterated one, further experiments were conducted by using acetic acid-d_1. Same applies to the used solvent, MeCN. It is not necessary to use deuterated solvent. Therefore, dry MeCN (Acro Seal) was further used.

To make sure, that no hydrogen isotope exchange reaction (HIE) occur, control experiments were carried out. It was checked, whether acidic or basic conditions lead to a HIE.

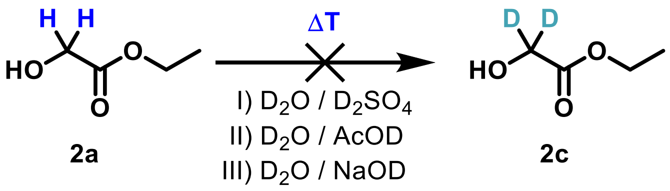

Table S22: Control reactions for the HIE.

Entry	Solvent	0D [%] NMR	Comment
**I)**	D_2O/D_2SO_4	> 99	Ester hydrolysis
**II)**	D_2O/AcOD	> 99	Partial ester hydrolysis
**III)**	D_2O/NaOD	> 99	Saponification

Control experiment before analyzing by ^{1}H NMR.: 1 mmol substrate (146 mg), 1 mL AcOD or D_2SO_4 or 40 %-wt NaOD, 7 mL D_2O at 80 °C, 24h.

The depicted ^{1}H NMR shows the reaction mixture of entry 2 after 24h. an ester hydrolysis towards the free carboxylic acid and the corresponding alcohol is clearly visible. However, due to the structure of the signals, and the ^{2}H NMR a HIE at the desired position can be ruled out.

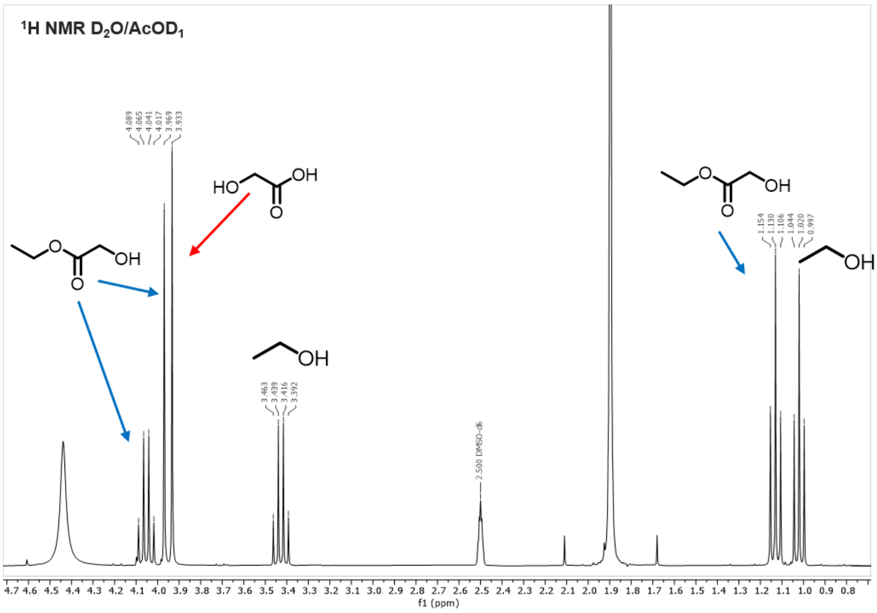

Figure S11: ^{1}H NMR Spectrum of entry 2 indicating partial ester hydrolysis but no HIE.

2. Mechanistic studies on the reduction of oxalic esters in acetic acid solution

Since Kolbe electrolysis is well known in literature and therefore was not deeply investigated, the experiment conducted was the exchange of acetic acid against hexanoic acid. In a system comprising 5 M acetic acid dissolved in acetonitrile and containing 0.05 M tetrabutylammonium acetate, the Kolbe electrolysis can proceed efficiently. The presence of tetrabutylammonium acetate ensures a sufficiently high concentration of free carboxylate ions, which constitute the electrochemically active species in the reaction.[2-3] At high concentrations, acetic acid acts as a reaction partner: it enables rapid proton transfer after electron donation, stabilizes the resulting radicals, and buffers the system without suppressing the Kolbe reaction.[4] The use of a water free, aprotic solvent suppresses competing oxygen evolution, thereby enabling the application of high anodic potentials.[5] Furthermore, the acidity of acetic acid is markedly diminished in acetonitrile, preventing inhibition of acetate oxidation and instead serving primarily as a reaction medium. However, the detection of CO_2 and ethane was not successful. For this reason, acetic acid was replaced with hexanoic acid. Unlike acetic acid, hexanoic acid undergoes Kolbe electrolysis even under acidic bulk conditions. Its higher hydrophobicity promotes accumulation at the electrode interface, facilitating local deprotonation within the electrical double layer.[6] The resulting alkyl radicals exhibit greater stability than methyl radicals, enhancing their recombination to the Kolbe dimer. The resulting product from this reaction, n-decane, which corresponds to the dimerization of two pentyl radicals is therefore easier to detect and was successfully analyzed using GC and GCMS.

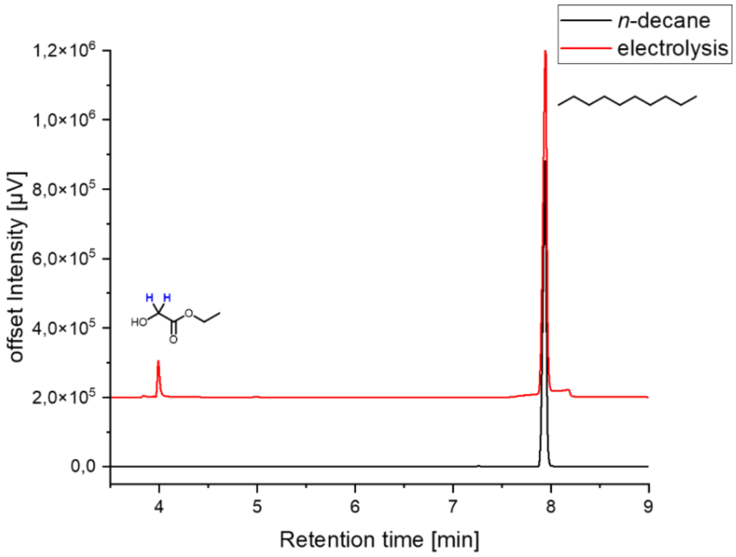

Figure S12: GC chromatogram of the electrolysis mixture after the reaction (red) against the reference of *n*-decane (black).

In addition, no dehydration reaction of the acetic acid to it acetic acid anhydride was observed in the raw ^{13}C NMR. According to literature spectra of acetic anhydride, the ^{13}C signal of the carboxylic acid should appear around 165-170. Seen in the reaction mixture spectrum, no signal in this region is observed.

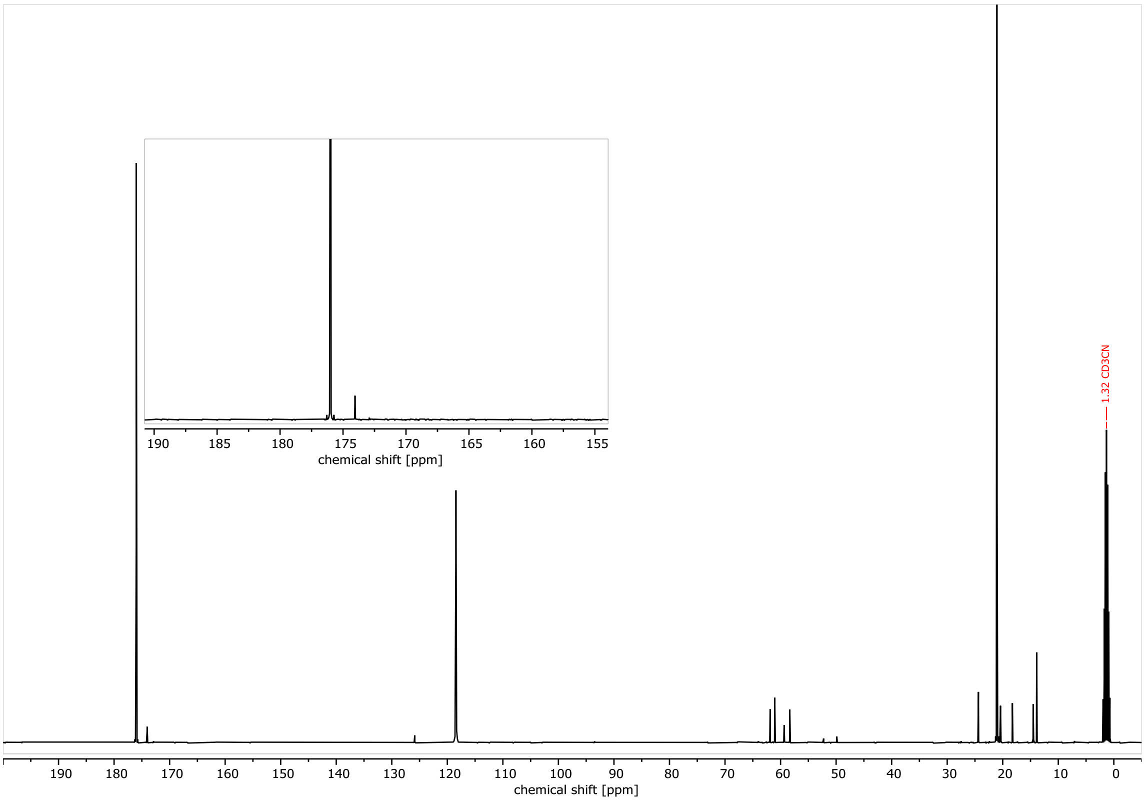

Figure S13: ^{13}C NMR spectrum of the crude reaction mixture.

On the cathodic side, the reduction of the oxalic ester was investigated by GC and NMR. Since trapping of the aldehyde intermediate is rather difficult, it was monitored by GC.

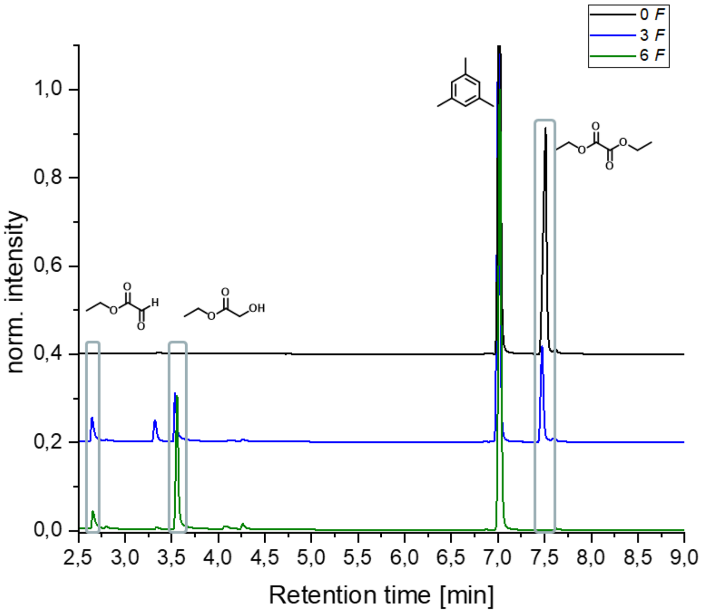

Figure S14: GC chromatogram of the electrolysis after different amounts of applied charge. The formation of the aldehyde intermediate can be seen on the left-hand side of the chromatogram.

To further investigate the reduction, it is additionally possible to detect the formed alcohol (ester cleavage) in the reaction.

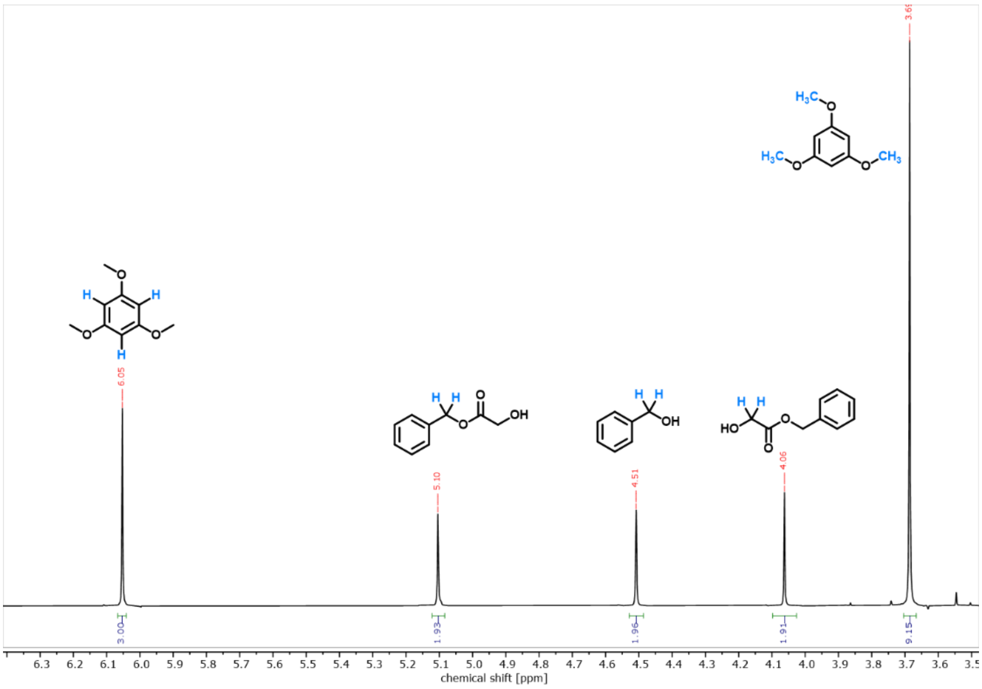

Figure S15: ^{1}H NMR after the electrolysis of dibenzyl oxalate. The formation of benzyl alcohol can be observed.

Indicating the formation of the alcohol leads to the assumption, that the ester is cleaved by forming the aldehyde, which will then be reduced further to the corresponding alcohol.

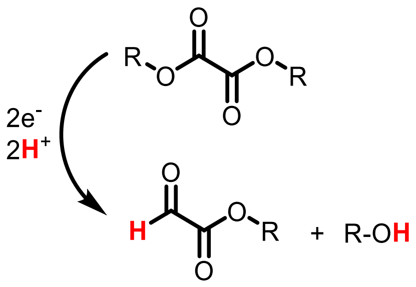

Formation of the aldehyde as the initial reduction step.

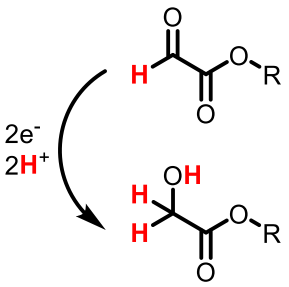

Further reduction of the aldehyde to the corresponding alcohol.

4. Synthesis of oxalic esters

GP5: General synthesis of different alkyl and aryl oxalates

In a 2-neck round bottom flask, the corresponding alcohol (82 mmol, 2.1 eq.) was dissolved with triethylamine (82 mmol, 2.1 eq.) in THF (100 mL) under stirring. The mixture was then cooled to about 5°C using an ice-water bath. Oxalyl chloride (40 mmol, 1 eq.) was added dropwise under stirring and cooling. After complete addition, stirring was continued for several hours at room temperature. Water was then added to the reaction solution to dissolve the formed triethylammonium hydrochloride salt. The two-phase mixture was extracted with diethyl ether (3x30 mL), washed with brine and dried over sodium sulphate. Purification was carried out by distillation or recrystallization.

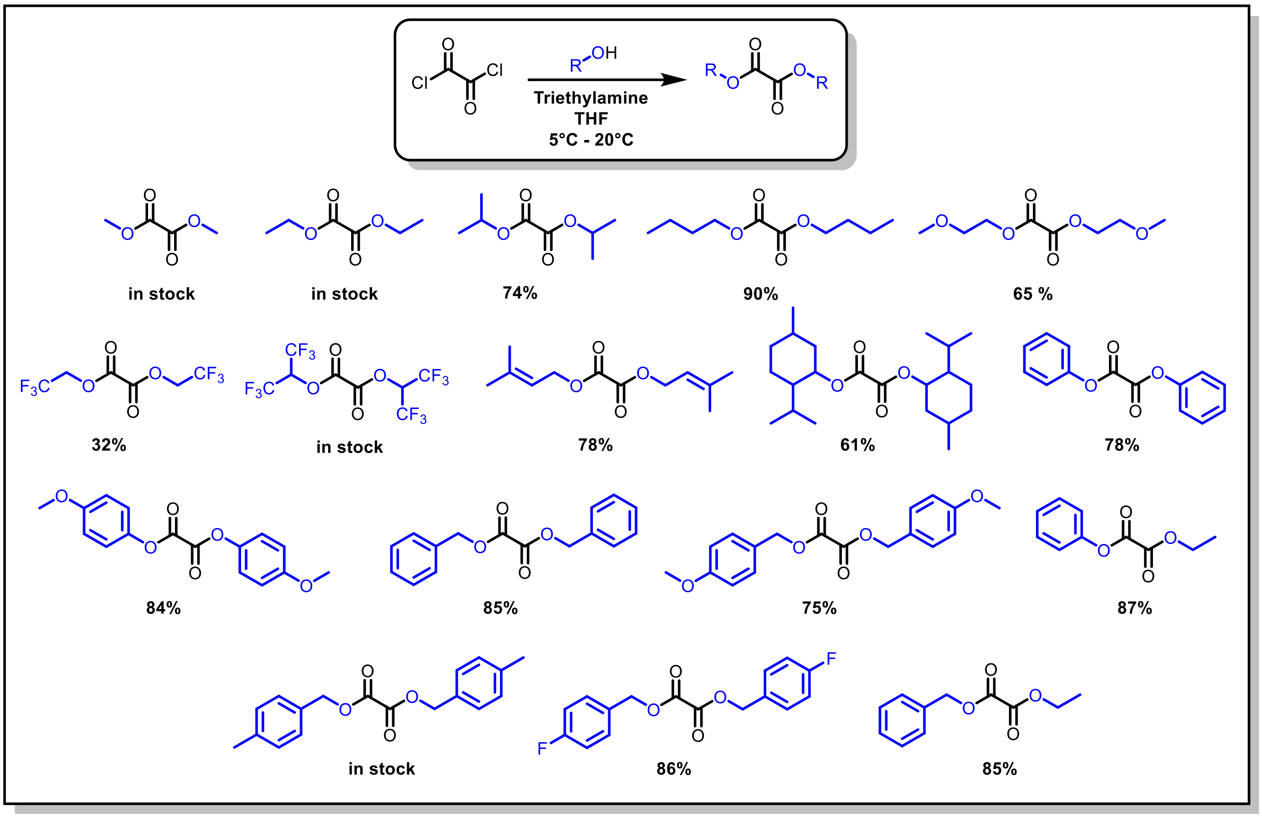

Scheme S5: Overview of the commercially bought and synthesized oxalic esters.

**Dimethyl oxalate**	
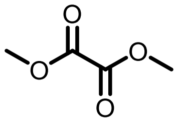	
Dimethyl oxalate was bought from Sigma Aldrich. ^{1}H NMR for reference purposes.	
**^{1}H NMR**	(400 MHz, DMSO-d_6) δ [ppm]: 3.78 (s, 6H).

The analytical data are in accordance with the literature.[7]

Diethyl oxalate

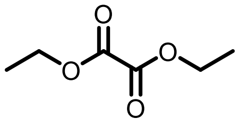

Diethyl oxalate was bought from Sigma Aldrich and TCI. ^{1}H NMR for reference purposes.

**^{1}H NMR**	(400 MHz, $CDCl_3$) δ [ppm]: 4.32 (q, 4H, ^3J = 7.1 Hz), 1.34 (t, 6H, ^3J = 7.1 Hz).

The analytical data are in accordance with the literature.[8]

Diisopropyl oxalate

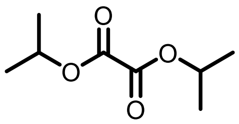

GP5 was followed for synthesis. The product was obtained by vacuum distillation (5 mbar, 60-65°C) as a colorless liquid (74%).

**Yield**	74%
**^{1}H NMR**	(400 MHz, DMSO-d_6) δ [ppm]: 5.03 (hept, 2H, ^3J = 6.3 Hz), 1.27 (d, 12H, ^3J = 6.3 Hz).
**^{13}C NMR**	(101 MHz, DMSO-d_6) δ [ppm]: 157.3, 70.8, 21.2.
**GC-MS (EI)**	([M] m/z): **calc**: 174.09 **found**: fragments: 39.00, 45.00, 59.00.

The analytical data are in accordance with the literature.[9]

Dibutyl oxalate

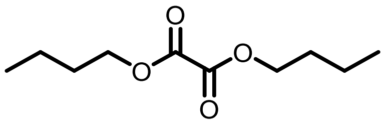

GP5 was followed for synthesis. The product was obtained by vacuum distillation (5 mbar, 98°C) as a colorless liquid (90%).

**Yield**	90%
**^{1}H NMR**	(400 MHz, DMSO-d_6) δ [ppm]: 4.22 (t, 4H, ^3J = 6.6 Hz), 1.63 (m, 4H), 1.35 (m, 4H), 0.89 (t, 6H, ^3J = 7.4 Hz).
**^{13}C NMR**	(101 MHz, DMSO-d_6) δ [ppm]: 157.5, 66.1, 29.7, 18.4, 13.4.
**GC-MS (EI)**	([M] m/z): **calc**: 202.12 **found**: 202.00.

The analytical data are in accordance with the literature.[10]

Bis(2-methoxyethyl) oxalate

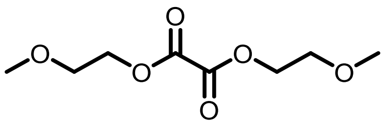

GP5 was followed for the synthesis of bis(2-methoxyethyl)-oxalate. The product was obtained by recrystallization as colorless needles (65%).

**Yield**	65%
**^{1}H NMR**	(400 MHz, DMSO-d_6) δ [ppm]: 4.35 (m, 4H), 3.59 (m, 4H), 3.27 (s, 6H).
**^{13}C NMR**	(101 MHz, DMSO-d_6) δ [ppm]: 157.3, 69.2, 65.5, 58.0.
**GC-MS (EI)**	([M] m/z): **calc**: 206.08 **found**: 103.00 (1/2 m/z).

The analytical data are in accordance with the literature.[9]

Bis(3-methylbut-2-en-1-yl) oxalate

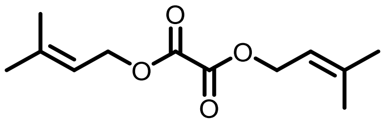

GP5 was followed for the synthesis of bis(3-methylbut-2-en-1-yl) oxalate. The product was obtained as a yellow liquid (78%). It was used without further purification.

**Yield**	78%
**^{1}H NMR**	(400 MHz, DMSO-d_6) δ [ppm]: 5.35 (tdt, 2H), 4.72 (d, 4H, ^{3}J = 7.4 Hz), 1.72 (dd, 12H, ^{4}J = 1.4 Hz).
**^{13}C NMR**	(101 MHz, DMSO-d_6) δ [ppm]: 157.4, 140.3, 117.4, 63.1, 25.4, 17.8.
**GC-MS (EI)**	([M] m/z): **calc**: 226.12 **found**: 226.00.

The analytical data are in accordance with the literature.

Bis(2,2,2-trifluoroethyl) oxalate

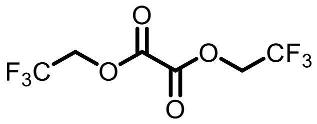

GP5 was followed for the synthesis of bis(2,2,2-trifluoroethyl) oxalate. The product was obtained by vacuum distillation (6 mbar, 65°C) as a colorless liquid (32%).

**Yield**	32%
**^{1}H NMR**	(400 MHz, $CDCl_3$) δ [ppm]: 4.97 (q, ^{3}J = 8.9 Hz, 4H).
**^{13}C NMR**	(101 MHz, $CDCl_3$) δ [ppm]: 154.5, 123.1 (q, ^{2}J = 277.0 Hz, CF_3), 61.9 (q, ^{3}J = 36.0 Hz).
**^{19}F NMR**	(376 MHz, $CDCl_3$) δ [ppm]: -73.38 (d, ^{3}J = 9.0 Hz)
**GC-MS (EI)**	([M] m/z): **calc**: 254.00 **found**: 127.00 (1/2 m/z).

The analytical data are in accordance with the literature.[11]

Bis(menthyl) oxalate

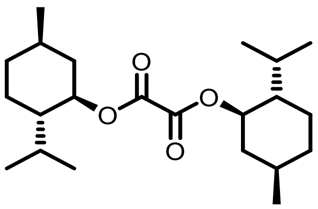

GP5 was followed for the synthesis of bis(2-isopropyl-5-methylcyclohexyl) oxalate. The product was obtained as a colorless solid (61%).

**Yield**	61%
**^{1}H NMR**	(400 MHz, $CDCl_3$) δ [ppm]: 4.85 – 4.78 (m, 2H), 2.08 – 2.02 (m, 2H), 1.91 – 1.84 (m, 2H), 1.73 – 1.66 (m, 4H), 1.55-1.46 (m, 4H), 1.16 – 1.02 (m, 4H), 0.89 (m, 12H), 0.77 (d, 6H, ^3J = 7.0 Hz).
**^{13}C NMR**	(101 MHz, $CDCl_3$) δ [ppm]: 158.3, 77.7, 46.8, 40.4, 34.2, 31.6, 26.5, 23.7, 22.1, 20.7, 16.6.
**GC-MS (EI)**	([M] m/z): **calc**: 366.28 **found**: 366.30.

The analytical data are in accordance with the literature.

Diphenyl oxalate

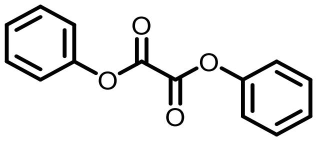

GP5 was followed for the synthesis of diphenyl oxalate. The product was obtained by recrystallisation from cyclohexane/ethyl acetate as a colorless solid (78%).

**Yield**	78%
**^{1}H NMR**	(400 MHz, DMSO-d_6) δ [ppm]: 7.54 – 7.49 (m, 4H), 7.38 – 7.31 (m, 6H).
**^{13}C NMR**	(101 MHz, DMSO-d_6) δ [ppm]: 155.0, 150.1, 129.8, 126.7, 121.3.
**GC-MS (EI)**	([M] m/z): **calc**: 242.06 (100%), 243.06 (15%) **found**: 242.00 (100%), 243.05 (17%).

The analytical data are in accordance with the literature.[11]

Bis(4-methoxyphenyl) oxalate

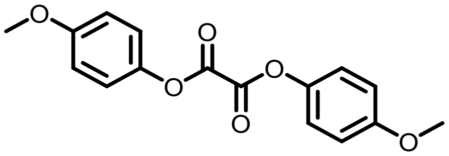

GP5 was followed for the synthesis of bis(4-methoxyphenyl) oxalate. The product was obtained by recrystallisation from cyclohexane/ethyl acetate as a colorless solid (84%).

**Yield**	84%
**^{1}H NMR**	(400 MHz, $CDCl_3$) δ [ppm]: 7.25 – 7.21 (m, 4H), 7.05 – 7.01 (m, 4H), 3.78 (s, 6H).
**^{13}C NMR**	(101 MHz, $CDCl_3$) δ [ppm]: 157.4, 155.4, 143.5, 122.1, 114.7, 55.5.
**GC-MS (EI)**	([M] m/z): **calc**: 302.08 **found**: 302.00.

The analytical data are in accordance with the literature.[12]

Dibenzyl oxalate

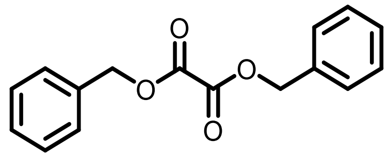

GP5 was followed for the synthesis of dibenzyl oxalate. The product was obtained by recrystallisation from cyclohexane/ethyl acetate as a colorless solid (85%).

**Yield**	85%
**^{1}H NMR**	(400 MHz, DMSO-d_6) δ [ppm]: 7.44 – 7.36 (m, 10H), 5.29 (s, 4H).
**^{13}C NMR**	(101 MHz, DMSO-d_6) δ [ppm]: 157.1 134.7, 128.7, 128.6, 68.0.
**GC-MS (EI)**	([M] m/z): **calc**: 270.09 **found**: 270.00.

The analytical data are in accordance with the literature.[13]

Bis(4-methoxybenzyl) oxalate

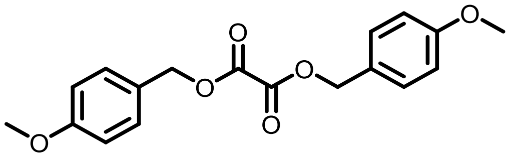

GP5 was followed for the synthesis of bis(4-methoxybenzyl) oxalate. The product was obtained by recrystallisation from cyclohexane/ethyl acetate as a colorless solid (84%).

**Yield**	84%
**^{1}H NMR**	(400 MHz, $CDCl_3$) δ [ppm]: 7.36 – 7.32 (m, 4H), 6.90 – 6.86 (m, 4H), 5.23 (s, 4H), 3.80 (s, 6H).
**^{13}C NMR**	(101 MHz, $CDCl_3$) δ [ppm]: 160.2, 157.8, 130.9, 126.4, 114.5, 68.8, 55.4.
**GC-MS (EI)**	([M] m/z): **calc**: 330.11 **found**: 330.00.

The analytical data are in accordance with the literature.[14]

Bis(4-fluorobenzyl) oxalate

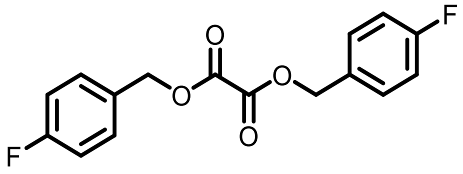

GP5 was followed for the synthesis of bis(4-fluorobenzyl) oxalate. The product was obtained by recrystallisation from cyclohexane/ethyl acetate as a colorless solid (86%).

**Yield**	86%
**^{1}H NMR**	(400 MHz, DMSO-d_6) δ [ppm]: 7.51 – 7.46 (m, 4H), 7.26 – 7.20 (m, 4H), 5.26 (s, 4H).
**^{13}C NMR**	(101 MHz, DMSO-d_6) δ [ppm]: 162.2 (d, J = 244.8 Hz) 156.9, 131,1 (d, ^{4}J = 8.5 Hz), 130.9 (d, ^{5}J = 3.2 Hz), 115.4 (d, ^{3}J = 21.6 Hz), 67.2.
**GC-MS (EI)**	([M] m/z): **calc**: 306.07 **found**: 306.00.

The analytical data are in accordance with the literature.[14]

**Bis(4-methylbenzyl) oxalate**	
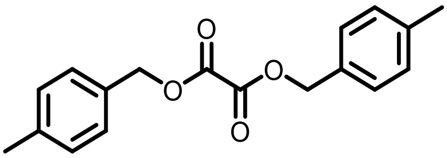	
Bis (4-methylbenzyl) oxalate was bought from BLD-Pharm. ^{1}H NMR and ^{13}C NMR for reference purposes.	
**^{1}H NMR**	(400 MHz, DMSO-*d*$_6$) δ [ppm]: 7.32 – 7.30 (m, 4H), 7.21-7.18 (m,4H), 5.23 (s, 4H), 2.30 (s, 6H).
**^{13}C NMR**	(101 MHz, DMSO-*d*$_6$) δ [ppm]: 157.1, 138.1, 131.6, 129.1, 128.8, 67.9, 20.8.

The analytical data are in accordance with the literature.[14]

**Ethyl phenyl oxalate**	
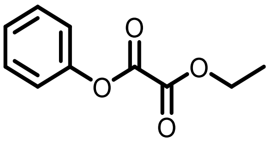	
GP5 was followed for the synthesis of ethyl phenyl oxalate. Instead of oxalyl chloride, ethyl 2-chloro-2-oxoacetate was used. The product was obtained by vacuum (6 mbar, 100°C) distillation as a colorless oil (87%).	
**Yield**	87%
**^{1}H NMR**	(400 MHz, DMSO-*d*$_6$) δ [ppm]: 7.50 – 7.44 (m, 2H), 7.35-7.31 (m, 1H), 7.28-7.25 (m, 2H), 4.35 (q, ^{3}J = 7.1 Hz, 2H), 1.33 (t, ^{3}J = 7.1 Hz, 3H).
**^{13}C NMR**	(101 MHz, DMSO-*d*$_6$) δ [ppm]: 156.6, 155.6, 145.0, 129.8, 126.6, 121.3, 63.0, 13.8.
**GC-MS (EI)**	([M] m/z): **calc**: 194.06 **found**: 194.05.

The analytical data are in accordance with the literature.[9]

**Benzyl ethyl oxalate**	
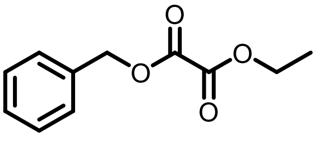	
GP5 was followed for the synthesis of benzyl ethyl oxalate. Instead of oxalyl chloride, ethyl 2-chloro-2-oxoacetate was used. The product was obtained by vacuum distillation (0.02 mbar, 90°C) as a colorless oil (85%).	
**Yield**	85%
**^{1}H NMR**	(400 MHz, DMSO-*d*$_6$) δ [ppm]: 7.45 – 7.36 (m, 5H), 5.29 (s, 2H), 4.27 (q, ^{3}J = 7.1 Hz, 2H), 1.26 (t, ^{3}J = 7.1 Hz, 3H).
**^{13}C NMR**	(101 MHz, DMSO-*d*$_6$) δ [ppm]: 157.2, 134.7, 128.7, 128.6, 128.5, 37.9, 62.8, 13.7.
**GC-MS (EI)**	([M] m/z): **calc**: 208.07 **found**: fragments: 91.00, 107.00.

The analytical data are in accordance with the literature.[11]

5. NMR characterization of isolated products after electrolysis

Ethyl 2-hydroxyacetate

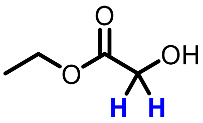

Ethyl 2-hydroxyacetate was isolated by distillation (4 mbar, 45°C) of the crude reaction mixture after electrolysis.

**^{1}H NMR**	(400 MHz, DMSO-d_6) δ [ppm]: 5.29 (t, ^{2}J = 6.5 Hz, 1H, OH), 4.09 (q, ^{3}J = 7.1 Hz, 2H), 3.98 (d, ^{2}J = 6.5 Hz, 2H), 1.19 (t, ^{3}J = 7.1 Hz, 3H).
**^{13}C NMR**	(101 MHz, DMSO-d_6) δ [ppm]: 172.7, 59.9, 59.7, 14.2.
**GC-MS (EI)**	([M] m/z): **calc**: 104.05 **found**: 104.00.

The analytical data are in accordance with the literature.[15]

Ethyl 2-hydroxyacetate-d_2

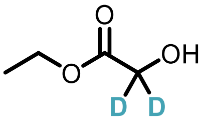

Ethyl 2-hydroxyacetate-d_2 was isolated by distillation (4 mbar, 45°C) of the crude reaction mixture after electrolysis.

**^{1}H NMR**	(400 MHz, DMSO-d_6) δ [ppm]: 5.29 (bs, OH), 4.09 (q, ^{3}J = 7.1 Hz, 2H), 3.96 (m, 0.05 H*), 1.19 (t, ^{3}J = 7.1 Hz, 3H).
**^{13}C NMR**	(101 MHz, DMSO-d_6) δ [ppm]: 172.6, 60.8, 60.00 – 58.6 (m), 14.2.
**GC-MS (EI)**	([M] m/z): **calc**: 106.06 **found**: 106.05.

The analytical data are in accordance with the literature. *Remaining proton integral.

Methyl 2-hydroxyacetate

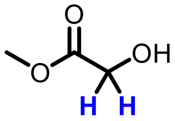

Methyl 2-hydroxyacetate was isolated by distillation (4 mbar, 40°C) of the crude reaction mixture after electrolysis.

**^{1}H NMR**	(400 MHz, DMSO-d_6) δ [ppm]: 5.31 (t, ^{2}J = 6.5 Hz, 1H, OH), 4.00 (d, ^{2}J = 6.5 Hz, 2H), 3.63 (s, 3H).
**^{13}C NMR**	(101 MHz, DMSO-d_6) δ [ppm]: 173.2, 59.7, 51.3.
**GC-MS (EI)**	([M] m/z): **calc**: 90.03 **found**: 90.05.

The analytical data are in accordance with the literature.[15]

Isopropyl 2-hydroxyacetate

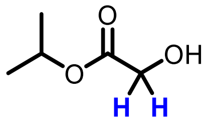

Isopropyl 2-hydroxyacetate was isolated by distillation (5 mbar, 60°C) of the crude reaction mixture after electrolysis.

**^{1}H NMR**	(400 MHz, DMSO-d_6) δ [ppm]: 5.25 (t, ^{2}J = 6.6 Hz, 1H, OH), 4.92 (hept, ^{3}J = 6.3 Hz, 1H), 3.94 (d, ^{2}J = 6.6 Hz, 2H), 1.19 (d, ^{3}J = 6.3 Hz, 6H).
**^{13}C NMR**	(101 MHz, DMSO-d_6) δ [ppm]: 172.2, 67.4, 59.8, 21.7.
**GC-MS (EI)**	([M] m/z): **calc**: 118.06 **found**: 118.00.

The analytical data are in accordance with the literature.[16]

Butyl 2-hydroxyacetate

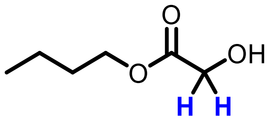

Butyl 2-hydroxyacetate was isolated by distillation (5 mbar, 65°C) of the crude reaction mixture after electrolysis.

**^{1}H NMR**	(400 MHz, DMSO-d_6) δ [ppm]: 5.30 (t, ^{2}J = 6.6 Hz, 1H, OH), 4.05 (t, ^{3}J = 6.6 Hz, 2H), 3.99 (d, ^{2}J = 6.5 Hz, 2H), 1.58 – 1.51 (m, 2H), 1.36 – 1.27 (m, 2H), 0.88 (t, ^{3}J = 7.4 Hz, 3H).
**^{13}C NMR**	(101 MHz, DMSO-d_6) δ [ppm]: 172.8, 63.6, 59.7, 30.3, 18.6, 13.6.
**GC-MS (EI)**	([M] m/z): **calc**: 132.08 **found**: 132.00.

The analytical data are in accordance with the literature.

Benzyl 2-hydroxyacetate

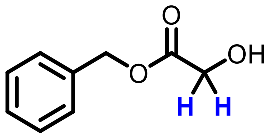

Benzyl 2-hydroxyacetate was isolated by flash chromatography of the crude evaporated mixture.

**^{1}H NMR**	(400 MHz, DMSO-d_6) δ [ppm]: 7.40 – 7.31 (m, 5H), 5.39 (t, ^{2}J = 6.5 Hz, 1H, OH), 5.14 (s, 2H), 4.07 (d, ^{2}J = 6.3 Hz, 2H).
**^{13}C NMR**	(101 MHz, DMSO-d_6) δ [ppm]: 172.6, 136.1, 128.5, 128.1, 128.1, 65.5, 59.7.
**GC-MS (EI)**	([M] m/z): **calc**: 166.06 **found**: 166.05.

The analytical data are in accordance with the literature.[17]

6. GC spectra of the starting materials

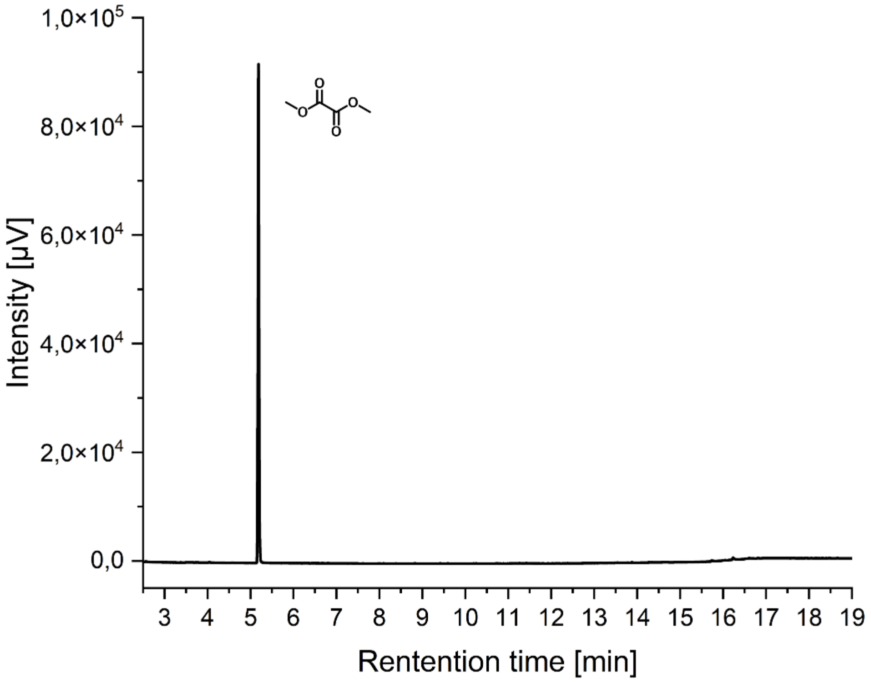

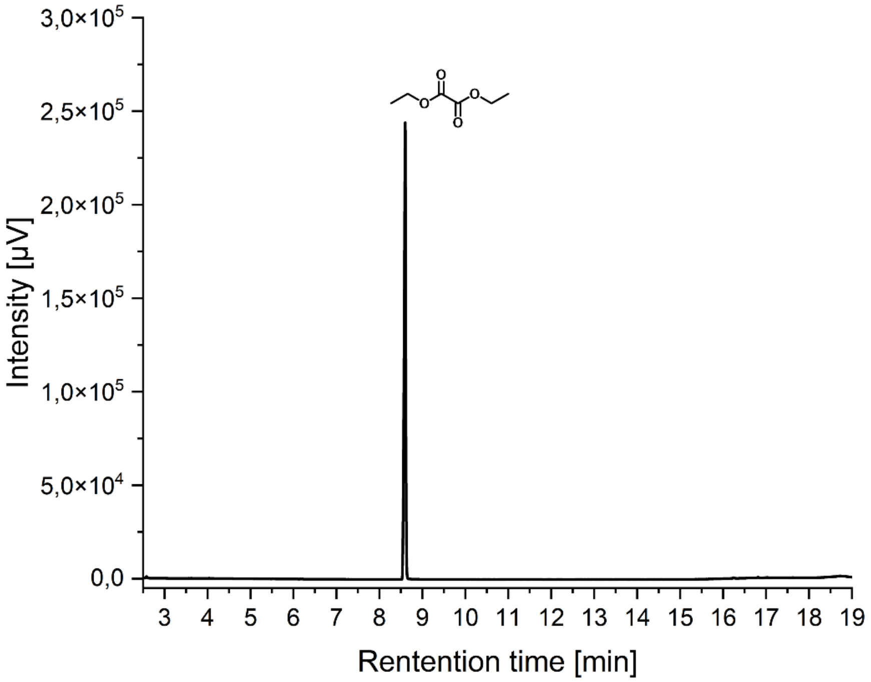

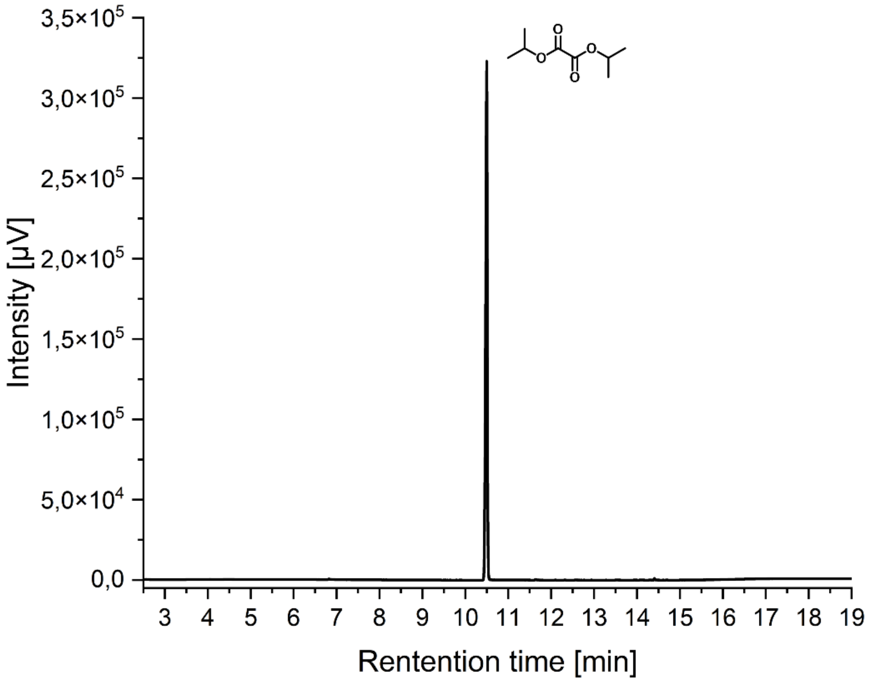

3,5×10^5
3,0×10^5
2,5×10^5
2,0×10^5
1,5×10^5
1,0×10^5
5,0×10^4
0,0
Intensity [µV]
3 4 5 6 7 8 9 10 11 12 13 14 15 16 17 18 19
Rentention time [min]

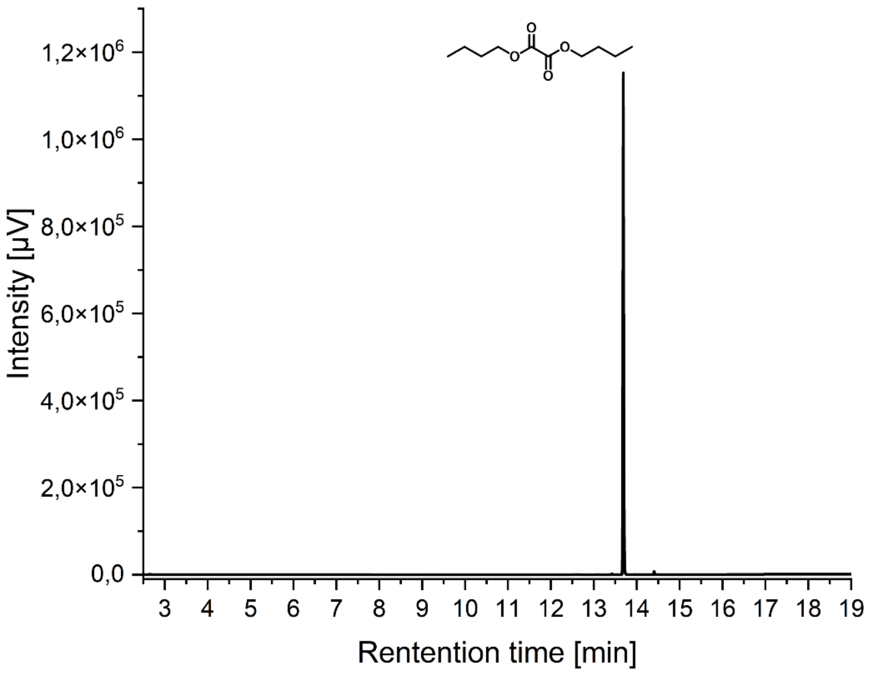

1,2×10^6
1,0×10^6
8,0×10^5
6,0×10^5
4,0×10^5
2,0×10^5
0,0
Intensity [µV]
3 4 5 6 7 8 9 10 11 12 13 14 15 16 17 18 19
Rentention time [min]

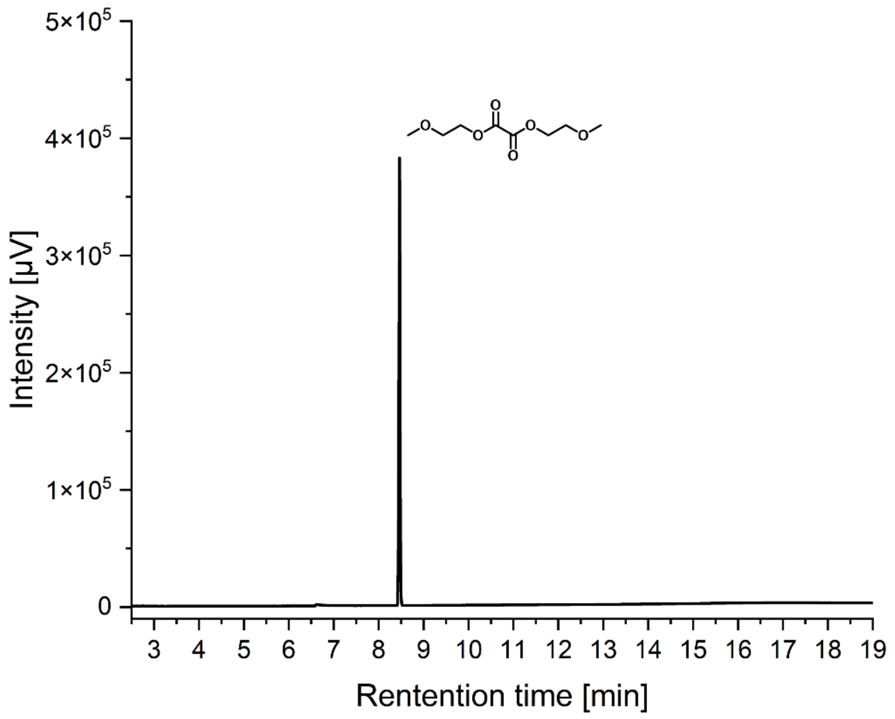

5×10^5
4×10^5
3×10^5
2×10^5
1×10^5
0
Intensity [µV]
3 4 5 6 7 8 9 10 11 12 13 14 15 16 17 18 19
Rentention time [min]

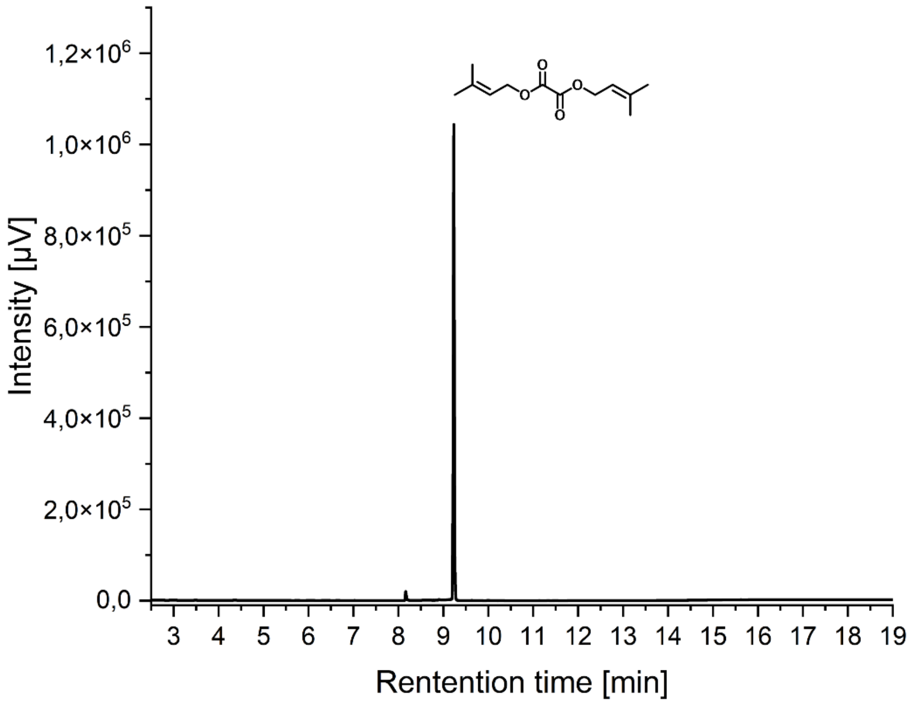

1,2×10^6
1,0×10^6
8,0×10^5
6,0×10^5
4,0×10^5
2,0×10^5
0,0
Intensity [µV]
3 4 5 6 7 8 9 10 11 12 13 14 15 16 17 18 19
Rentention time [min]

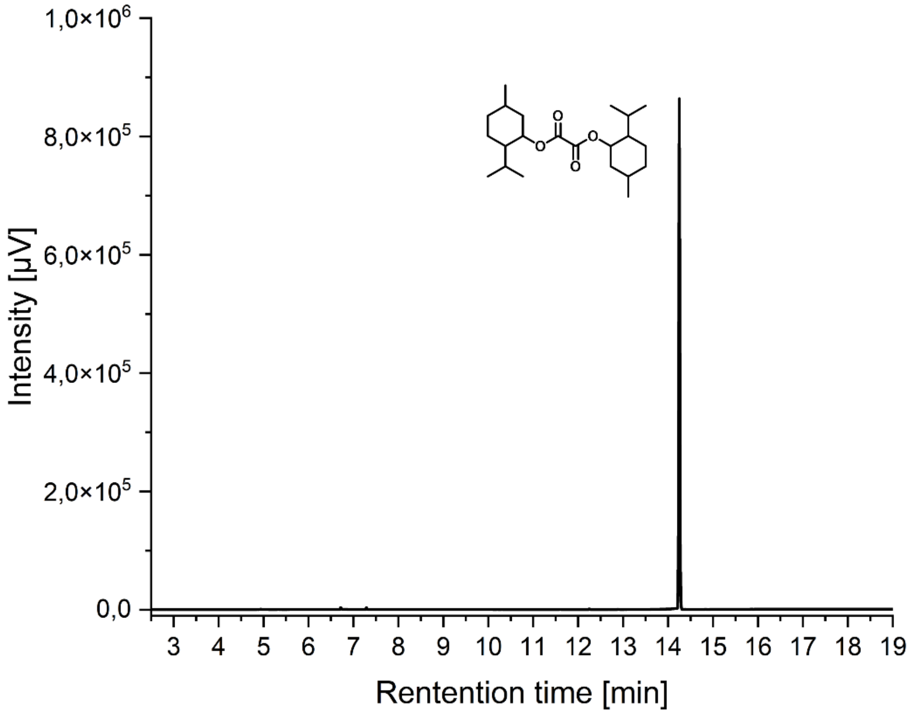

1,0×10^6
8,0×10^5
6,0×10^5
4,0×10^5
2,0×10^5
0,0
Intensity [µV]
3 4 5 6 7 8 9 10 11 12 13 14 15 16 17 18 19
Rentention time [min]

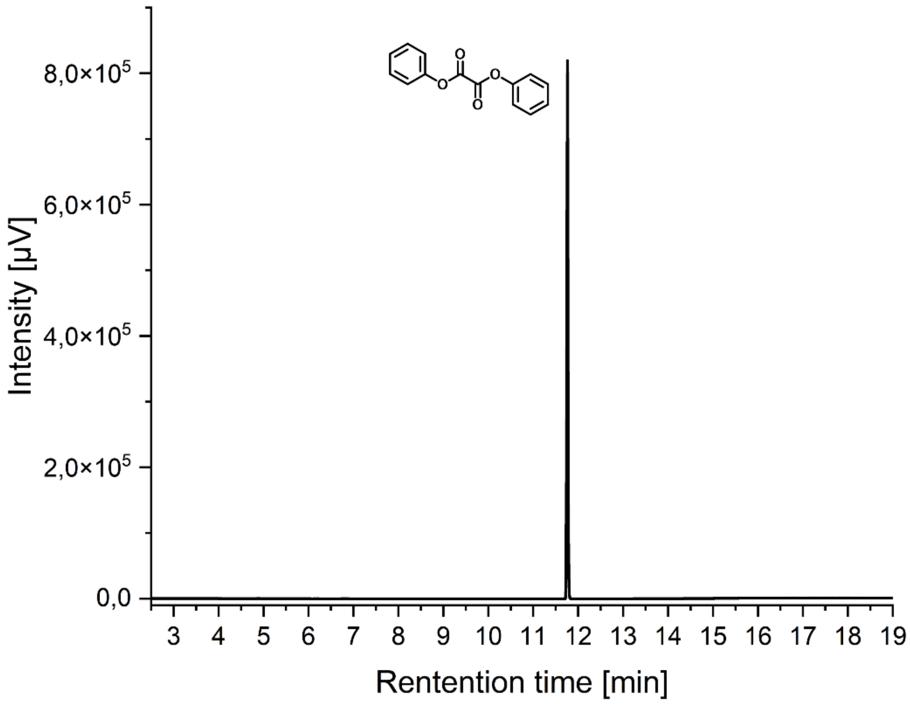

8,0×10^5
6,0×10^5
4,0×10^5
2,0×10^5
0,0
Intensity [µV]
3 4 5 6 7 8 9 10 11 12 13 14 15 16 17 18 19
Rentention time [min]

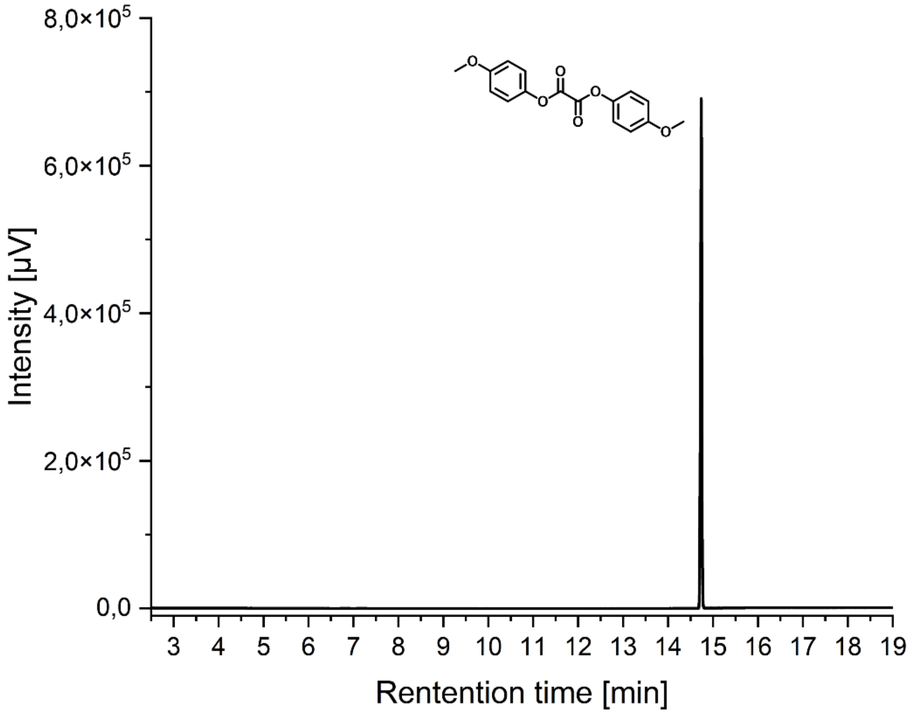
8,0×10^5
6,0×10^5
4,0×10^5
2,0×10^5
0,0
Intensity [µV]
3 4 5 6 7 8 9 10 11 12 13 14 15 16 17 18 19
Rentention time [min]

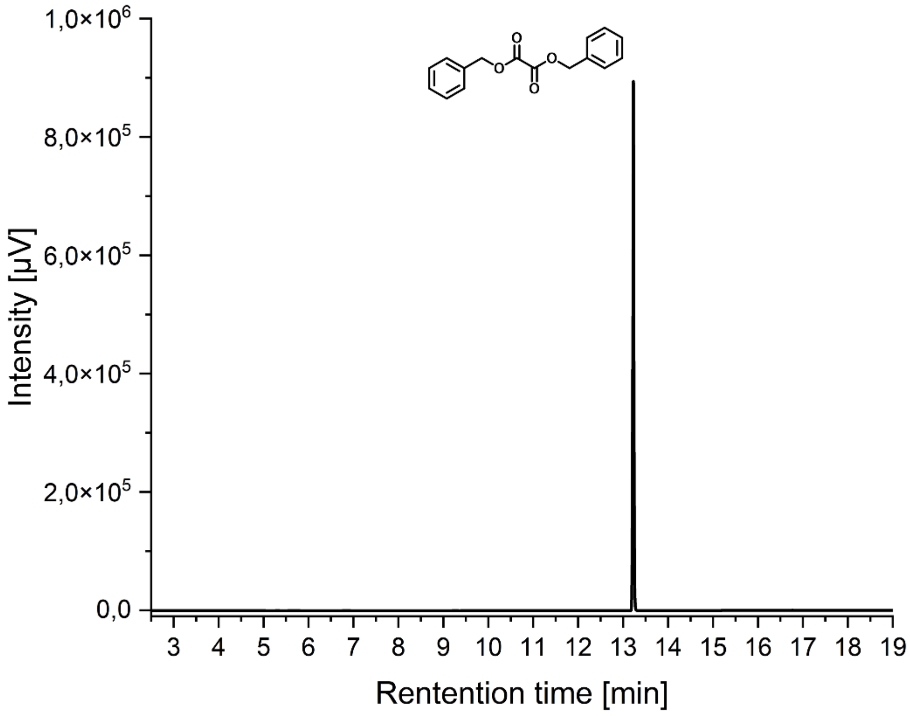
1,0×10^6
8,0×10^5
6,0×10^5
4,0×10^5
2,0×10^5
0,0
Intensity [µV]
3 4 5 6 7 8 9 10 11 12 13 14 15 16 17 18 19
Rentention time [min]

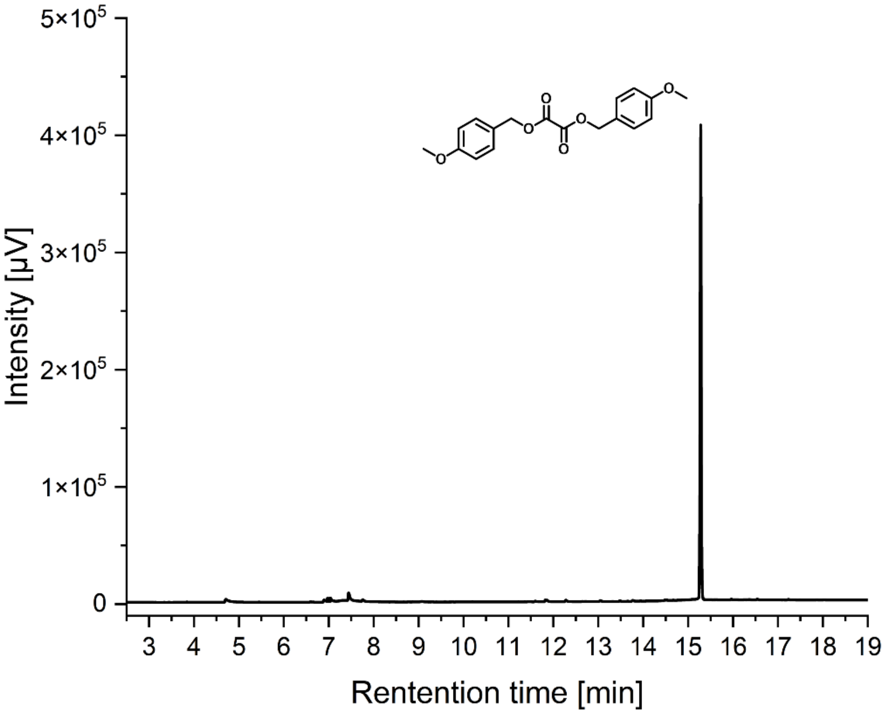

5×10^5
4×10^5
3×10^5
2×10^5
1×10^5
0
Intensity [µV]
3 4 5 6 7 8 9 10 11 12 13 14 15 16 17 18 19
Rentention time [min]

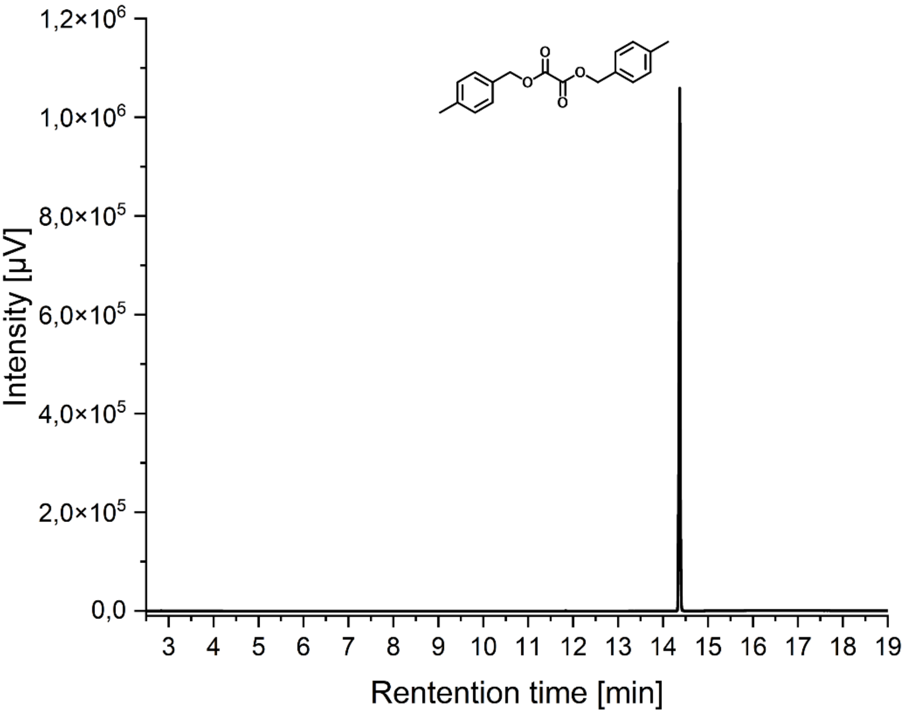

1,2×10^6
1,0×10^6
8,0×10^5
6,0×10^5
4,0×10^5
2,0×10^5
0,0
Intensity [µV]
3 4 5 6 7 8 9 10 11 12 13 14 15 16 17 18 19
Rentention time [min]

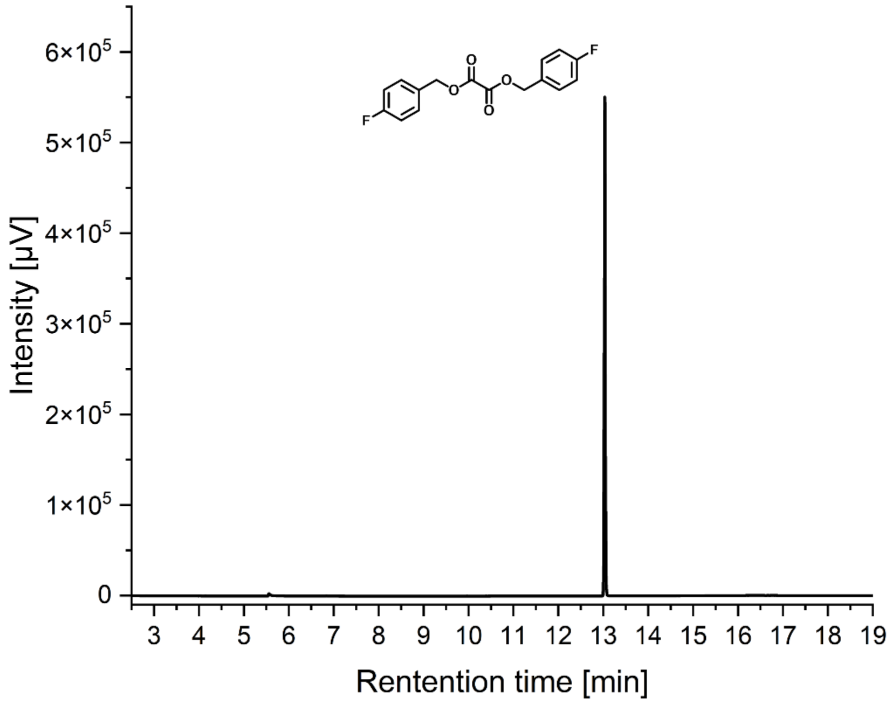
Intensity [µV]
6×10^5
5×10^5
4×10^5
3×10^5
2×10^5
1×10^5
0
3 4 5 6 7 8 9 10 11 12 13 14 15 16 17 18 19
Rentention time [min]
F
O
F

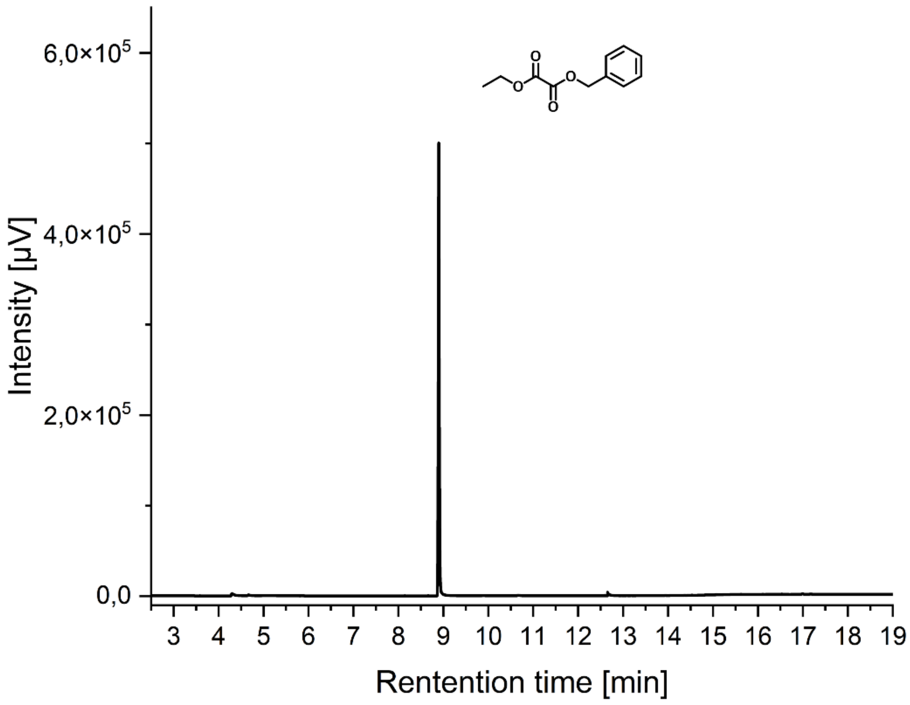
Intensity [µV]
6,0×10^5
4,0×10^5
2,0×10^5
0,0
3 4 5 6 7 8 9 10 11 12 13 14 15 16 17 18 19
Rentention time [min]
O

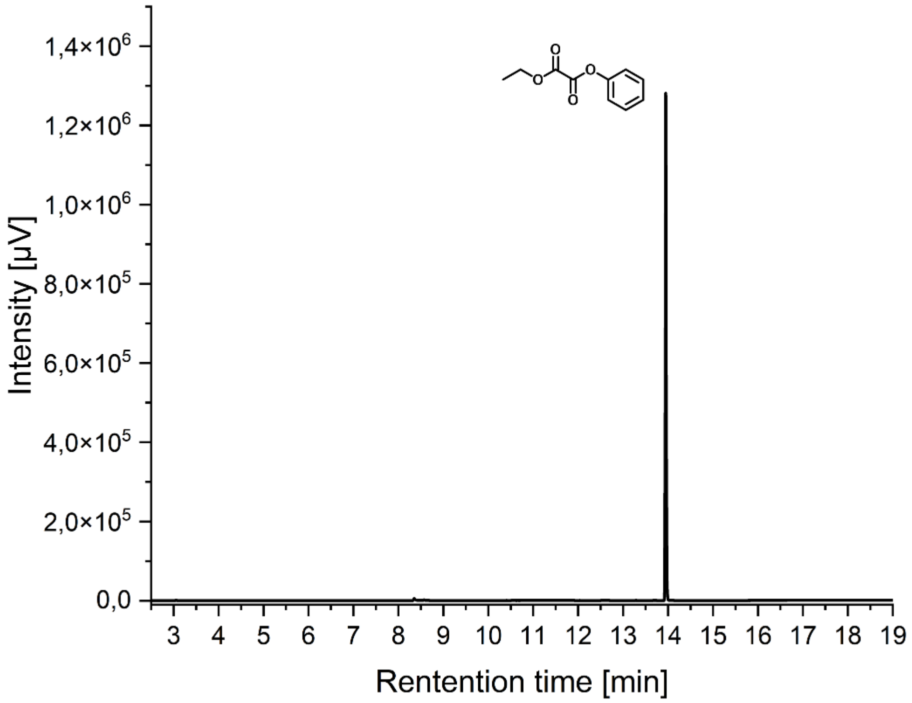

1,4×10^6
1,2×10^6
1,0×10^6
8,0×10^5
6,0×10^5
4,0×10^5
2,0×10^5
0,0
Intensity [µV]
3 4 5 6 7 8 9 10 11 12 13 14 15 16 17 18 19
Rentention time [min]

7. NMR spectra of the starting materials and the isolated products

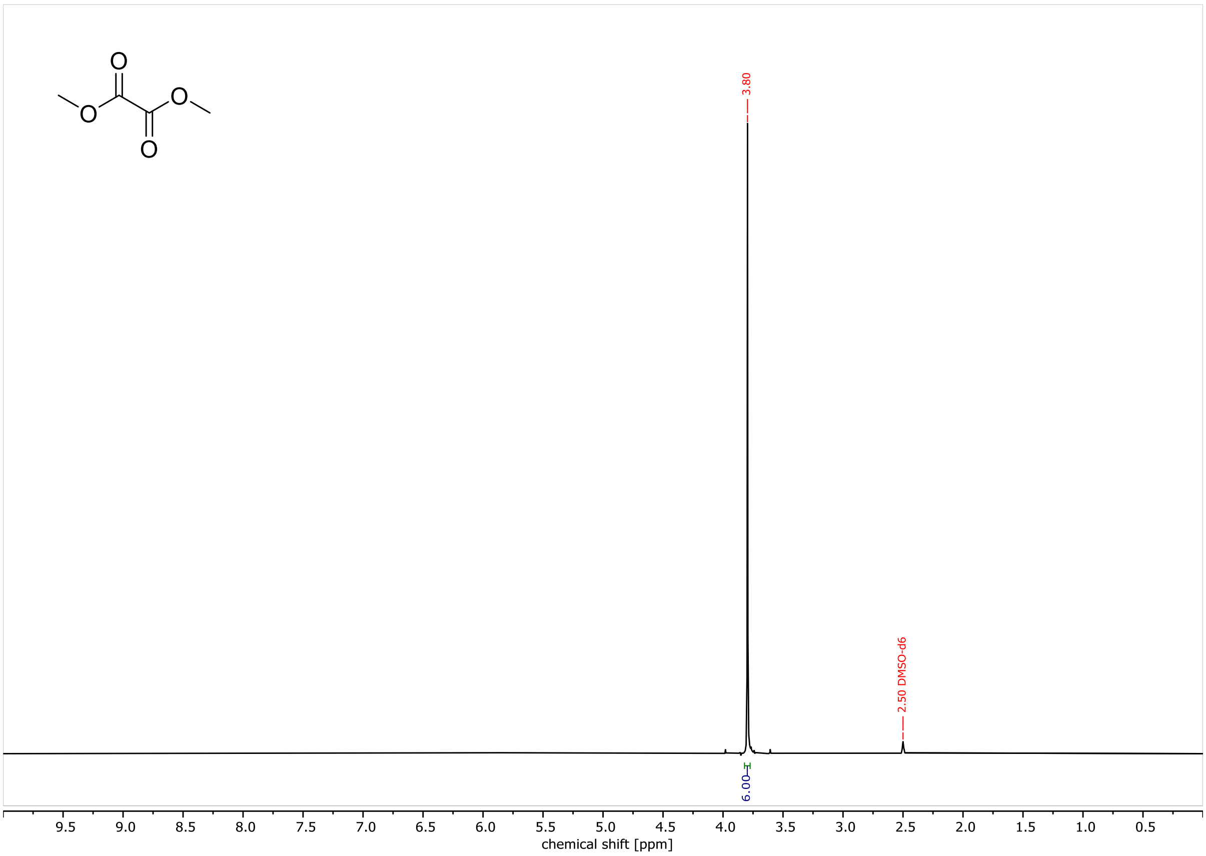

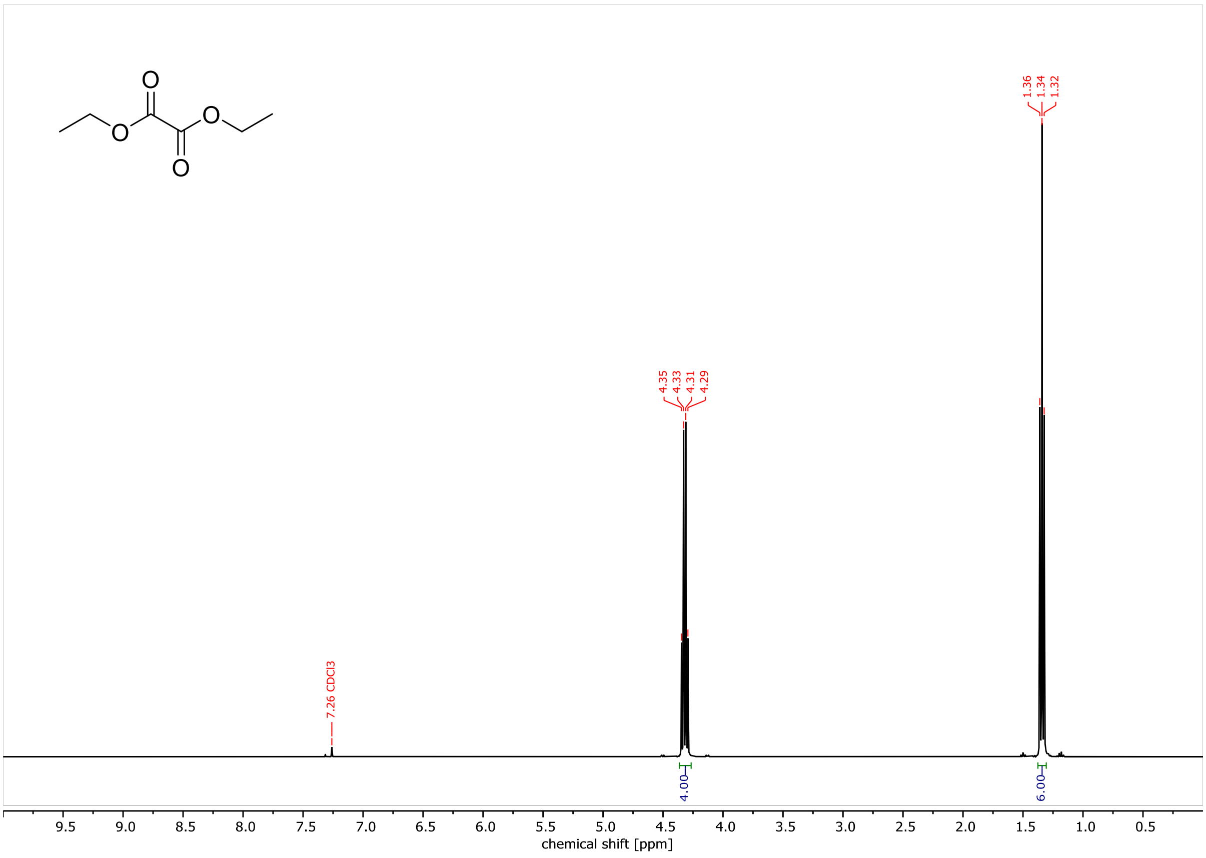

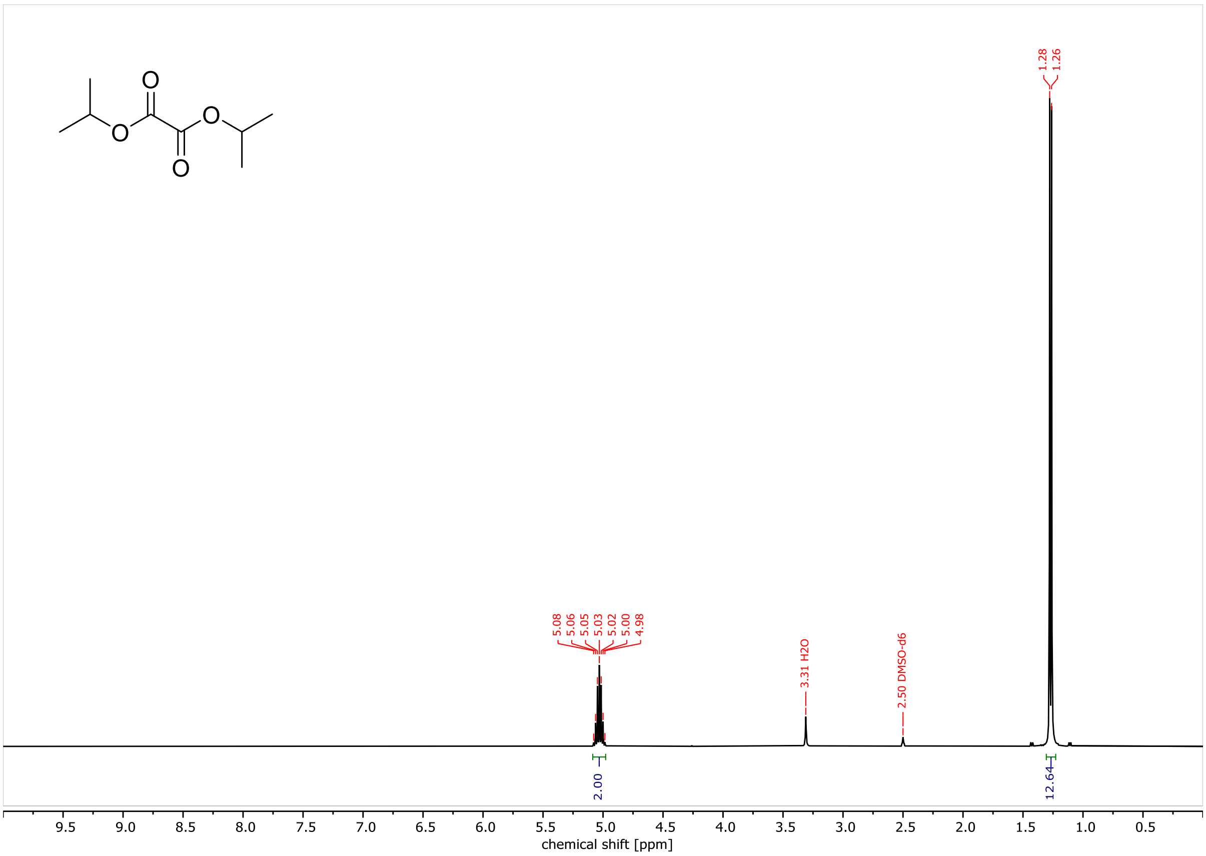
5.08
5.06
5.05
5.03
5.02
5.00
4.98
3.31 H2O
2.50 DMSO-d6
1.28
1.26
2.00
12.64
9.5 9.0 8.5 8.0 7.5 7.0 6.5 6.0 5.5 5.0 4.5 4.0 3.5 3.0 2.5 2.0 1.5 1.0 0.5
chemical shift [ppm]

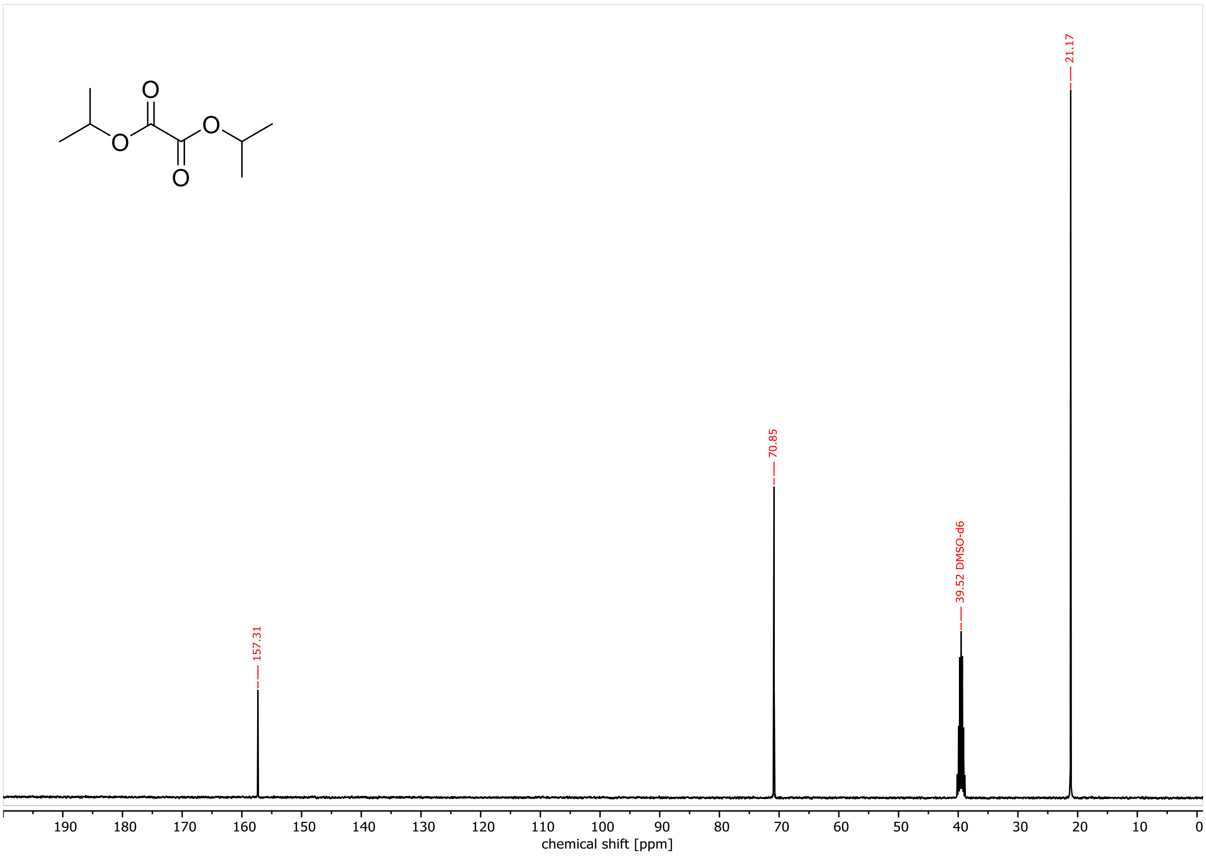
157.31
70.85
39.52 DMSO-d6
21.17
190 180 170 160 150 140 130 120 110 100 90 80 70 60 50 40 30 20 10 0
chemical shift [ppm]

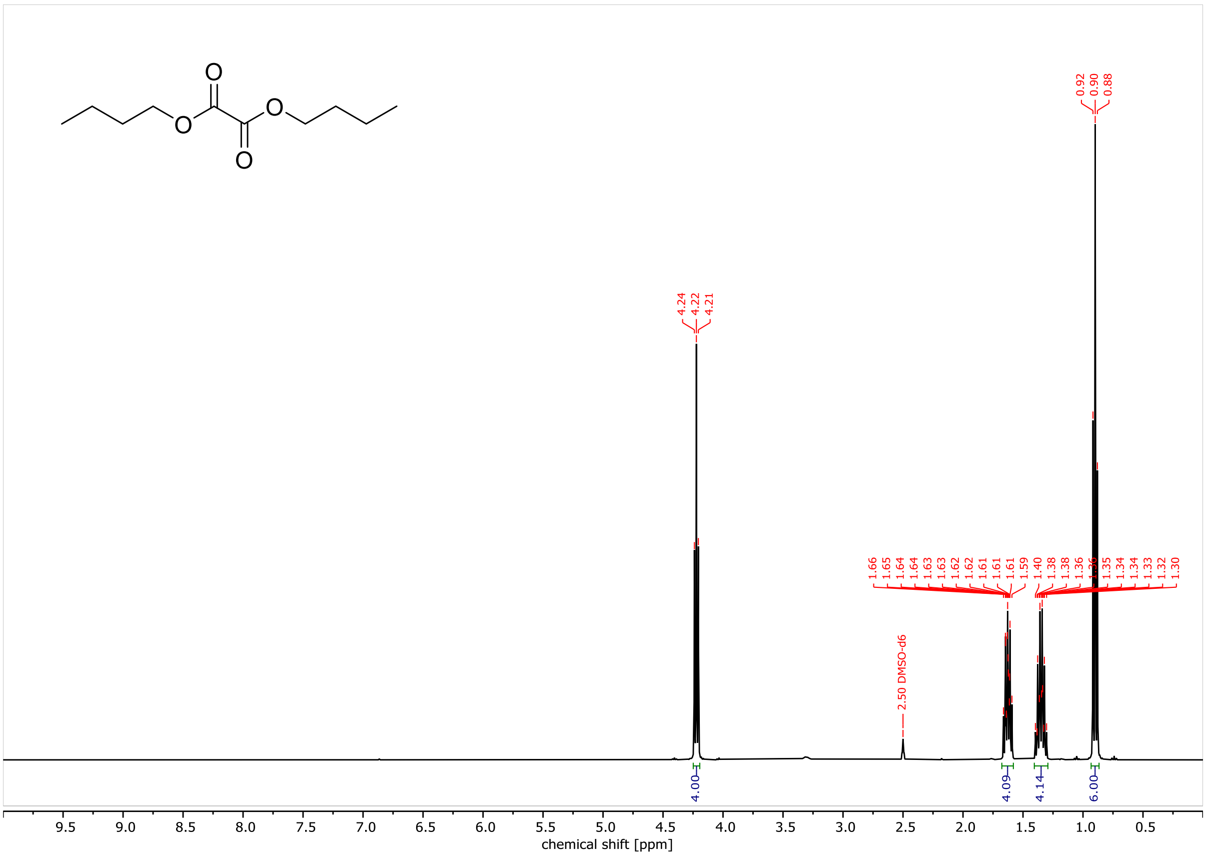
O
O
O
O
4.24
4.22
4.21
2.50 DMSO-d6
1.66
1.65
1.64
1.63
1.63
1.62
1.62
1.61
1.61
1.59
1.40
1.38
1.38
1.36
1.35
1.34
1.34
1.33
1.32
1.30
0.92
0.90
0.88
4.00
4.09
4.14
6.00
chemical shift [ppm]

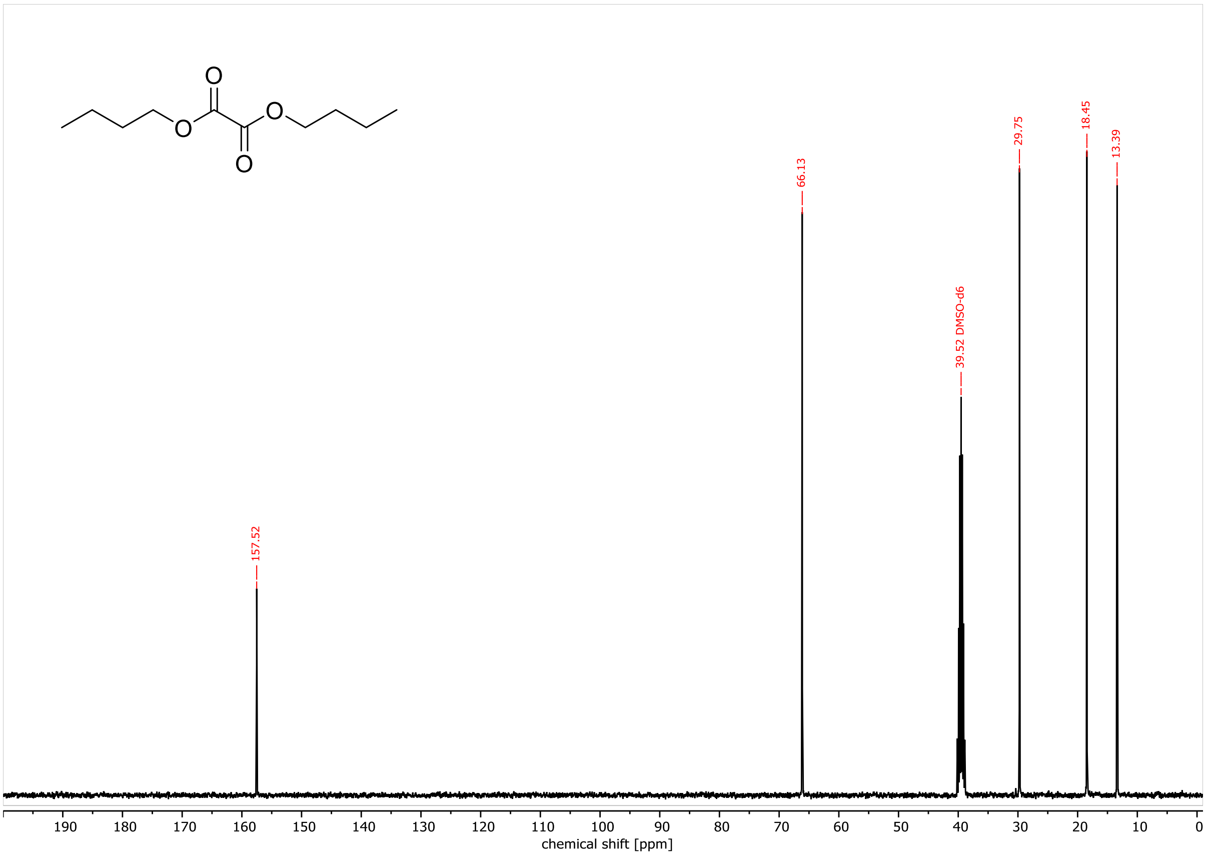
O
O
O
O
157.52
66.13
39.52 DMSO-d6
29.75
18.45
13.39
chemical shift [ppm]

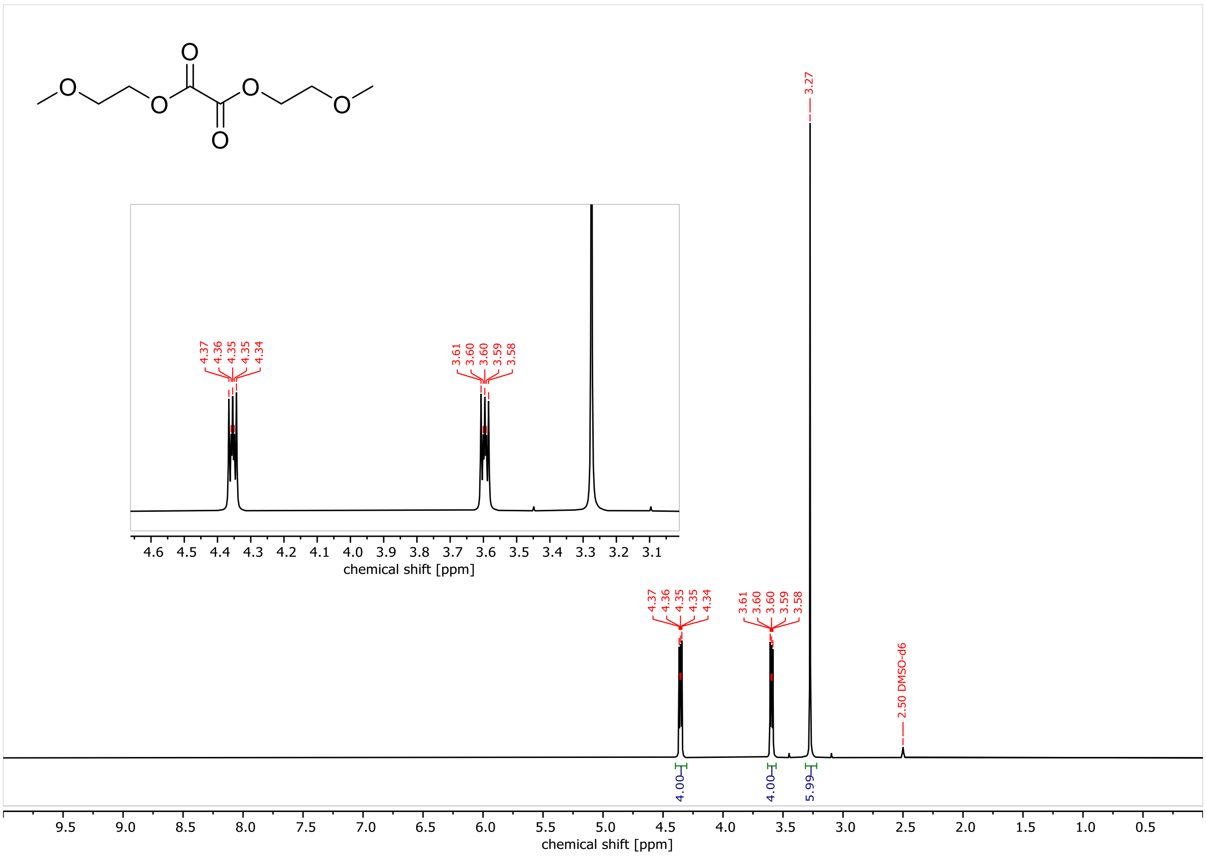
O
O
O
O
O
O
3.27
4.37
4.36
4.35
4.35
4.34
3.61
3.60
3.60
3.59
3.58
4.6 4.5 4.4 4.3 4.2 4.1 4.0 3.9 3.8 3.7 3.6 3.5 3.4 3.3 3.2 3.1
chemical shift [ppm]
2.50 DMSO-d6
4.00
4.00
5.99
9.5 9.0 8.5 8.0 7.5 7.0 6.5 6.0 5.5 5.0 4.5 4.0 3.5 3.0 2.5 2.0 1.5 1.0 0.5
chemical shift [ppm]

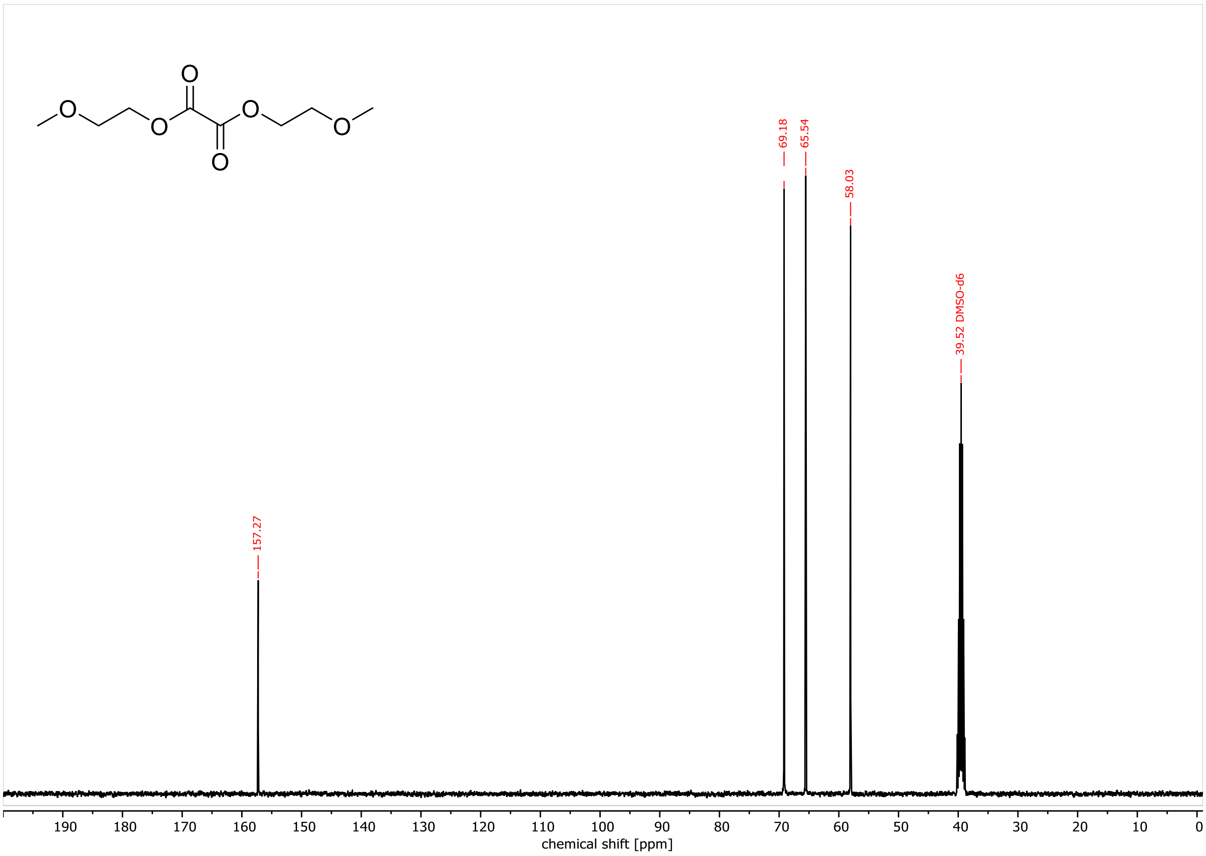
O
O
O
O
O
O
69.18
65.54
58.03
39.52 DMSO-d6
157.27
190 180 170 160 150 140 130 120 110 100 90 80 70 60 50 40 30 20 10 0
chemical shift [ppm]

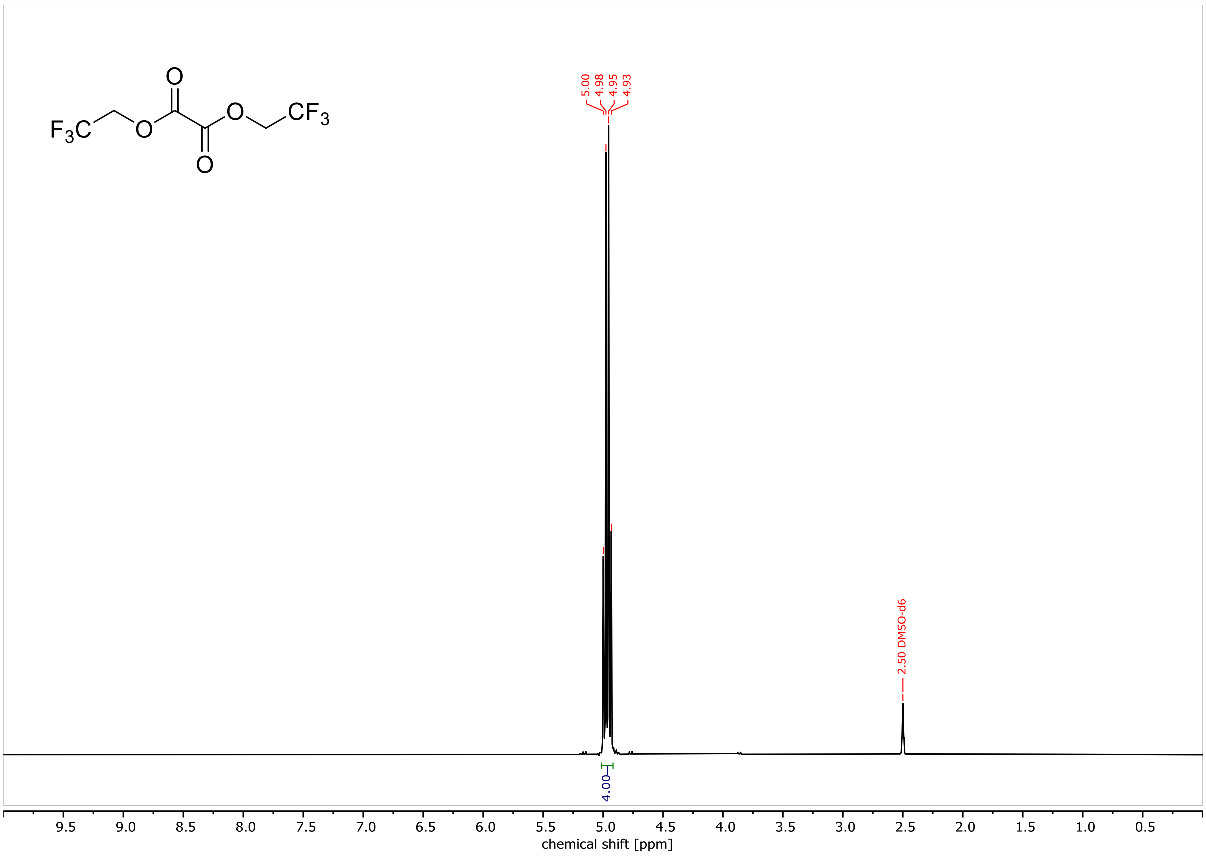
F3C
O
O
O
O
CF3
5.00
4.98
4.95
4.93
2.50 DMSO-d6
4.00
9.5
9.0
8.5
8.0
7.5
7.0
6.5
6.0
5.5
5.0
4.5
4.0
3.5
3.0
2.5
2.0
1.5
1.0
0.5
chemical shift [ppm]

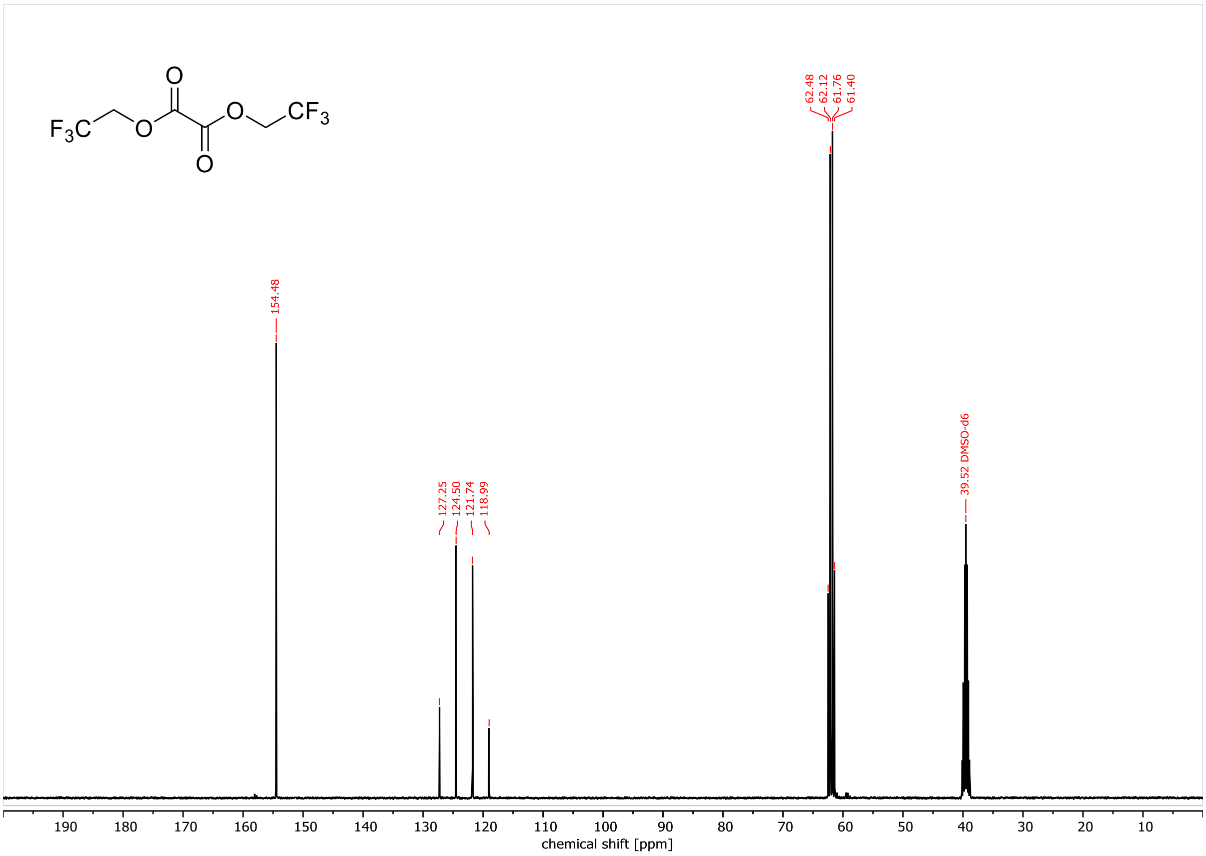
F3C
O
O
O
O
CF3
154.48
127.25
124.50
121.74
118.99
62.48
62.12
61.76
61.40
39.52 DMSO-d6
190
180
170
160
150
140
130
120
110
100
90
80
70
60
50
40
30
20
10
chemical shift [ppm]

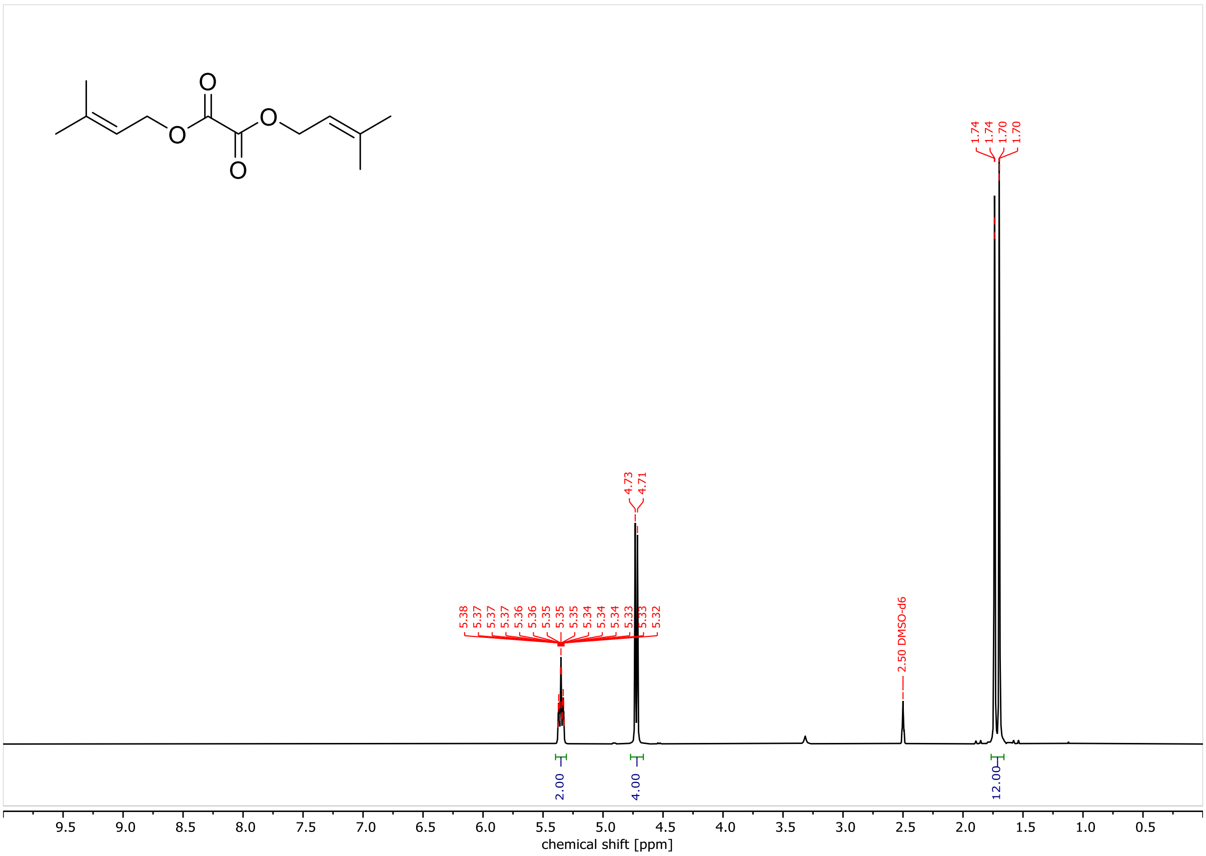
5.38
5.37
5.37
5.37
5.36
5.36
5.35
5.35
5.35
5.34
5.34
5.34
5.33
5.33
5.33
5.32
4.73
4.71
2.50 DMSO-d6
1.74
1.74
1.70
1.70
2.00
4.00
12.00
9.5 9.0 8.5 8.0 7.5 7.0 6.5 6.0 5.5 5.0 4.5 4.0 3.5 3.0 2.5 2.0 1.5 1.0 0.5
chemical shift [ppm]

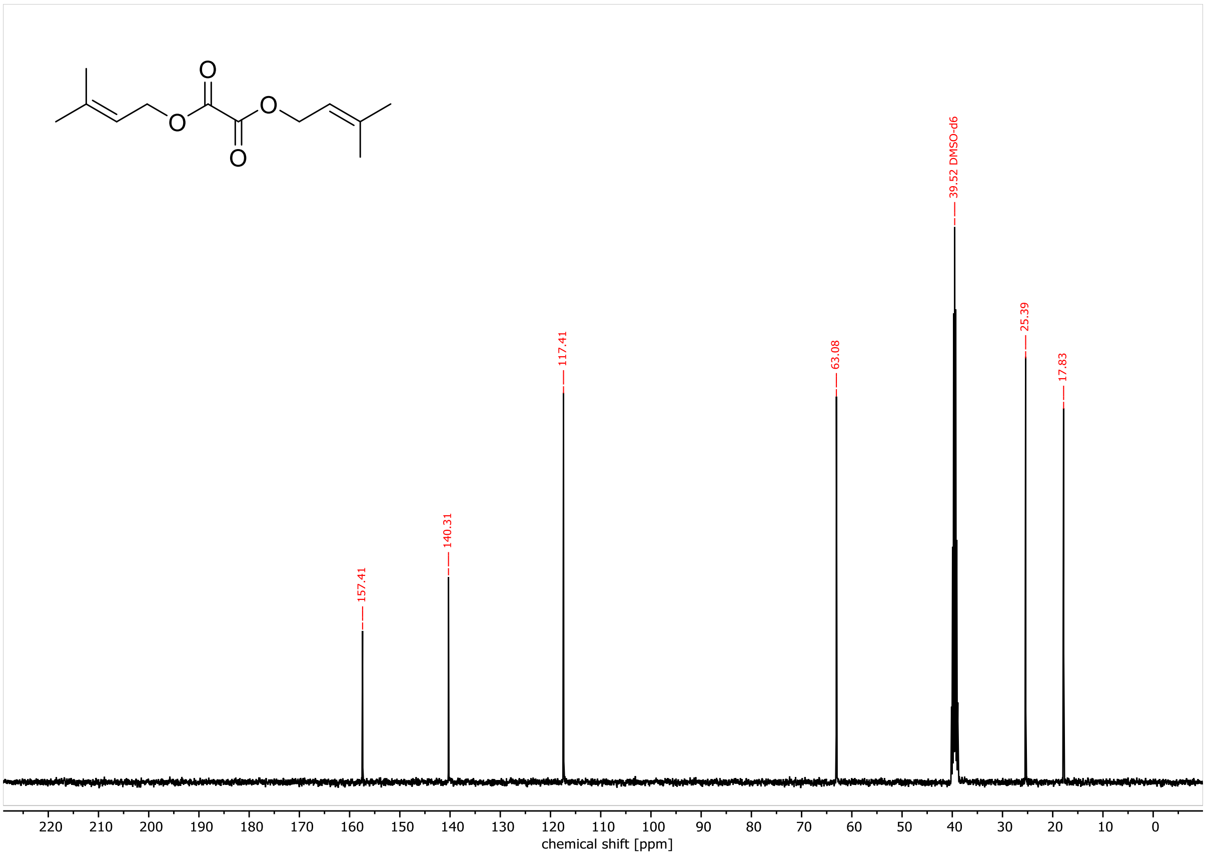
157.41
140.31
117.41
63.08
39.52 DMSO-d6
25.39
17.83
220 210 200 190 180 170 160 150 140 130 120 110 100 90 80 70 60 50 40 30 20 10 0
chemical shift [ppm]

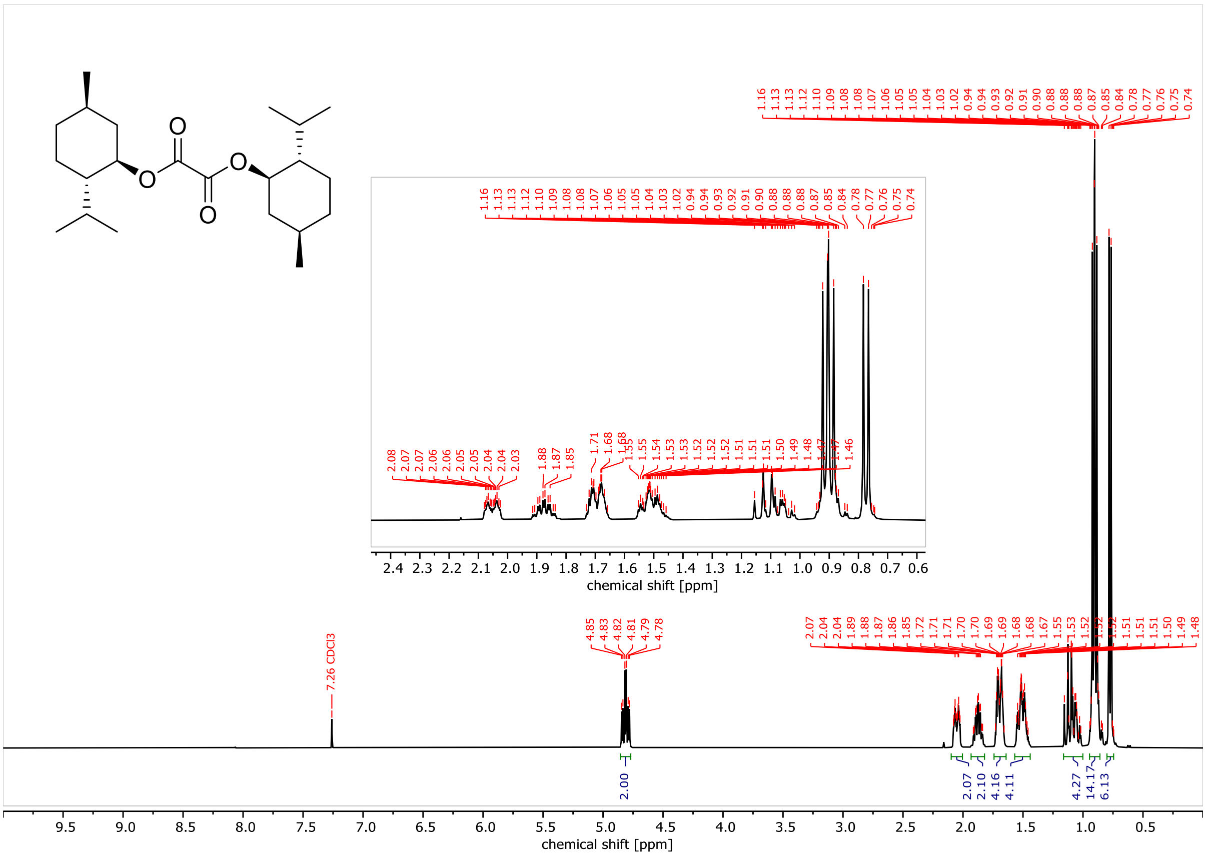

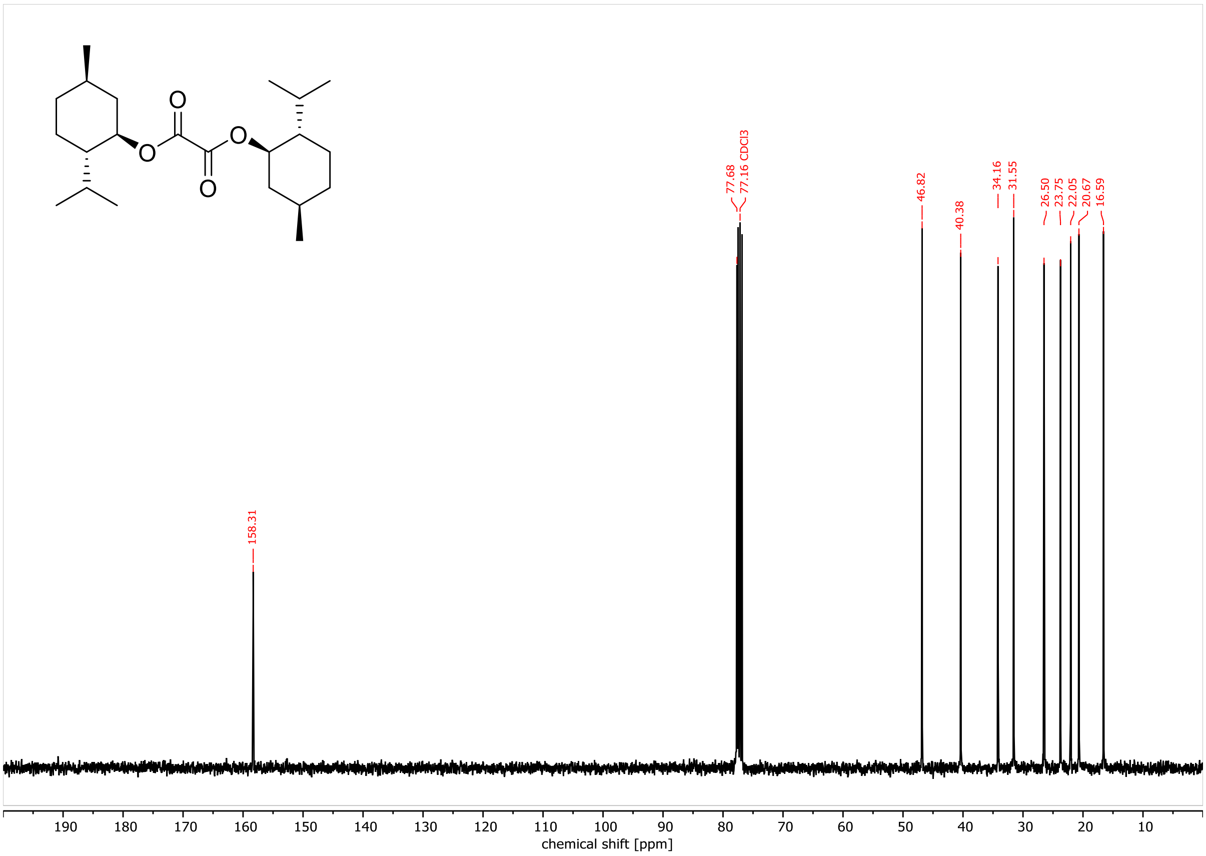

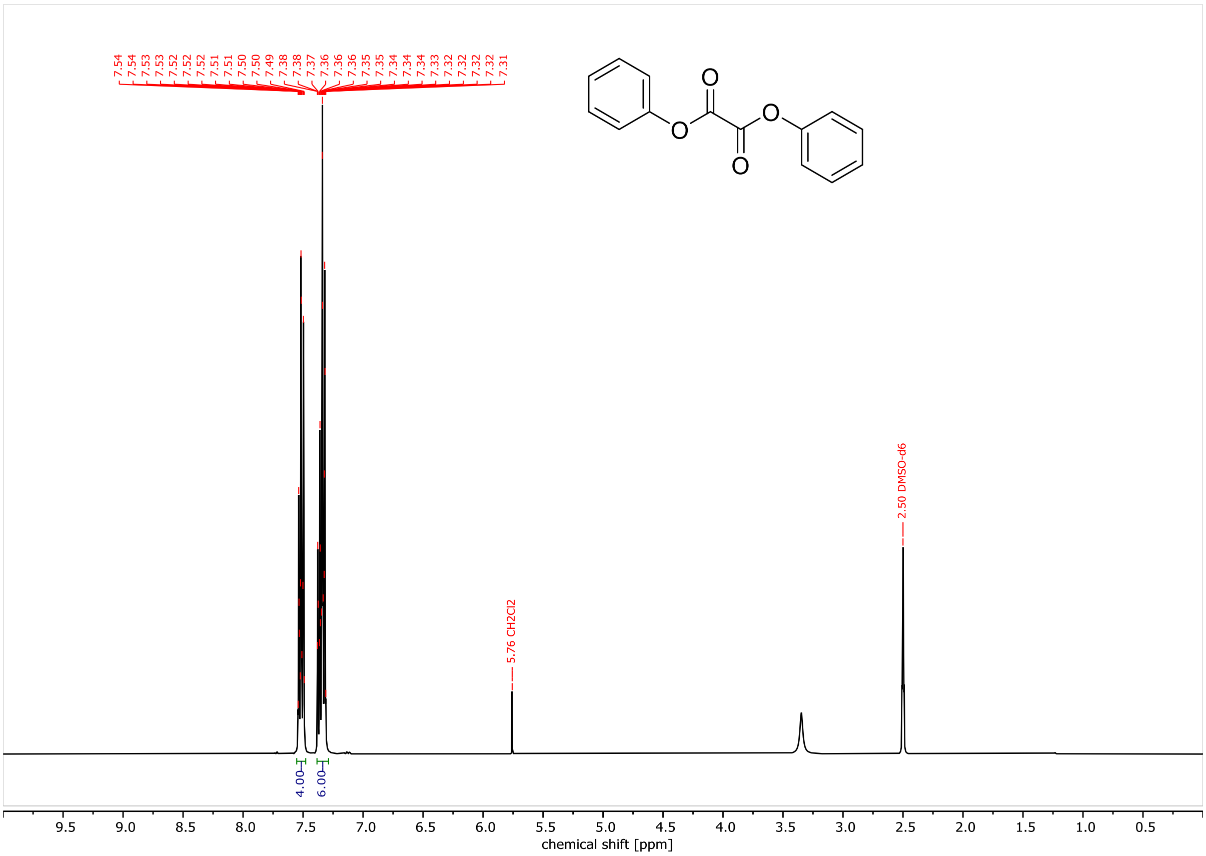
7.54
7.54
7.53
7.53
7.52
7.52
7.52
7.51
7.51
7.50
7.50
7.49
7.38
7.38
7.37
7.36
7.36
7.36
7.35
7.35
7.34
7.34
7.34
7.33
7.32
7.32
7.32
7.32
7.31
O
O
O
O
5.76 CH2Cl2
2.50 DMSO-d6
4.00
6.00
9.5
9.0
8.5
8.0
7.5
7.0
6.5
6.0
5.5
5.0
4.5
4.0
3.5
3.0
2.5
2.0
1.5
1.0
0.5
chemical shift [ppm]

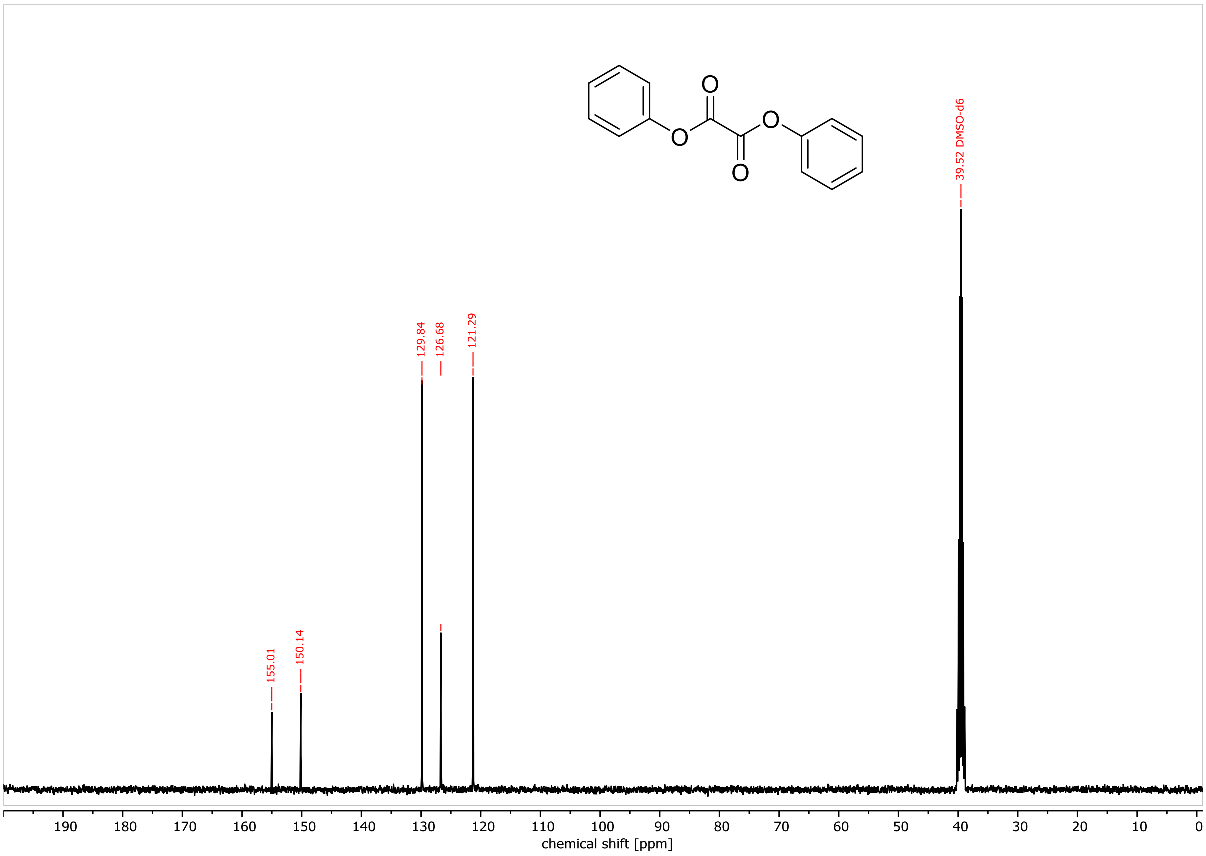
O
O
O
O
39.52 DMSO-d6
129.84
126.68
121.29
155.01
150.14
190
180
170
160
150
140
130
120
110
100
90
80
70
60
50
40
30
20
10
0
chemical shift [ppm]

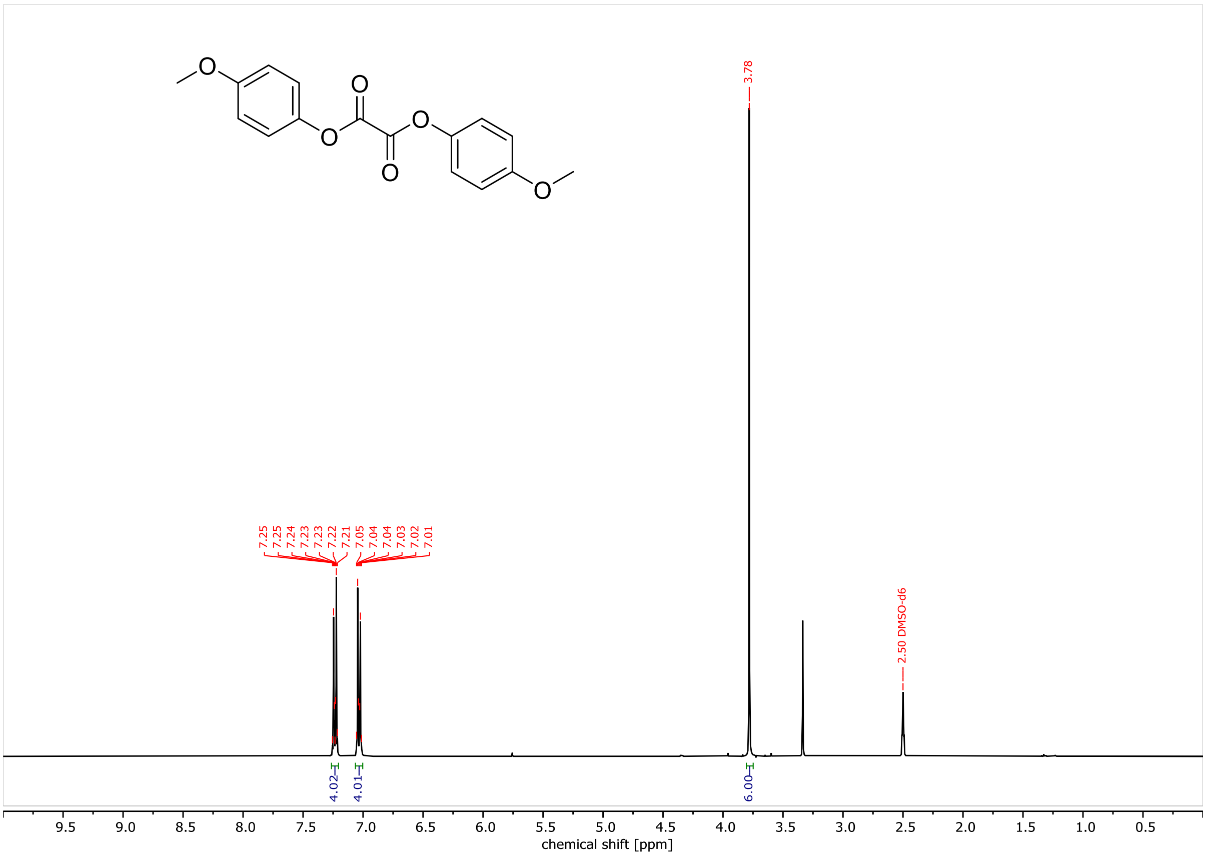

7.25
7.25
7.24
7.23
7.23
7.22
7.21
7.05
7.04
7.04
7.03
7.02
7.01
3.78
2.50 DMSO-d6
4.02
4.01
6.00
chemical shift [ppm]

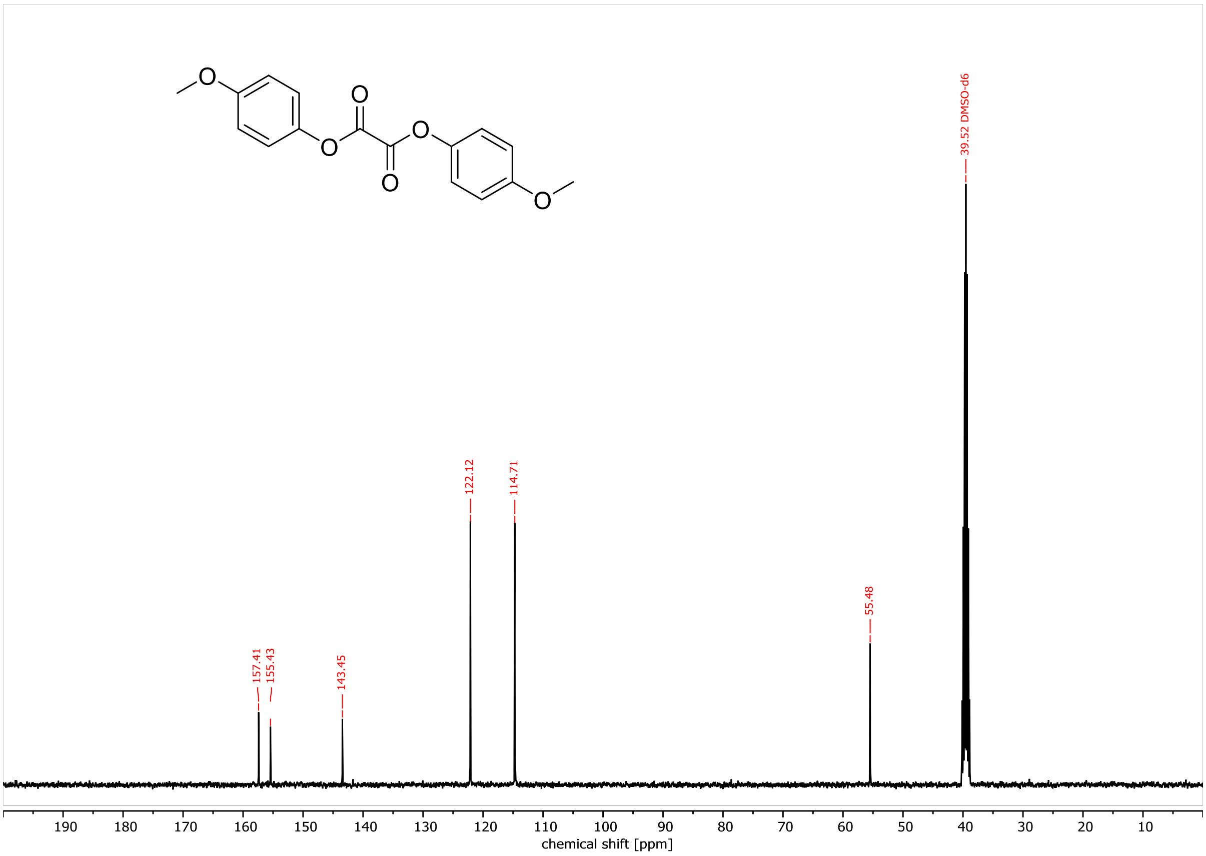

157.41
155.43
143.45
122.12
114.71
55.48
39.52 DMSO-d6
chemical shift [ppm]

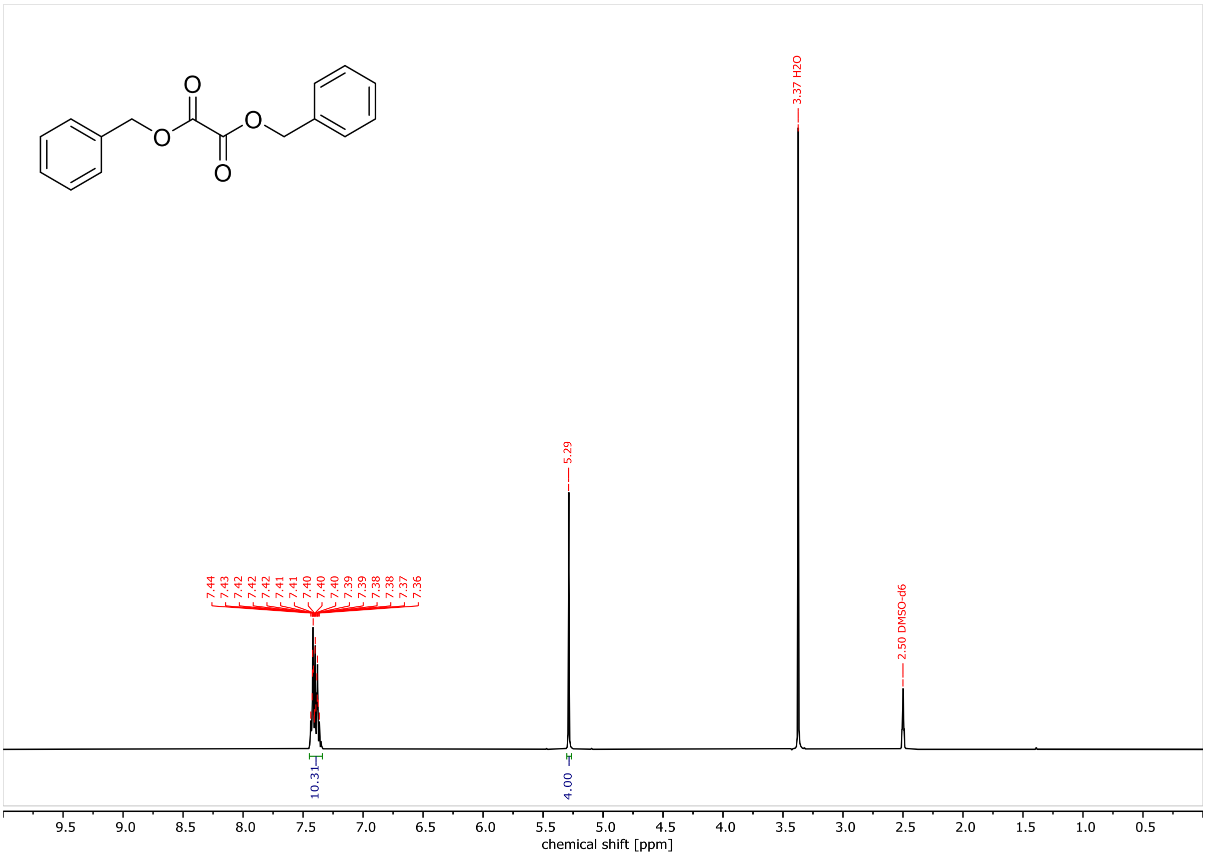

O
O
O
O
7.44
7.43
7.42
7.42
7.42
7.41
7.41
7.40
7.40
7.40
7.39
7.39
7.38
7.38
7.37
7.36
5.29
3.37 H2O
2.50 DMSO-d6
10.31
4.00
9.5 9.0 8.5 8.0 7.5 7.0 6.5 6.0 5.5 5.0 4.5 4.0 3.5 3.0 2.5 2.0 1.5 1.0 0.5
chemical shift [ppm]

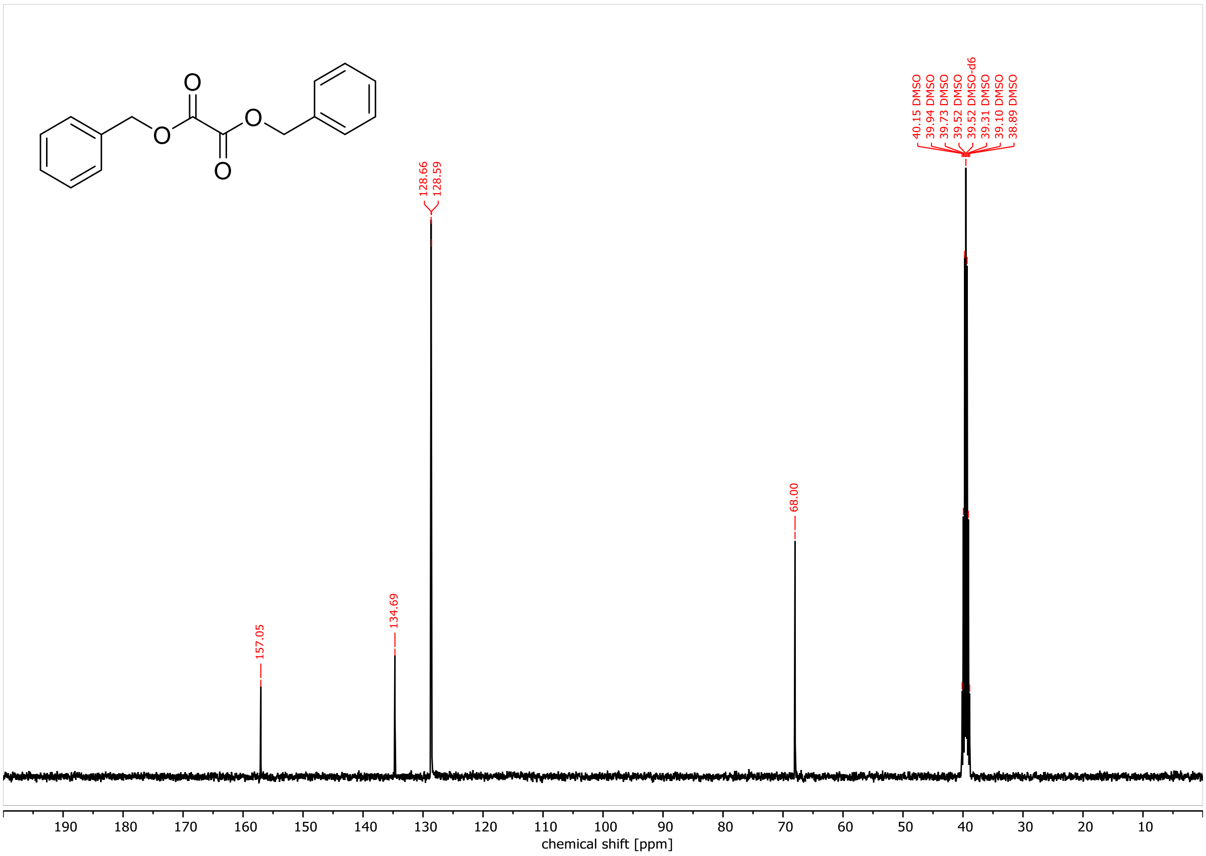

O
O
O
O
157.05
134.69
128.66
128.59
68.00
40.15 DMSO
39.94 DMSO
39.73 DMSO
39.52 DMSO-d6
39.31 DMSO
39.10 DMSO
38.89 DMSO
190 180 170 160 150 140 130 120 110 100 90 80 70 60 50 40 30 20 10
chemical shift [ppm]

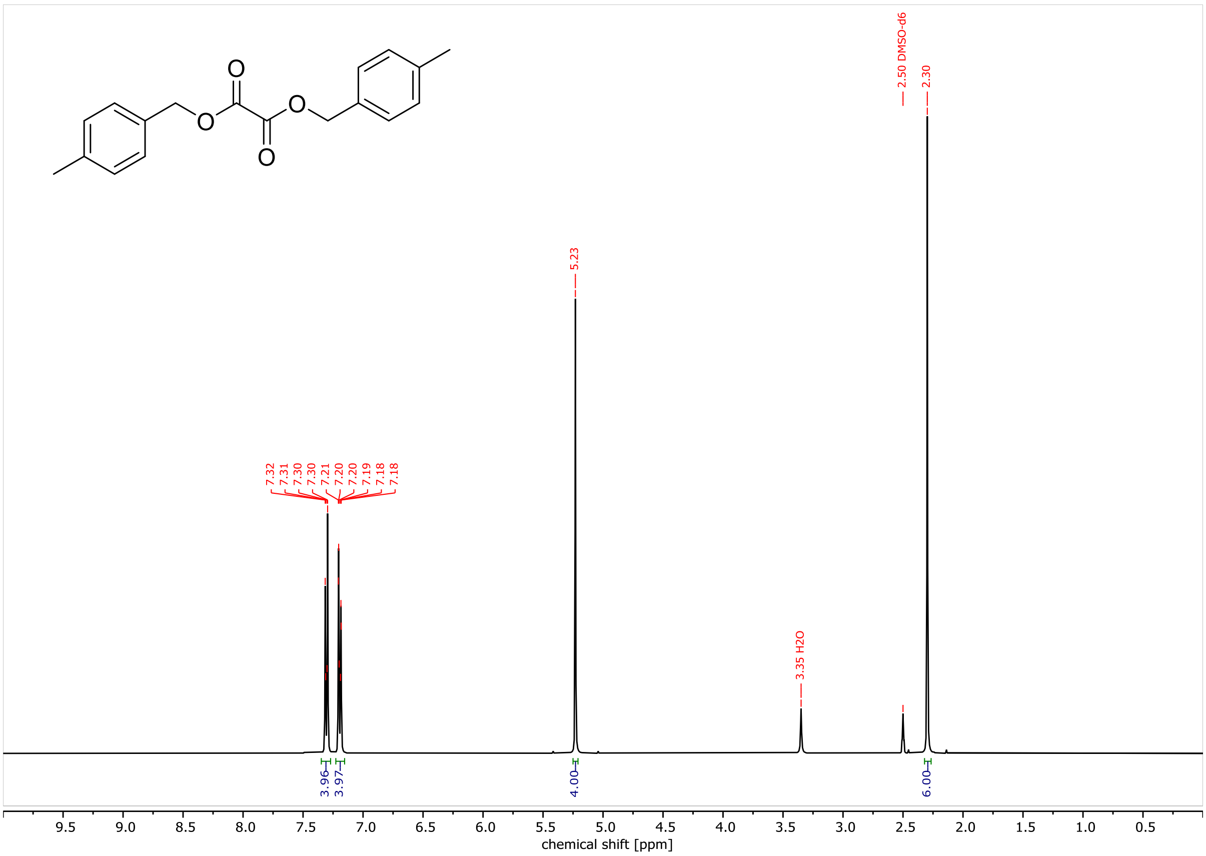
2.50 DMSO-d6
2.30
5.23
7.32
7.31
7.30
7.30
7.21
7.20
7.20
7.19
7.18
7.18
3.35 H2O
3.96
3.97
4.00
6.00
9.5 9.0 8.5 8.0 7.5 7.0 6.5 6.0 5.5 5.0 4.5 4.0 3.5 3.0 2.5 2.0 1.5 1.0 0.5
chemical shift [ppm]

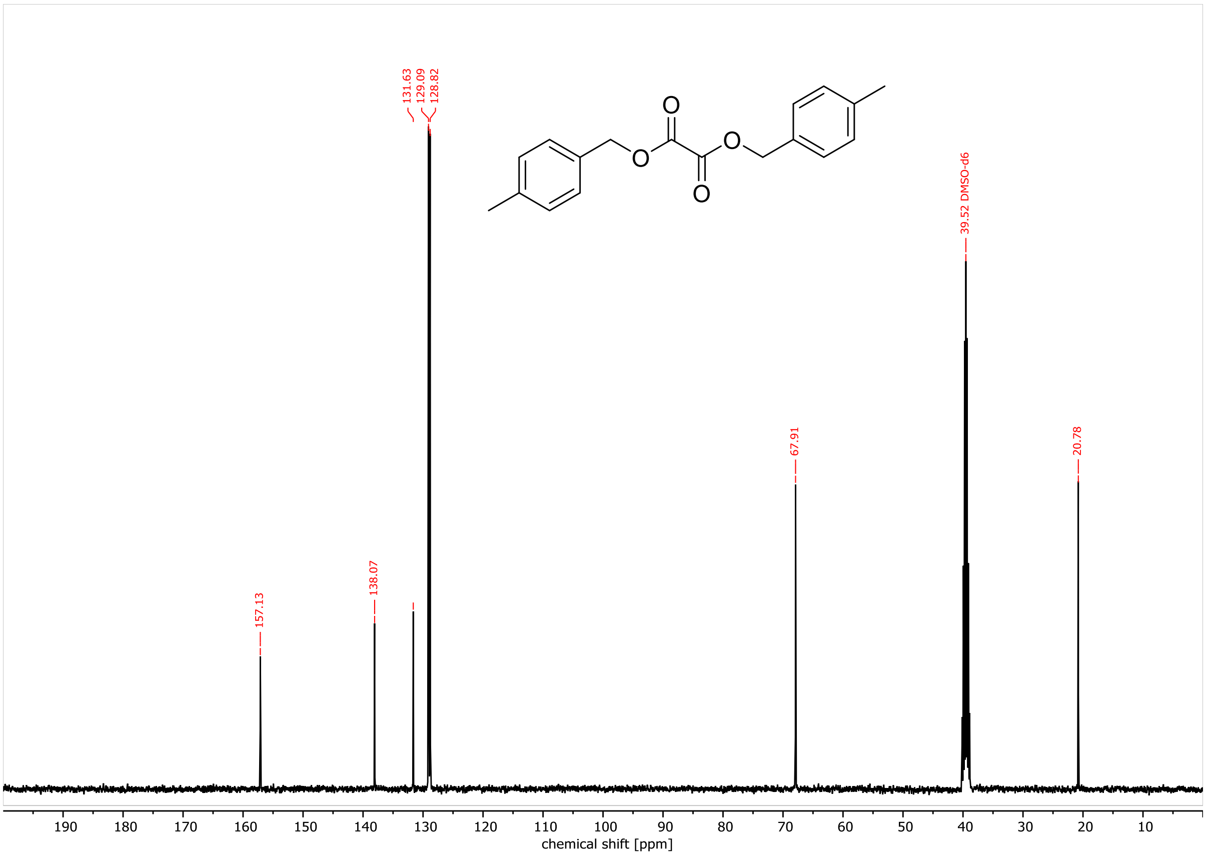
131.63
129.09
128.82
39.52 DMSO-d6
67.91
20.78
157.13
138.07
190 180 170 160 150 140 130 120 110 100 90 80 70 60 50 40 30 20 10
chemical shift [ppm]

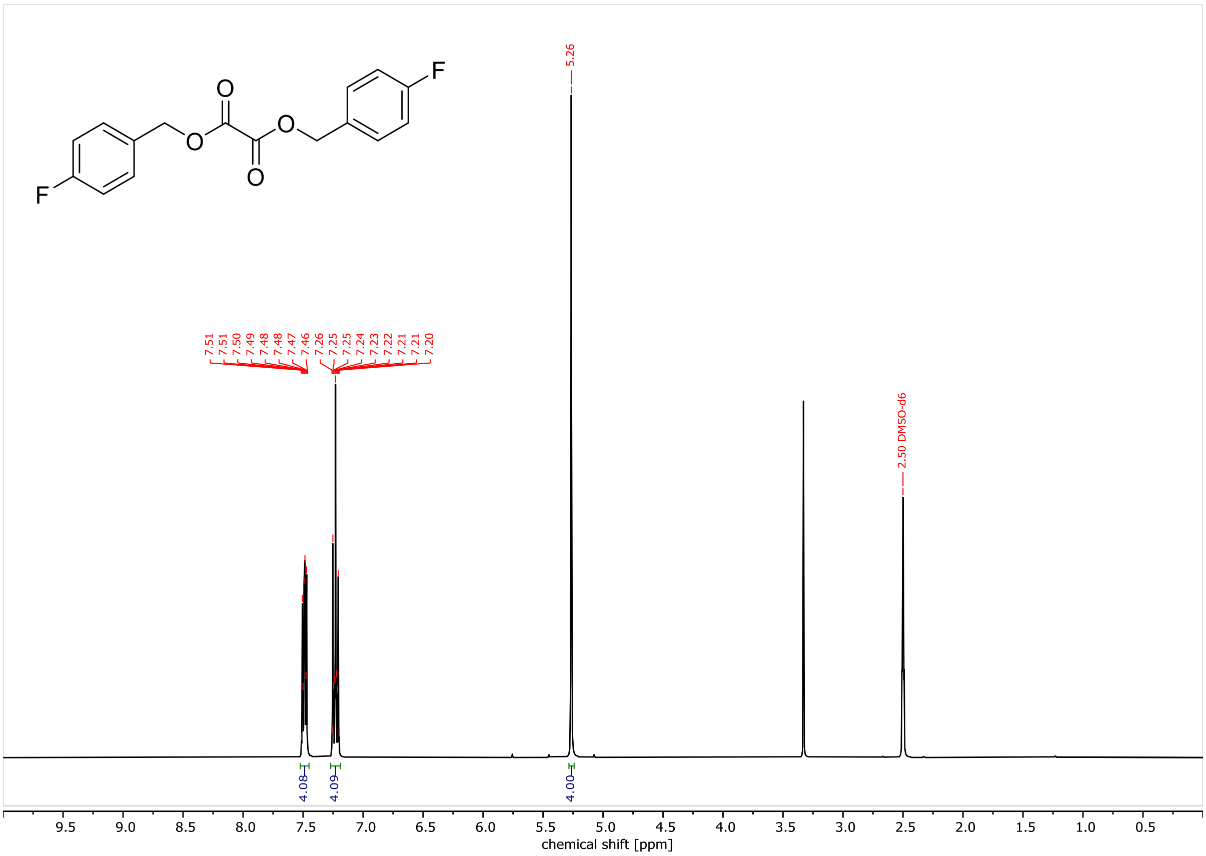

5.26
7.51
7.51
7.50
7.49
7.48
7.48
7.47
7.46
7.26
7.25
7.25
7.24
7.23
7.22
7.21
7.21
7.20
2.50 DMSO-d6
4.08
4.09
4.00
9.5 9.0 8.5 8.0 7.5 7.0 6.5 6.0 5.5 5.0 4.5 4.0 3.5 3.0 2.5 2.0 1.5 1.0 0.5
chemical shift [ppm]

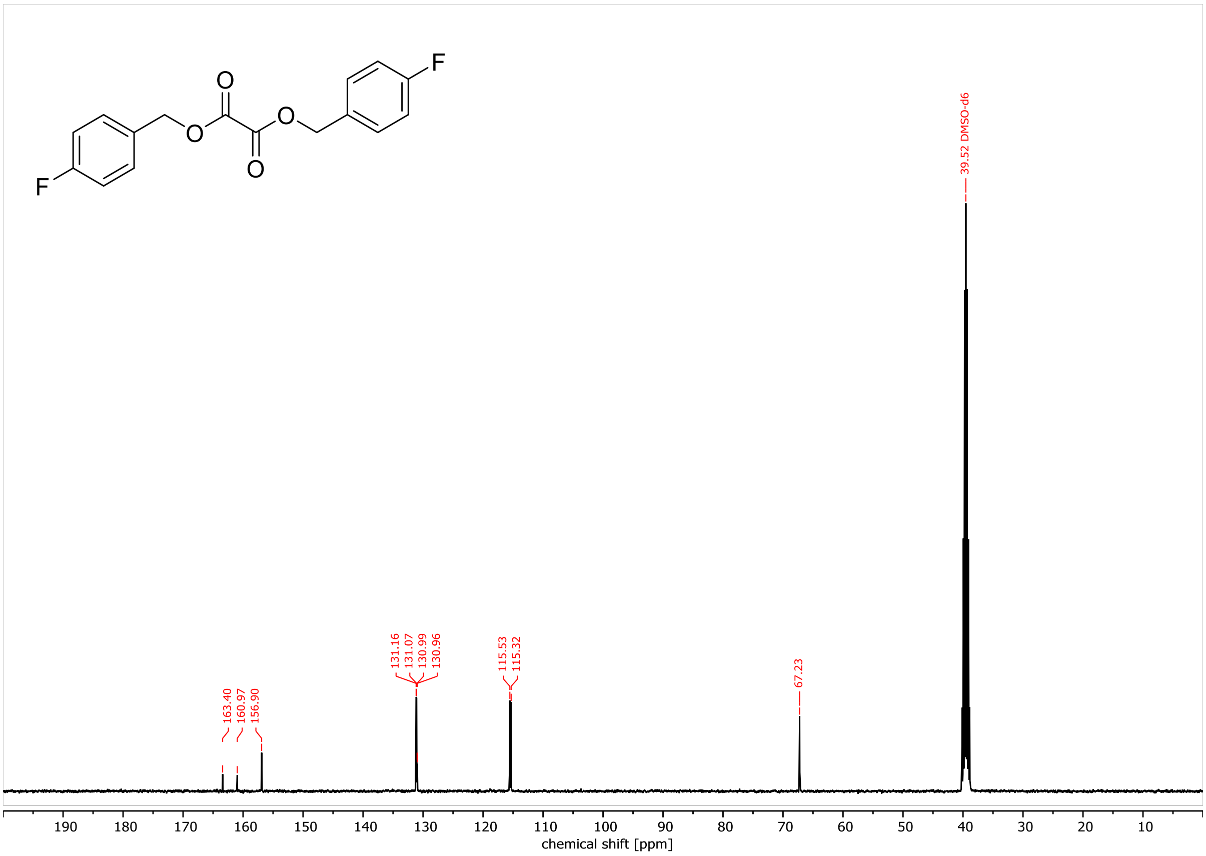

39.52 DMSO-d6
163.40
160.97
156.90
131.16
131.07
130.99
130.96
115.53
115.32
67.23
190 180 170 160 150 140 130 120 110 100 90 80 70 60 50 40 30 20 10
chemical shift [ppm]

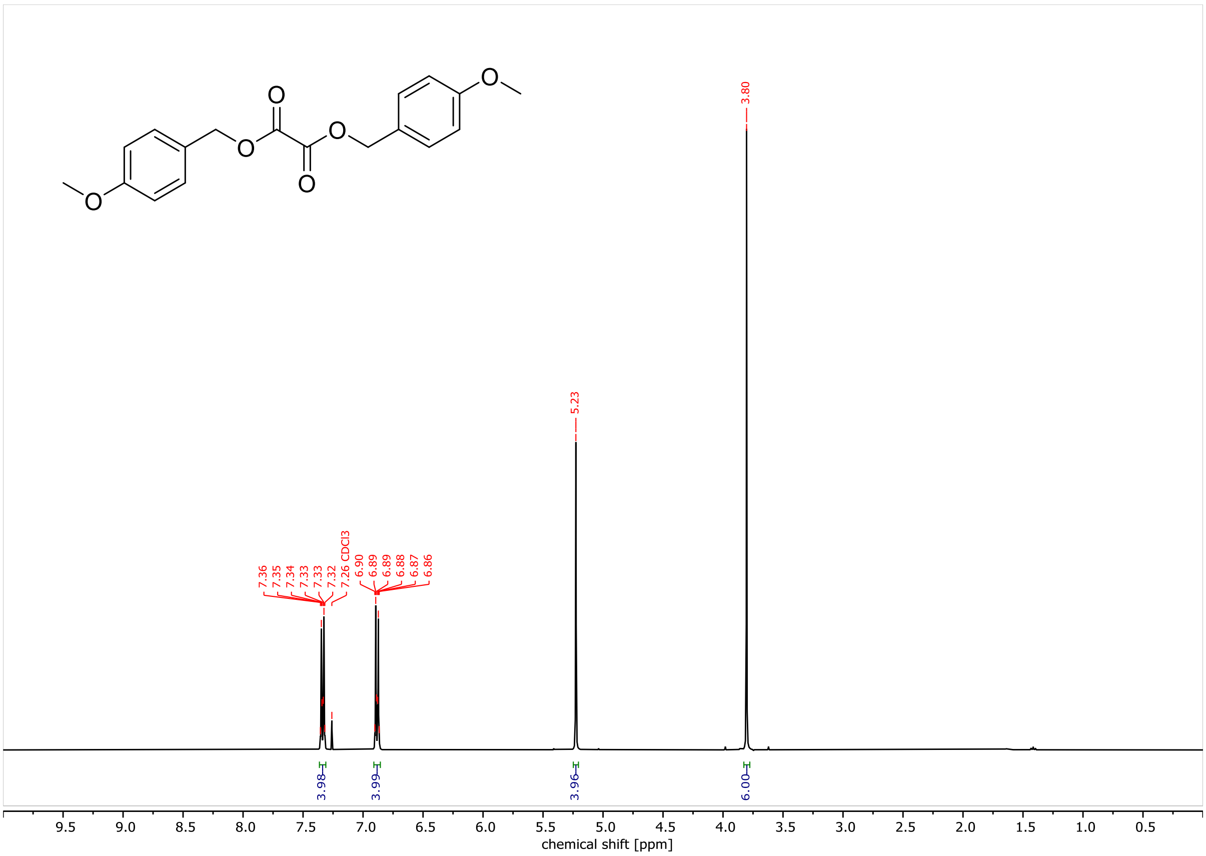
3.80
5.23
7.36
7.35
7.34
7.33
7.33
7.32
7.26 CDCl3
6.90
6.89
6.89
6.88
6.87
6.86
3.98
3.99
3.96
6.00
9.5 9.0 8.5 8.0 7.5 7.0 6.5 6.0 5.5 5.0 4.5 4.0 3.5 3.0 2.5 2.0 1.5 1.0 0.5
chemical shift [ppm]

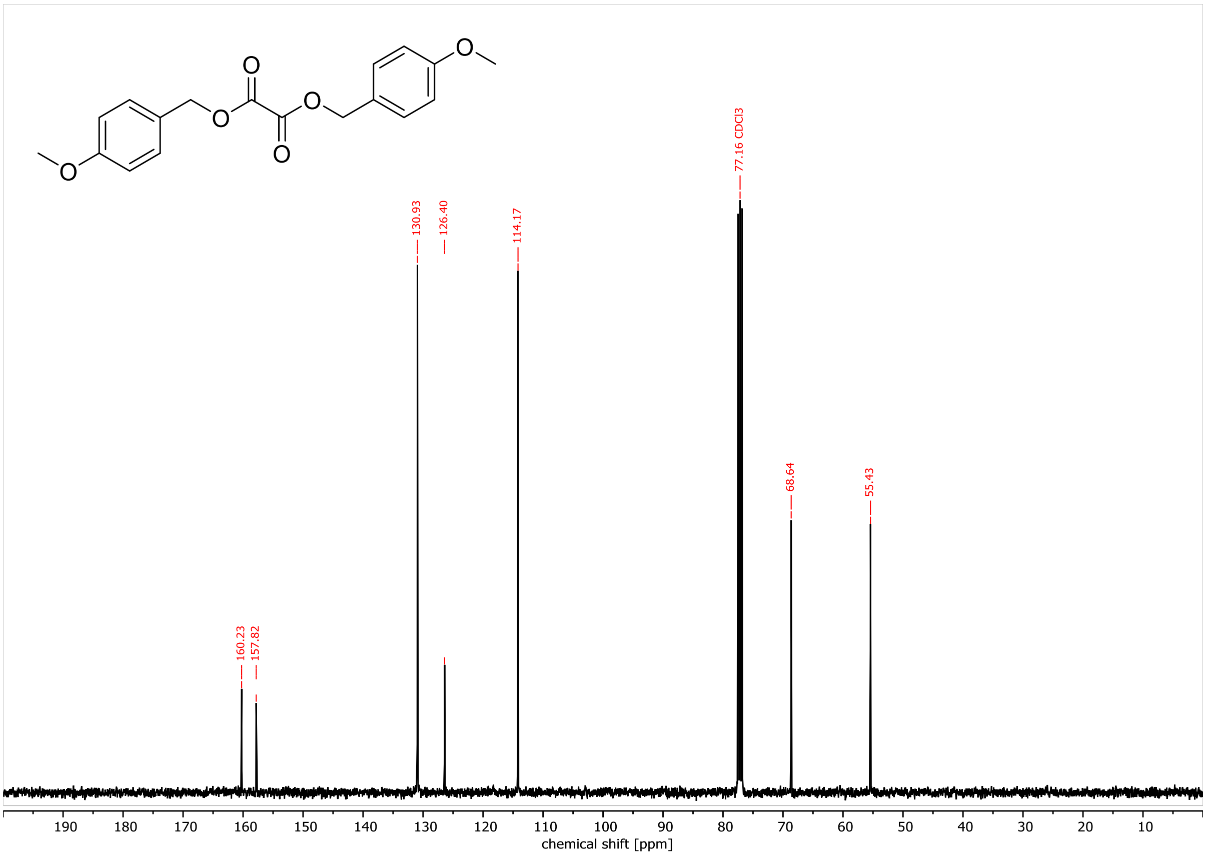
77.16 CDCl3
130.93
126.40
114.17
68.64
55.43
160.23
157.82
190 180 170 160 150 140 130 120 110 100 90 80 70 60 50 40 30 20 10
chemical shift [ppm]

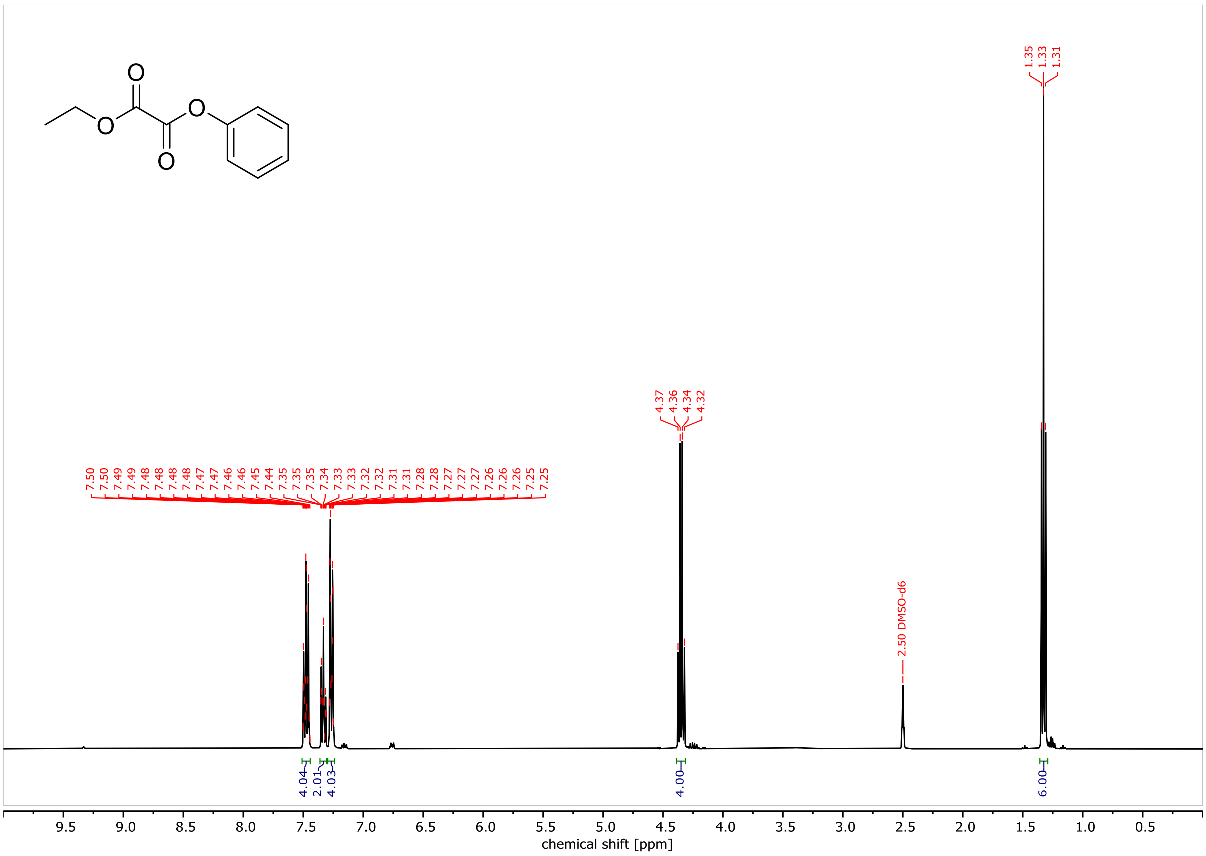
O
O
O
O
7.50
7.50
7.49
7.49
7.48
7.48
7.48
7.48
7.47
7.47
7.46
7.46
7.45
7.44
7.35
7.35
7.35
7.34
7.33
7.33
7.32
7.32
7.31
7.31
7.28
7.28
7.27
7.27
7.27
7.26
7.26
7.26
7.25
7.25
4.37
4.36
4.34
4.32
2.50 DMSO-d6
1.35
1.33
1.31
4.04
2.01
4.03
4.00
6.00
9.5 9.0 8.5 8.0 7.5 7.0 6.5 6.0 5.5 5.0 4.5 4.0 3.5 3.0 2.5 2.0 1.5 1.0 0.5
chemical shift [ppm]

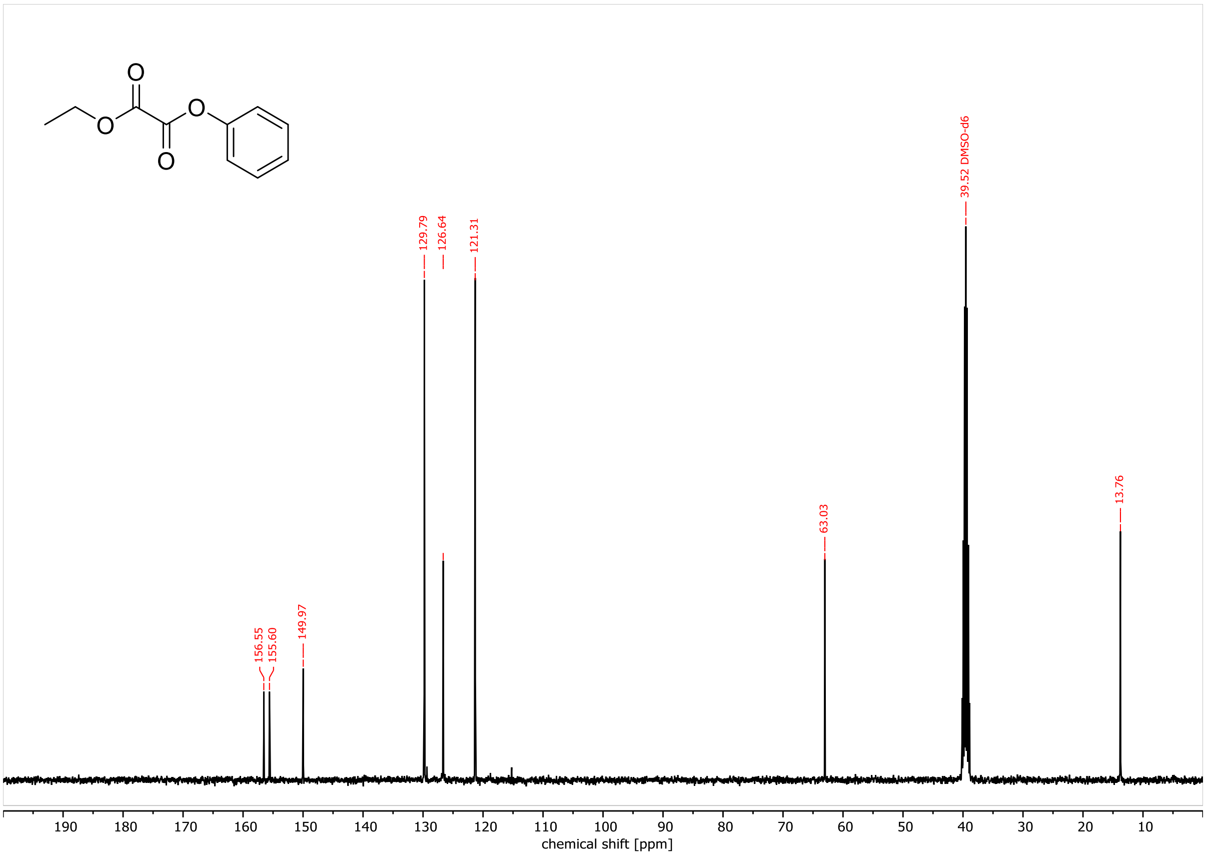
O
O
O
O
156.55
155.60
149.97
129.79
126.64
121.31
63.03
39.52 DMSO-d6
13.76
190 180 170 160 150 140 130 120 110 100 90 80 70 60 50 40 30 20 10
chemical shift [ppm]

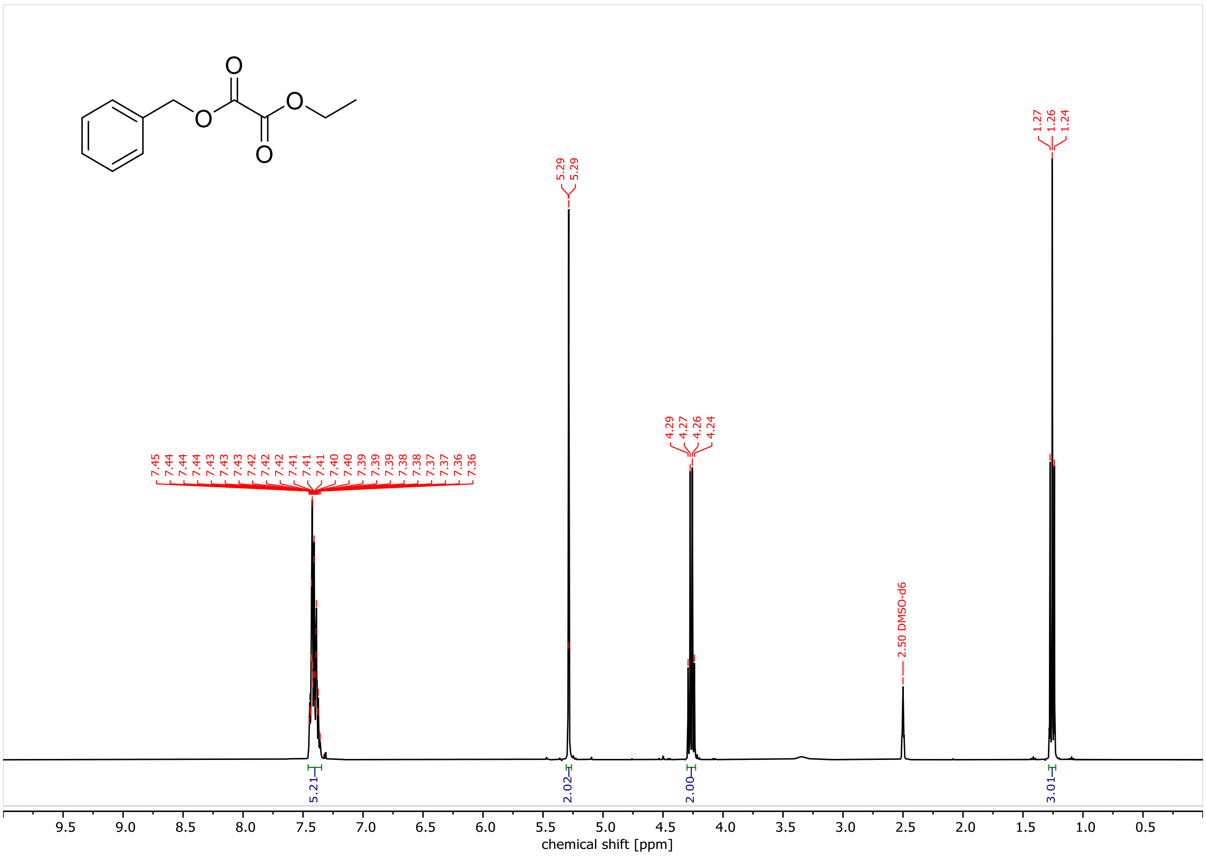

7.45
7.44
7.44
7.44
7.43
7.43
7.43
7.42
7.42
7.42
7.41
7.41
7.41
7.40
7.40
7.39
7.39
7.39
7.38
7.38
7.37
7.37
7.36
7.36
5.29
5.29
4.29
4.27
4.26
4.24
2.50 DMSO-d6
1.27
1.26
1.24
5.21
2.02
2.00
3.01
9.5
9.0
8.5
8.0
7.5
7.0
6.5
6.0
5.5
5.0
4.5
4.0
3.5
3.0
2.5
2.0
1.5
1.0
0.5
chemical shift [ppm]

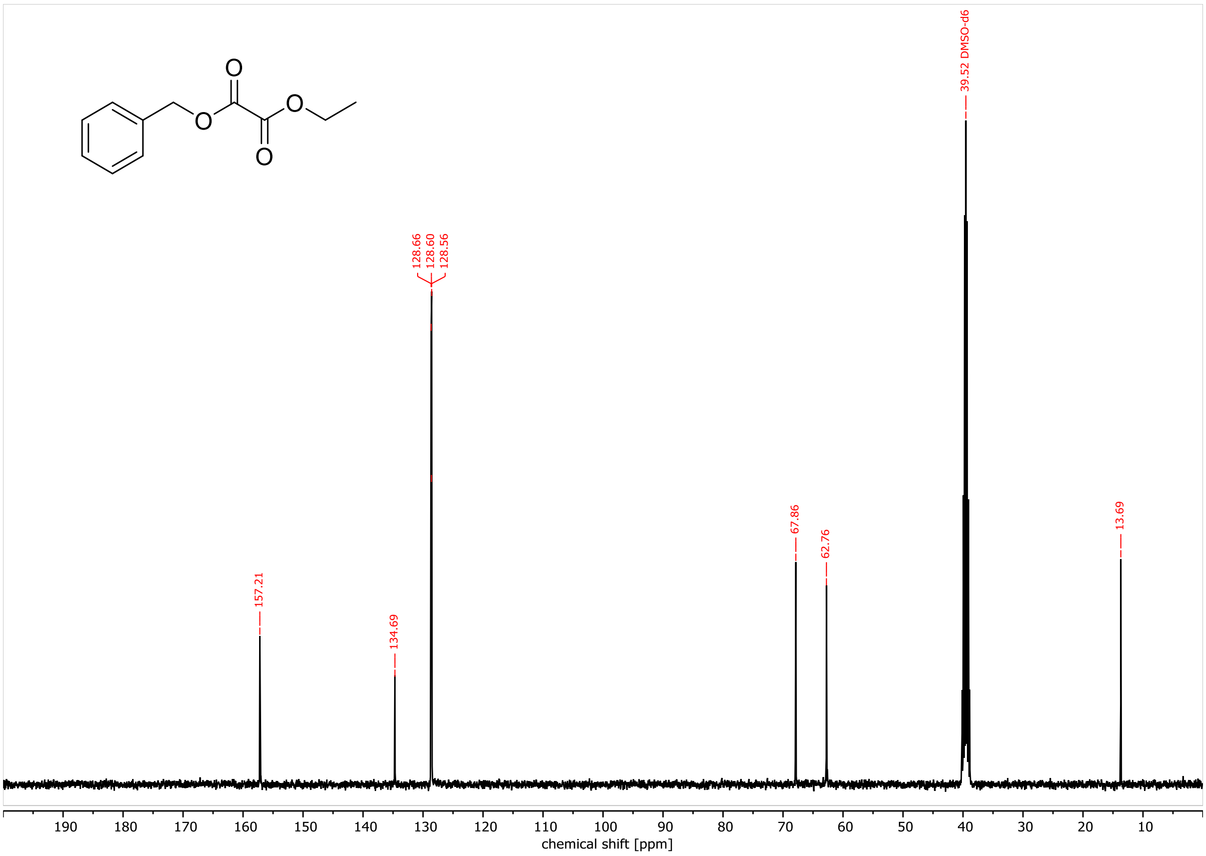

157.21
134.69
128.66
128.60
128.56
67.86
62.76
39.52 DMSO-d6
13.69
190
180
170
160
150
140
130
120
110
100
90
80
70
60
50
40
30
20
10
chemical shift [ppm]

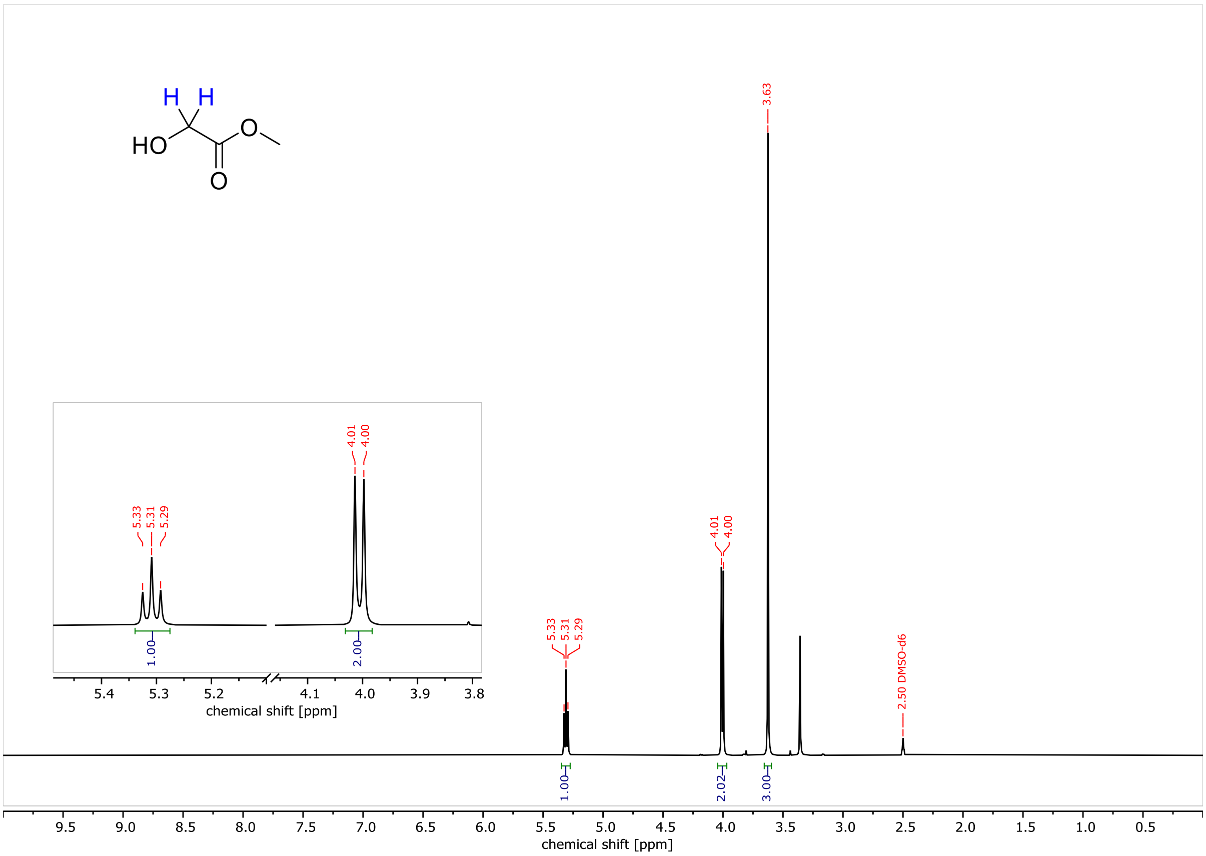
HO
O
O
H H
5.33
5.31
5.29
4.01
4.00
3.63
2.50 DMSO-d6
1.00
2.02
3.00
2.00
chemical shift [ppm]

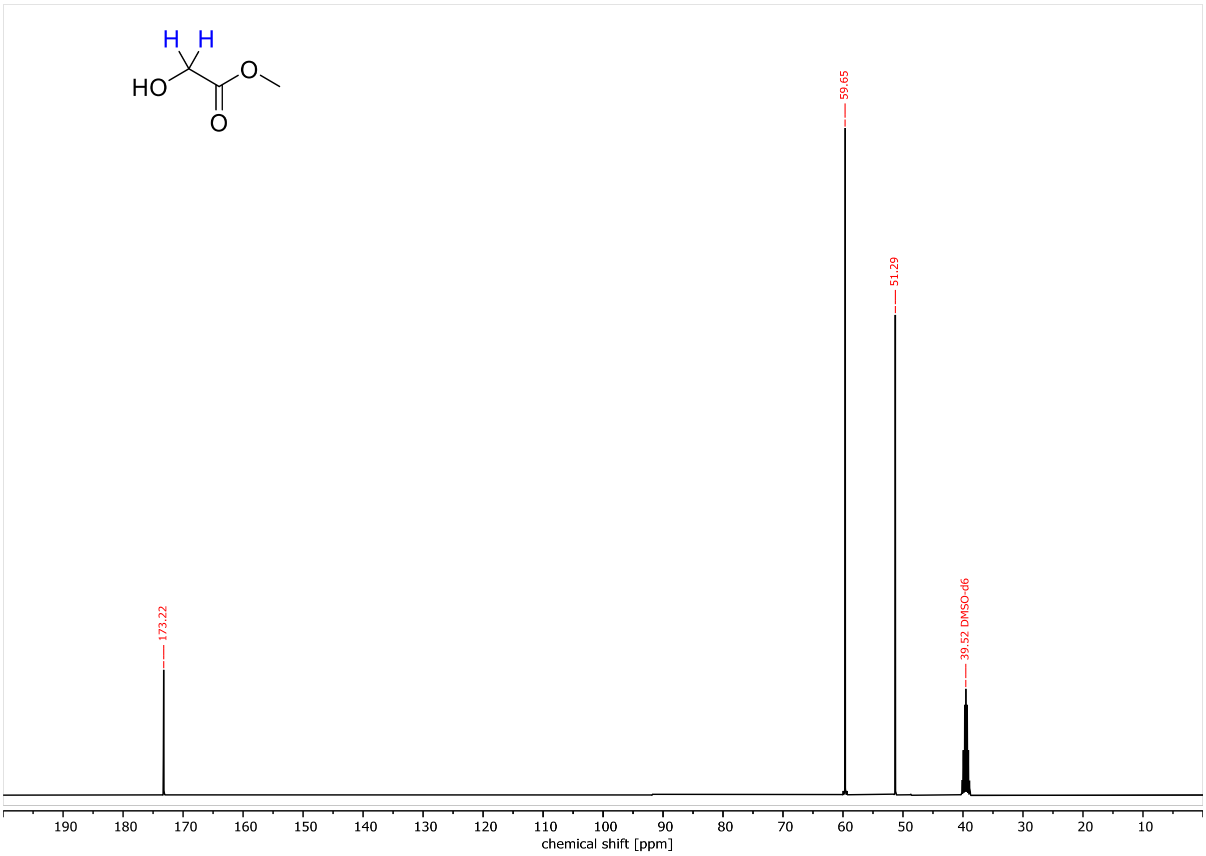
HO
O
O
H H
173.22
59.65
51.29
39.52 DMSO-d6
chemical shift [ppm]

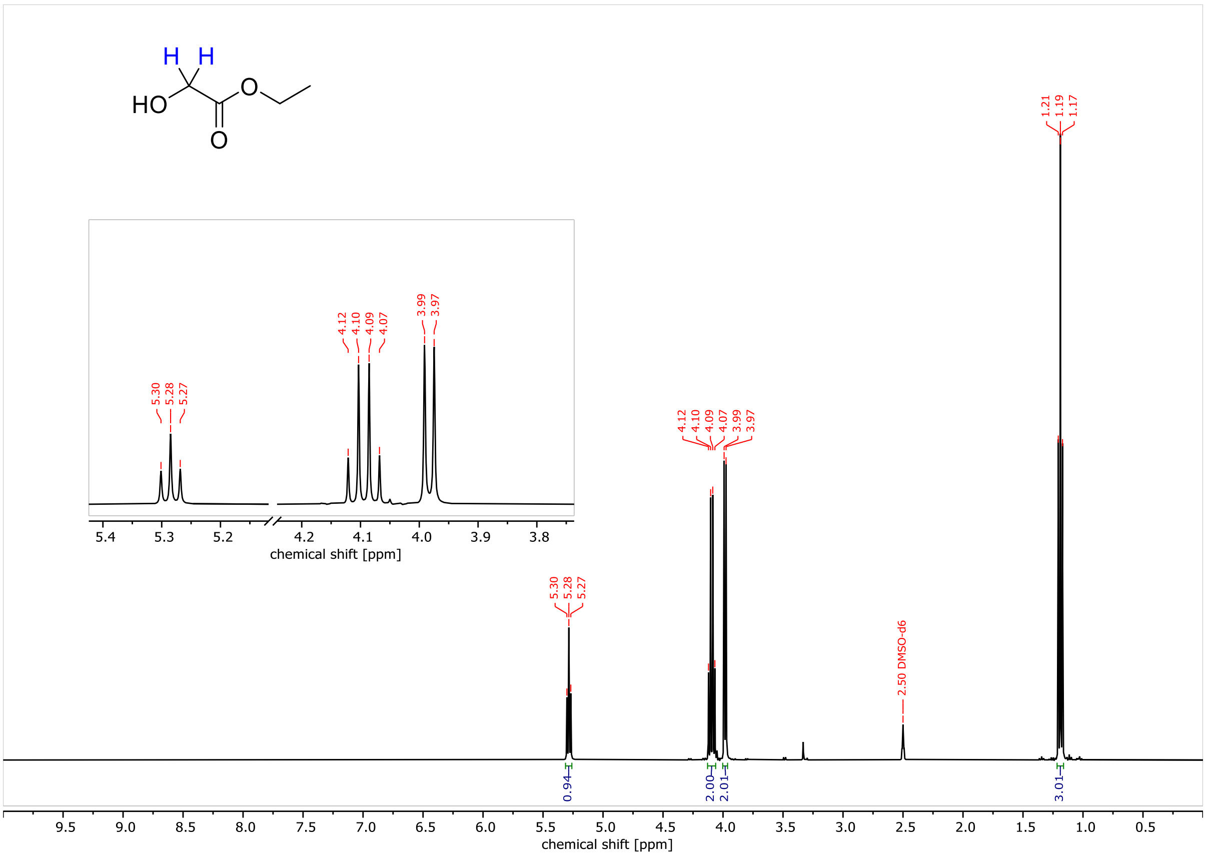
H H
HO
O
O
5.30
5.28
5.27
4.12
4.10
4.09
4.07
3.99
3.97
5.4
5.3
5.2
4.2
4.1
4.0
3.9
3.8
chemical shift [ppm]
2.50 DMSO-d6
1.21
1.19
1.17
0.94
2.00
2.01
3.01
9.5
9.0
8.5
8.0
7.5
7.0
6.5
6.0
5.5
5.0
4.5
4.0
3.5
3.0
2.5
2.0
1.5
1.0
0.5
chemical shift [ppm]

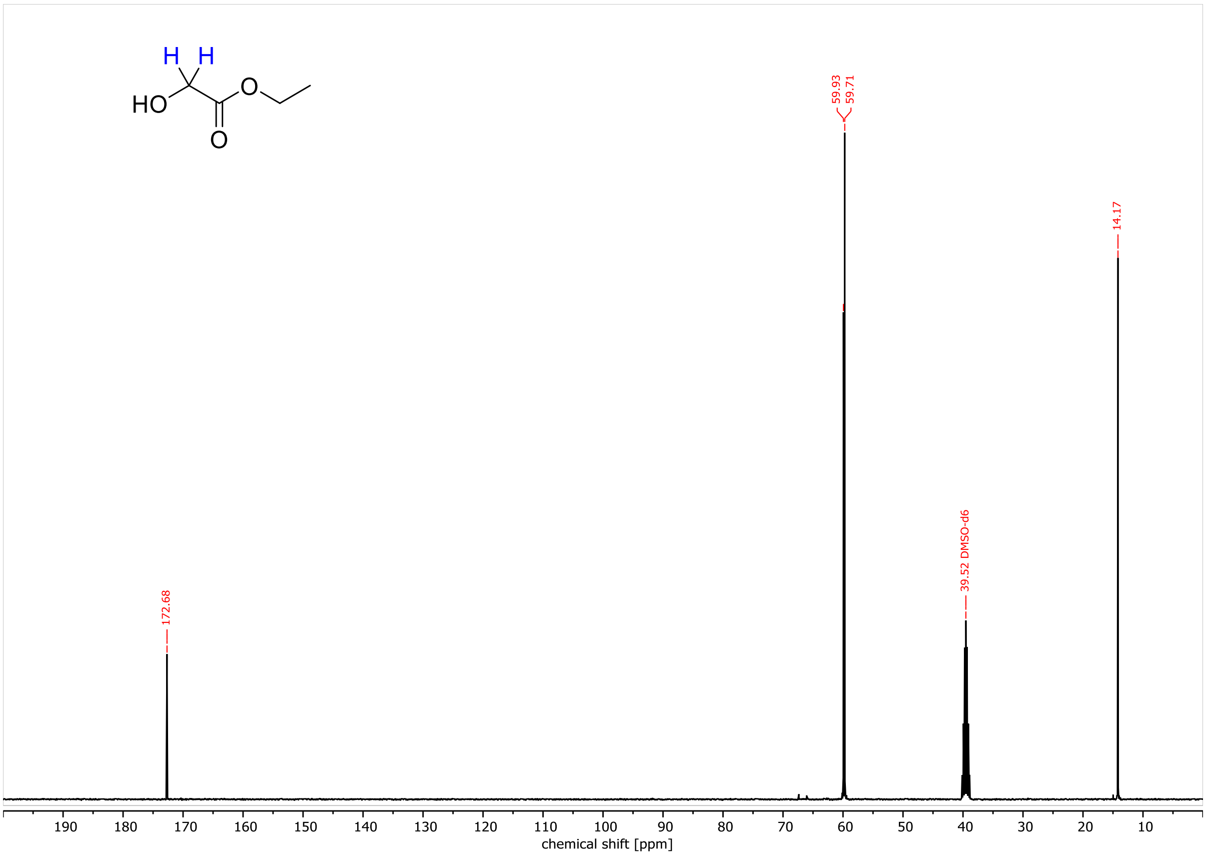
H H
HO
O
O
172.68
59.93
59.71
39.52 DMSO-d6
14.17
190
180
170
160
150
140
130
120
110
100
90
80
70
60
50
40
30
20
10
chemical shift [ppm]

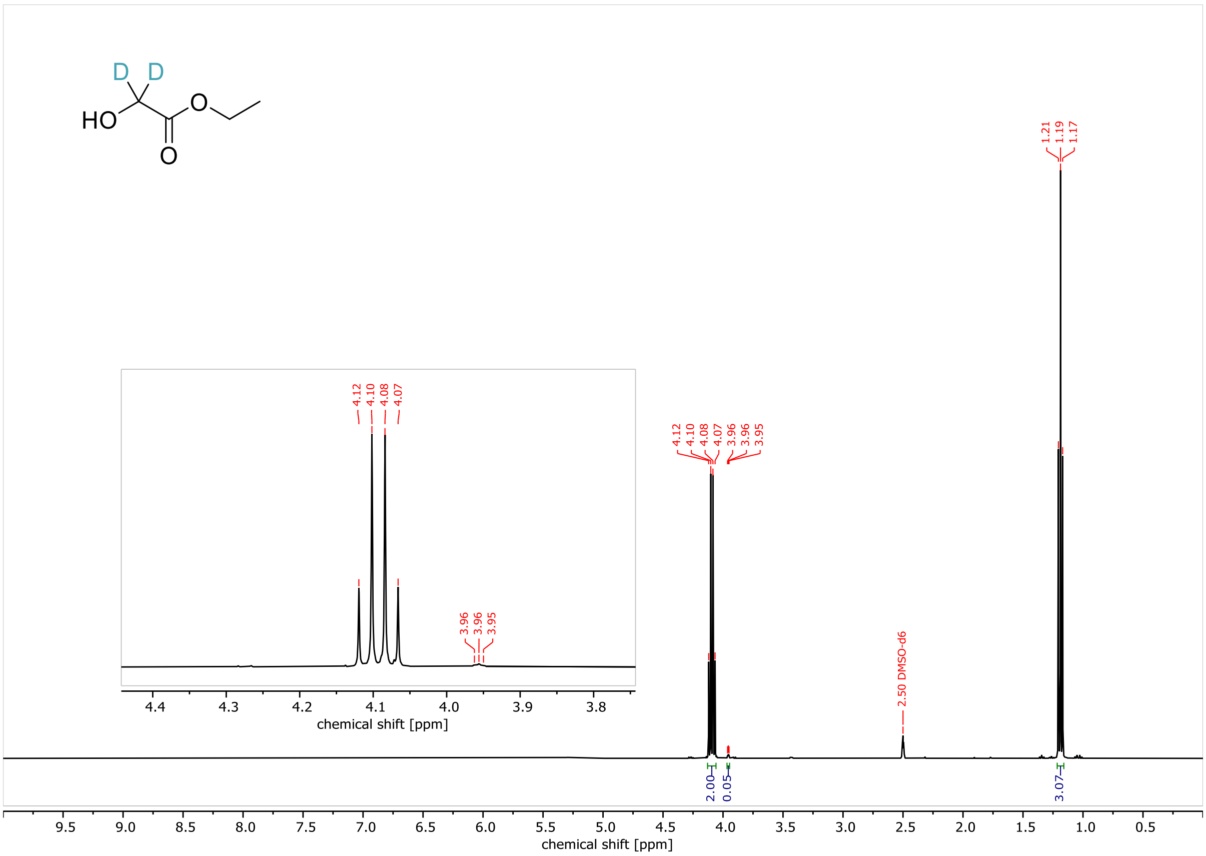
D D
HO
O
O
4.12 4.10 4.08 4.07
3.96 3.96 3.95
4.12 4.10 4.08 4.07 3.96 3.96 3.95
2.50 DMSO-d6
1.21 1.19 1.17
2.00
0.05
3.07
chemical shift [ppm]
chemical shift [ppm]

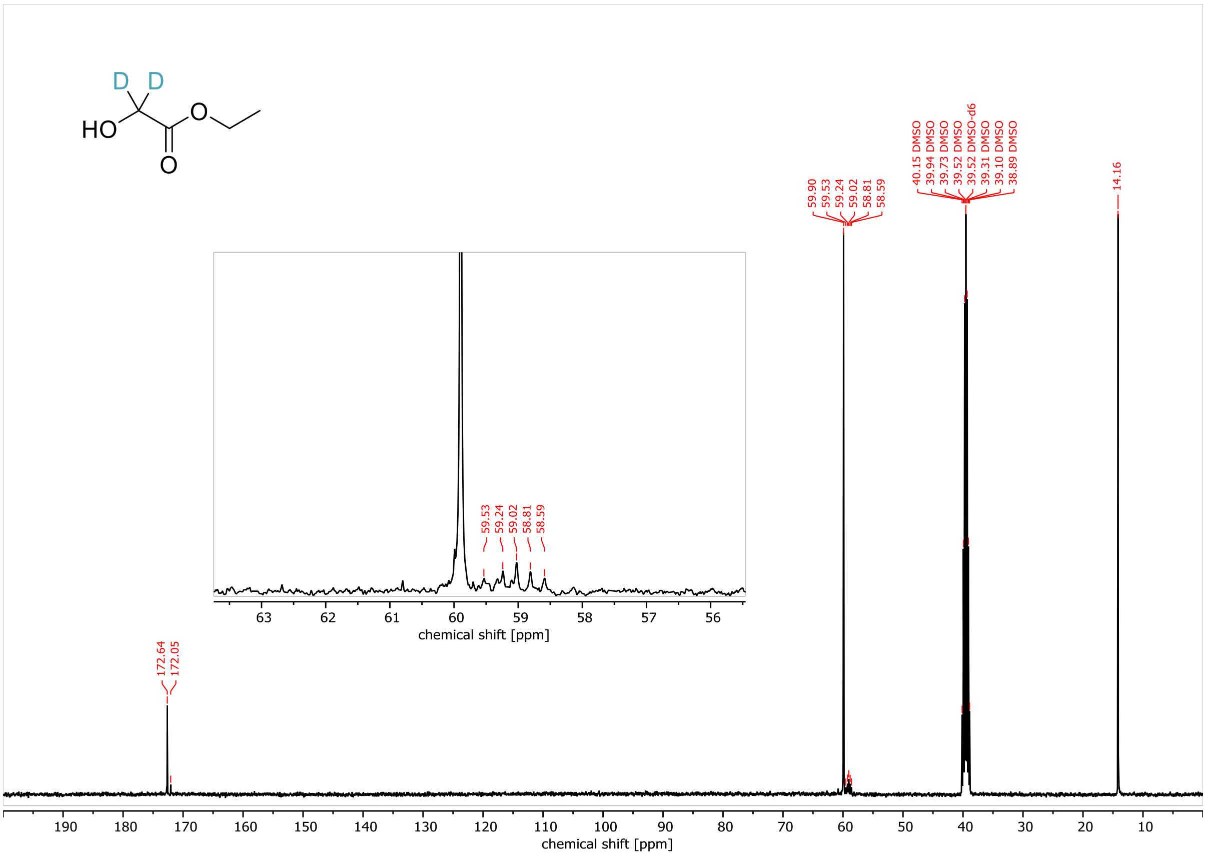
D D
HO
O
O
59.90 59.53 59.24 59.02 58.81 58.59
40.15 DMSO 39.94 DMSO 39.73 DMSO 39.52 DMSO 39.52 DMSO-d6 39.31 DMSO 39.10 DMSO 38.89 DMSO
14.16
59.53 59.24 59.02 58.81 58.59
172.64 172.05
chemical shift [ppm]
chemical shift [ppm]

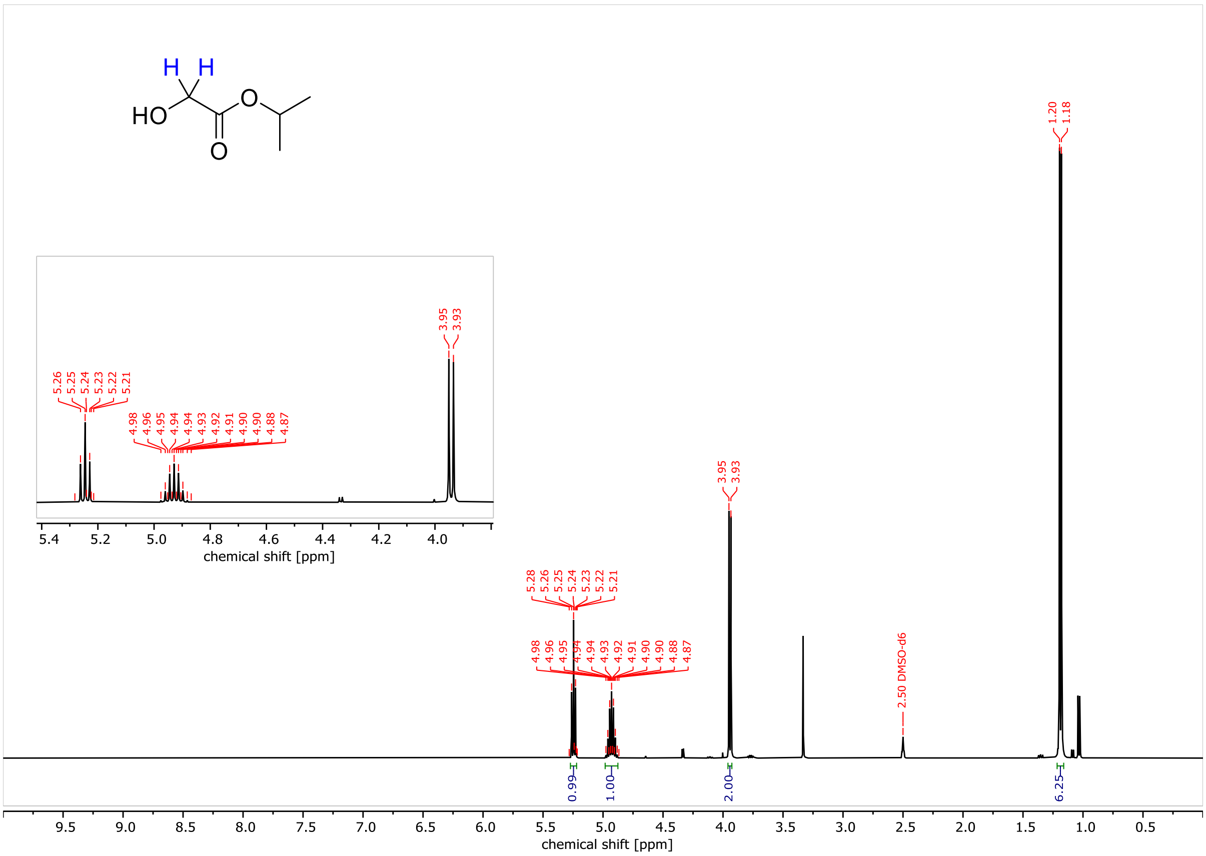
HO
O
O
H H
5.4
5.2
5.0
4.8
4.6
4.4
4.2
4.0
chemical shift [ppm]
3.95
3.93
2.50 DMSO-d6
1.20
1.18
0.99
1.00
2.00
6.25
9.5
9.0
8.5
8.0
7.5
7.0
6.5
6.0
5.5
5.0
4.5
4.0
3.5
3.0
2.5
2.0
1.5
1.0
0.5
chemical shift [ppm]

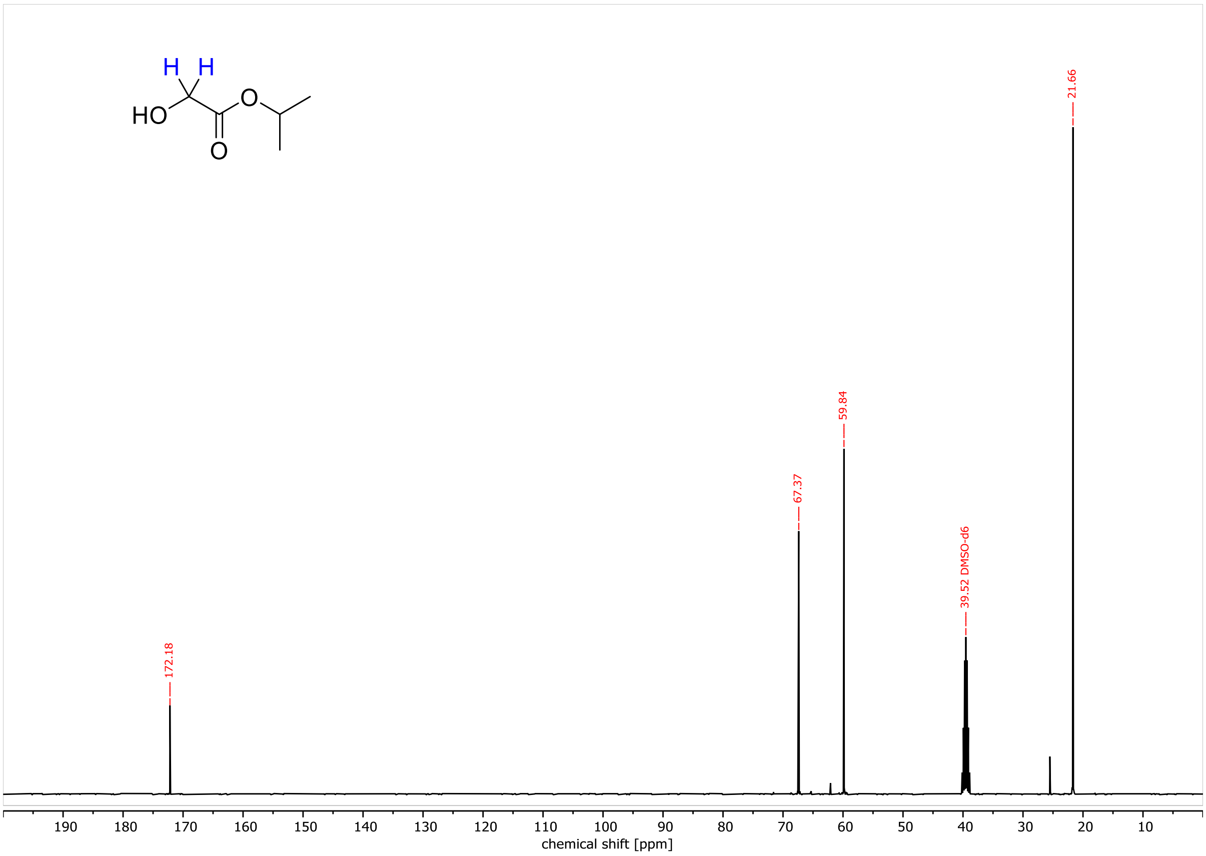
HO
O
O
H H
172.18
67.37
59.84
39.52 DMSO-d6
21.66
190
180
170
160
150
140
130
120
110
100
90
80
70
60
50
40
30
20
10
chemical shift [ppm]

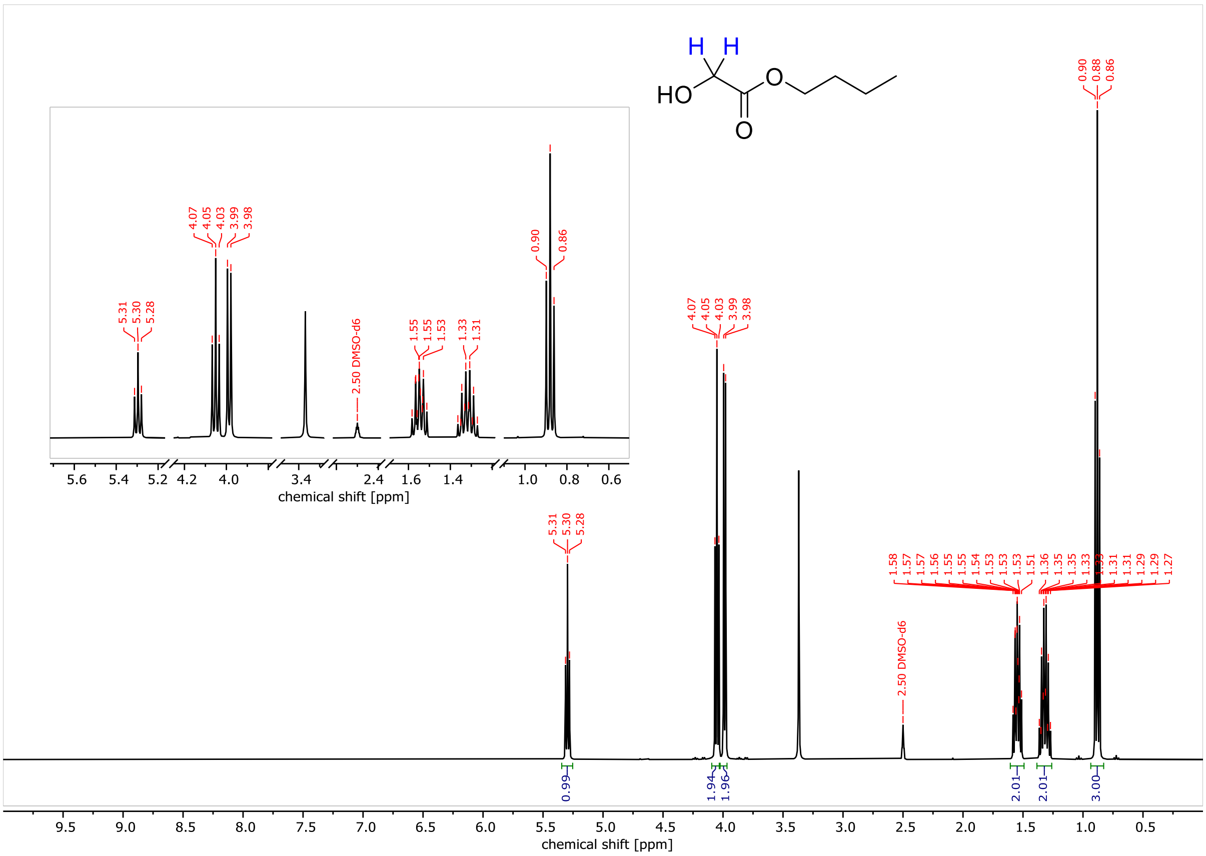
HO
O
O
H H
chemical shift [ppm]
2.50 DMSO-d6

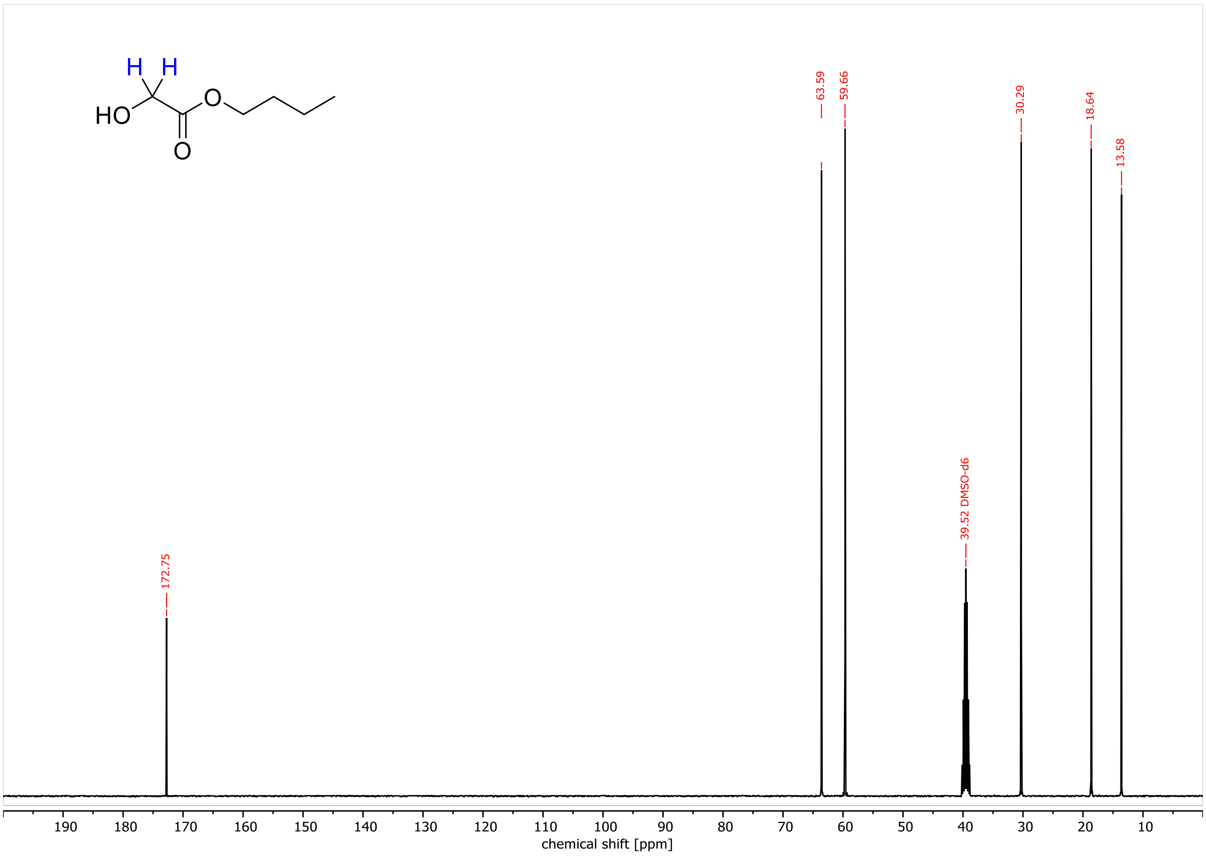
HO
O
O
H H
172.75
63.59
59.66
39.52 DMSO-d6
30.29
18.64
13.58
chemical shift [ppm]

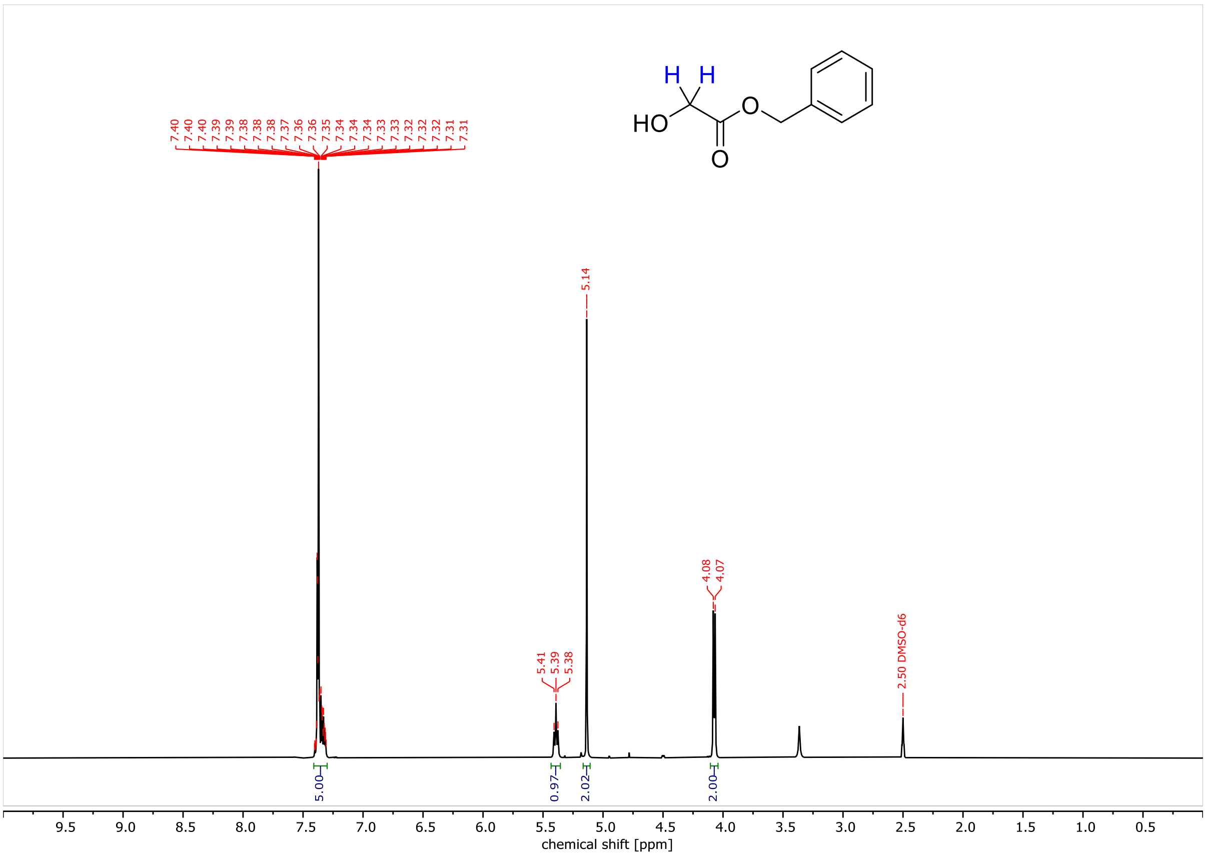
7.40
7.40
7.40
7.39
7.39
7.38
7.38
7.38
7.37
7.36
7.36
7.35
7.34
7.34
7.34
7.33
7.33
7.32
7.32
7.32
7.31
7.31
5.14
5.41
5.39
5.38
4.08
4.07
2.50 DMSO-d6
5.00
0.97
2.02
2.00
H H
HO
O
O
9.5 9.0 8.5 8.0 7.5 7.0 6.5 6.0 5.5 5.0 4.5 4.0 3.5 3.0 2.5 2.0 1.5 1.0 0.5
chemical shift [ppm]

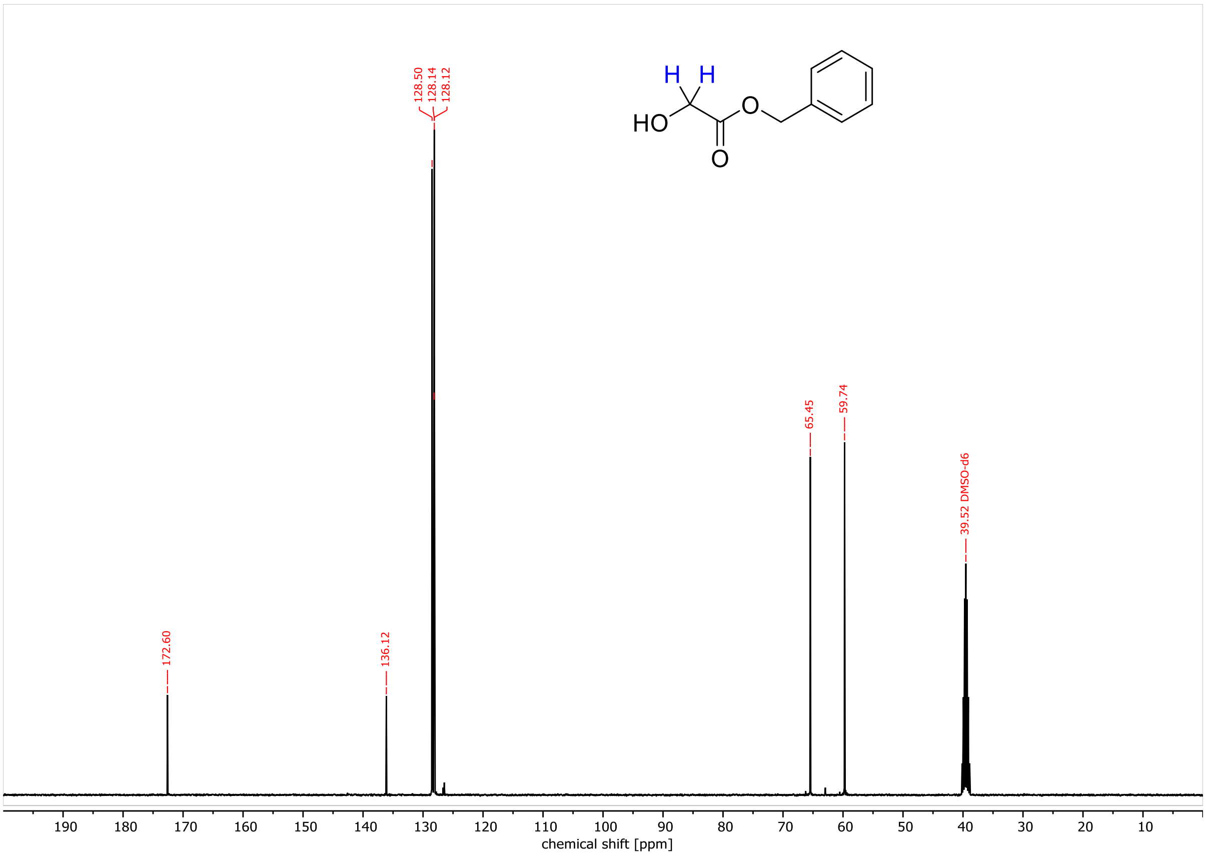
128.50
128.14
128.12
172.60
136.12
65.45
59.74
39.52 DMSO-d6
H H
HO
O
O
190 180 170 160 150 140 130 120 110 100 90 80 70 60 50 40 30 20 10
chemical shift [ppm]

Table S23: Data evaluation of the screening of different oxalic esters in batch type cells.

Entry	Starting Material-Oxalic ester	Chemical shift IStd [ppm]	Integral IStd	Chemical shift CH_2-group product [ppm]	Integral product	^{1}H NMR yield [%][a]	Isolated yield
**1**	**Methyl**	6.030	3.00	3.999	1.40	**70**	**40**
**2**	**Isopropyl**	6.024	3.00	3.937	1.84	**92**	**60**
**3**	**Butyl**	6.034	3.00	3.985	1.82	**91**	**64**
**4**	**Bis(3-methylbut-2-en-1-yl)**	6.032	3.00	3.975	1.68	**84**	
**5**	**Methoxyethyl**	6.029	3.00	4.006	1.89	**95**	
**6**	**2,2,2-Trifluoroethyl**	6.036	3.00	4.129	1.27	**64**	
**7**	**Menthyl**	6.047	3.00	3.981	1.60	**80**	
**8**	**Hexafluoroisopropyl**	6.060	3.00	-	-	**decomposed**	
**9**	**Phenyl**	6.049	3.00	4.296	1.67	**84**	
**10**	**4-Methoxyphenyl**	6.045	3.00	4.264	0.71	**36**[b]	
**11**	**Benzyl**	6.053	3.00	4.062	1.93	**96**	**64**
**12**	**4-Methylbenzyl**	6.045	3.00	4.035	1.79	**90**	
**13**	**4-Methoxybenzyl**	6.030	3.00	4.010	0.57	**29**[b]	
**14**	**4-Fluorobenzyl**	6.051	3.00	4.051	1.79	**90**	

[a] Quantification by **^{1}H NMR** using 1,3,5-trimethoxybenzene as internal standard (1.0 mmol, 168.2 mg). [b] Solubility issues (not suitable for flow electrolysis.)

Literature

[1] C. C. Gruber, G. Oberdorfer, C. V. Voss, J. M. Kremsner, C. O. Kappe, W. Kroutil, *J. Org. Chem.* **2007**, *72*, 5778–5783.
[2] A. Kütt, I. Leito, I. Kaljurand, L. Sooväli, V. M. Vlasov, L. M. Yagupolskii, I. A. Koppel, *J. Org. Chem.* **2006**, *71*, 2829–2838.
[3] Y. Morii, T. Watanabe, Y. Saga, T. Kambe, M. Kondo, S. Masaoka, *ChemElectroChem* **2024**, *11*, e202400061.
[4] M. C. Leech, K. Lam, *Accounts of Chemical Research* **2020**, *53*, 121–134.
[5] M. Ávila-Gutierrez, S. Gutierrez-Portocarrero, L. Corono-Elizarrarás, M. A. Alpuche Aviles, *Journal of the Mexican Chemical Society* **2023**, *67*, 393–402.
[6] K. Neubert, M. Hell, M. Chávez Morejón, F. Harnisch, *ChemSusChem* **2022**, *15*, e202201426.
[7] K. Čebular, B. Đ. Božić, S. Stavber, *Molecules* **2018**, *23*, 2235.
[8] A. Stergiou, A. Bariotaki, D. Kalaitzakis, I. Smonou, *The Journal of Organic Chemistry* **2013**, *78*, 7268-7273.
[9] Y. Morino, T. Yatabe, K. Suzuki, K. Yamaguchi, *Green Chem.* **2022**, *24*, 2017–2026.
[10] J. P. Ferris, J. D. Wos, A. P. Lobo, *J. Mol. Evol.* **1974**, *3*, 311–316.
[11] M. F. Grünberg, L. J. Gooßen, *Chem. - Eur. J.* **2013**, *19*, 7334–7337.
[12] P. M. Lahti, D. A. Modarelli, F. C. Rossitto, A. L. Inceli, A. S. Ichimura, S. Ivatury, *J. Org. Chem.* **1996**, *61*, 1730–1738.
[13] R. M. Denton, J. An, B. Adeniran, A. J. Blake, W. Lewis, A. M. Poulton, *J. Org. Chem.* **2011**, *76*, 6749–6767.

[14] W. S. Trahanovsky, C. C. Ong, J. A. Lawson, *J. Am. Chem. Soc.* **1968**, *90*, 2839–2842.
[15] R. Petrus, P. Fałat, P. Sobota, *Dalton Trans.* **2020**, *49*, 866–876.
[16] M. Peifer, R. Berger, V. W. Shurtleff, J. C. Conrad, D. W. C. MacMillan, *J. Am. Chem. Soc.* **2014**, *136*, 5900–5903.
[17] P. J. Geaneotes, C. P. Janosko, C. Afeke, A. Deiters, P. E. Floreancig, *Angew. Chem. Int. Ed.* **2024**, *63*, e202409229.
